# Diversity and Systematics of *Schizomavella* Species (Bryozoa: Bitectiporidae) from the Bathyal NE Atlantic

**DOI:** 10.1371/journal.pone.0139084

**Published:** 2015-10-21

**Authors:** Oscar Reverter-Gil, Björn Berning, Javier Souto

**Affiliations:** 1 Departamento de Zooloxía e Antropoloxía Física, Facultade de Bioloxía, Universidade de Santiago de Compostela, 15782 Santiago de Compostela, Spain; 2 Geowissenschaftliche Sammlungen, Oberösterreichisches Landesmuseum, Welser Str. 20, 4060 Leonding, Austria; 3 Institut für Paläontologie, Fakultät für Geowissenschaften, Geographie und Astronomie, Geozentrum, Universität Wien, Althanstrasse 14, 1090, Wien, Austria; Australian Museum, AUSTRALIA

## Abstract

Eight NE Atlantic and Mediterranean species, which were originally assigned to the genus *Schizoporella* (Family Schizoporellidae) when introduced, are redescribed and stabilized by typification. Seven of these species are transferred to the bitectiporid genus *Schizomavella*: *S*. *fischeri*, *S*. *glebula*, *S*. *neptuni*, *S*. *obsoleta*, *S*. *richardi*, *S*. *triaviculata*, and *S*. *triaviculata* var. *paucimandibulata*, which is here raised to species rank. The eighth species, *Schizoporella fayalensis*, is transferred to the lanceoporid genus *Stephanotheca*. *Schizomavella obsoleta* and *S*. *glebula* are considered junior subjective synonyms of *S*. *fischeri* and *S*. *richardi*, respectively. Two new species are described: *Schizomavella rectangularis* n. sp. from the Strait of Gibraltar, and *Schizomavella phterocopa* n. sp. from the Great Meteor Bank. A new subgenus, *Calvetomavella* n. subgen. is established as a result of a phylogenetic analysis based on morphological characters; it includes *S*. *neptuni*, *S*. *triaviculata*, *S*. *paucimandibulata* and *S*. *phterocopa* n. sp., together with *Schizomavella discoidea* and *Schizomavella noronhai*. The rest of the species remain in the nominotypical subgenus *Schizomavella*.

## Introduction

During the end of the 19^th^ century and the begining of the 20^th^, several French and Monacan vessels collected samples during cruises mainly in the Northeast Atlantic: *Travailleur*, *Talisman*, *l’Hirondelle* and *Princesse Alice*. The results on bryozoans were published by Jullien [1,2], Jullien & Calvet [3] and Calvet [4,5,6,7]. In total, more than 150 species were newly described, although most of these were never redescribed according to current standards. Thirteen of the new species were initially assigned to the then ill-defined, genus *Schizoporella*. While their generic assignment has been a matter of some debate over time, in the present paper we revise seven of these species. Whereas seven species are placed in the bitectiporid genus *Schizomavella* Canu & Bassler, 1917, one is transferred to the lanceoporid genus *Stephanotheca* Reverter-Gil, Souto & Fernández-Pulpeiro, 2012.

*Schizomavella* is a speciose genus with about 43 recent species reported from all over the world [8,9]. Some 11 species seem to be restricted to the North Atlantic. Another 11 species are supposedly endemic to the Mediterranean Sea. A further four species supposedly occur in both the Atlantic and the Mediterranean. However, the identity of many previous records is not always clear owing to the widespread confusion surrounding the diagnostic characters of some of the species. Although several species have been newly described in recent years, both in the Atlantic [10,11] and in the Mediterranean [9,12,13], and some previously known species were redescribed [10,13,14,15,16], the true diversity of the genus *Schizomavella* can still be regarded as poorly known. Moreover, the morphology of its species is not yet well understood and the genus is therefore ill defined. For instance, some species previously classified as belonging to the genus *Schizomavella* have recently been redescribed and assigned to a new genus, *Stephanotheca*, owing to differences in the structure of the ovicell and its closure type [17].

Three of the species described in the present paper (*Schizoporella fischeri*, *Schizoporella obsoleta* and *Schizoporella neptuni*) were originally described by Jullien [1]. *Schizoporella glebula* was introduced by Jullien & Calvet [3]. Another two (*Schizoporella richardi* and *Schizoporella triaviculata*) were described by Calvet [3]. The species introduced as *Schizoporella fayalensis* by Calvet [3], is here placed in the lanceoporid genus *Stephanotheca*. Another species was first described by Calvet [6] as an unnamed variety of *S*. *triaviculata*, and later named as variety *paucimandibulata* by d’Hondt [18]. Finally, two new species are introduced in the present paper. One of them, similar to *S*. *triaviculata paucimandibulata*, is from material newly collected at the Great Meteor Bank (central North Atlantic). The other species was collected near the Strait of Gibraltar and was originally labelled as *S*. *neptuni*.

The aim of the present study is to revise this group of neglected species that occur in the bathyal Atlantic and Mediterranean Sea. As these species were not associated with the genus *Schizomavella* for most of their history, we analyse their phylogenetic relationships within the group, and evaluate their impact on the generic status of *Schizomavella*.

## Material and Methods

### Specimens examined

Previous records of the studied species were compiled from the literature. The longitudes of the sampling stations of the *Talisman*, *Travailleur* and *l’Hirondelle* cruises, published by Jullien [1,2], Jullien & Calvet [3] and Calvet [6], were initially measured with reference to the Paris meridian. They have been here corrected to the Greenwich meridian (see Ryland, 1969: 238) [19].

There has been some confusion about the year of publication of the results of the *Travailleur* cruise by Jullien– 1882 or 1883 according to different authors. The source of the problem seems to be that the work was actually published twice. The results first appeared independently in 1882, as an extract of volume 7 of the *Bulletin de la Société Zoologique de France* corresponding to the year 1882, with pages numbered 1–33 [20]. Subsequently, the paper was published again with the entire volume 7 of the Bulletin during the first months of 1883 (J.-L. d’Hondt, pers. comm.). In this paper, the sentence “*(Séance du 26 décembre 1882)*” was added to the title, and pages were numbered 497–534. Therefore, as the paper was first published in 1882, all the taxa described there by Jullien must be referred to this year, following the original pagination.

The authorship of taxa introduced in Jullien & Calvet [3] has been also matter of some confusion. The work is divided into two parts, the first one traditionally regarded as authored only by J. Jullien, and the second one by L. Calvet. While this seems true for the second part, because Calvet clearly published material Jullien did not see, the first part is actually arguably authored by both Jullien and Calvet, as stated in p. 11 of the work [3]. Moreover, it seems clear that Calvet was much more actively involved in establishing species in the first part than can be guessed from his humility in the second part when he cited Jullien as only author of the species introduced in the first part. Calvet has revised all of Jullien’s specimens and transferred some of these to his own collection at the Musée océanographique de Monaco, he actively chose colonies for imaging that were not from Jullien’s original suite of specimens, and even changed species names that were originally proposed by Jullien [21]. Consequently, the taxa introduced in the first part of the work should have “Jullien & Calvet” as author, while taxa from the second part should remain being cited as “Calvet *in* Jullien & Calvet”.

More than 135 specimens, including type and non-type material, stored in the following museums have been studied: Muséum National d’Histoire Naturelle, Paris (MNHN), Musée Océanographique, Monaco (MOM), Natural History Museum, London (NHMUK), Oberösterreichisches Landesmuseum, Linz (OLL; collection "Evertebrata varia" at the Biology Centre), Senckenberg Forschungsinstitut und Naturmuseum, Frankfurt (SMF), and Museu Nacional de História Natural e da Ciência, Lisbon (MB).

We have also studied newly collected material from sampling surveys with the German RV *Meteor* to the Great Meteor Bank in 1970 (cruise M19) and 1998 (cruise M42/3); specimens from a settlement panel experiment conducted by M. Wisshak (Senckenberg am Meer, Wilhelmshaven) between the islands of Pico and Faial [22]; material collected during the sampling surveys *BANGAL 2011* and *Avilés 0511 (INDEMARES* project); material from the personal collection of J.-G. Harmelin (JGH), collected during different sampling surveys; as well as material from the personal collection of ORG.

When specifying geographic regions, abbreviations of the geographic directions (e.g. north = N, northeast = NE, etc.) are used in combination with the respective region (e.g. NE Atlantic).

### Morphological and phylogenetic analysis

The samples were examined with a stereomicroscope and uncoated material was photographed with a Zeiss EVO LS15 SEM, a LEO 1455VP SEM and an FEI Inspect S50 SEM, using the back-scattered electron detector in low vacuum mode. Images of sputter-coated specimens of *Schizomavella phterocopa* were taken with a CamScan (Serie-2-CS-44). Measurements were taken with the software ImageJ^®^ on the SEM photographs. Pore density (PD) was measured by randomly placing a digital square with a side length of 200 μm over a photo of the frontal wall of an autozooid and counting the number of pores that lay completely within this square. The resulting number therefore represents the quantity of pores/0.04mm^2^.

A matrix of 33 morphological characters (S1 Text, S1 Table) was created and treated following Maximum Parsimony (MP) and Bayesian Inference (BI) principles to investigate the phylogenetic relationships of *Schizomavella* species. Branch and Bound search with tree bisection and reconnection option was used for MP in PAUP 4.010 [23], and branch support evaluated through 1000 bootstrap replicates. Two species, the lanceoporid *Stephanotheca fayalensis* (Calvet *in* Jullien & Calvet, 1903), and *Hippoporina teresae* Souto et al., 2010, which is a bitectiporid as is *Schizomavella*, were included in the matrix as outgroups. A model including a variable codification and gamma parameter was selected for the BI. Five million generations were performed in two parallel runs in MrBayes 3.2.1 [24,25], sampling one tree every 1000 generations; the first 25% of the trees were discarded after inspection to confirm approaches to stationarity as burn-in, and the support evaluated through posterior probabilities.

### Nomenclatural acts

The electronic edition of this article conforms to the requirements of the amended International Code of Zoological Nomenclature, and hence the new names contained herein are available under that Code from the electronic edition of this article. This published work and the nomenclatural acts it contains have been registered in ZooBank, the online registration system for the ICZN. The ZooBank LSIDs (Life Science Identifiers) can be resolved and the associated information viewed through any standard web browser by appending the LSID to the prefix "http://zoobank.org/". The LSID for this publication is: urn:lsid:zoobank.org:pub:A56CB748-0718-46C5-AD7E-5A8D9401C79D. The electronic edition of this work was published in a journal with an ISSN, and has been archived and is available from the following digital repositories: PubMed Central, LOCKSS.

## Results

Superfamily **SMITTINOIDEA** Levinsen, 1909

Family **BITECTIPORIDAE** MacGillivray, 1895

Genus ***Schizomavella*** Canu & Bassler, 1917

Type species: *Lepralia auriculata* Hassall, 1842, by original designation.

*Diagnosis*: Colony encrusting, unilaminar to multilaminar; sheet-like or developing folded plates, mamillate growths, or massive and nodular; colony growth predominantly by zooidal budding, occasionally by frontal budding. Autozooids with cryptocystidean frontal shield, perforated by a highly variable number of small pseudopores, from very numerous to extremely reduced or almost absent, plus a row of peripheral areolar pores. Communication via small uniporous septula. Primary orifice sinuate; condyles present, often pronounced. Oral spines present though often lost during ontogeny or absent from adult zooids in some species. Avicularia adventitious, monomorphic or polymorphic, suboral or lateral to orifice. Ovicell hyperstomial (i.e. prominent), often becoming subimmersed by secondary calcification during ontogeny, completely calcified ectooecium with conspicuous frontal pseudopores, not closed by autozooidal operculum. Ancestrula tatiform or with a well-developed proximal cryptocyst forming a pseudosinus.

*Remarks*: The generic position of some species currently placed in the genus *Schizomavella*, as for instance *Schizoporella fischeri* Jullien, 1882, *Schizoporella neptuni* Jullien, 1882 and *Lepralia discoidea* Busk, 1859, has been a matter of debate for over 130 years. In his description of *S*. *neptuni*, Jullien [1] argued that some of its characters may justify the introduction of a new genus, separating it from the then large genus *Schizoporella*. For species with a suboral avicularium, Canu & Bassler [26] introduced the genus *Schizomavella*, with *Lepralia auriculata* Hassall, 1842 as genotype. The presence of two avicularia is presumably the reason why *S*. *neptuni* was still considered to belong to *Schizoporella* by e.g. d’Hondt [27] or Hayward [28]. Gordon [29] and Harmelin & d’Hondt [30] considered that *S*. *fischeri* and *S*. *neptuni* display some characters intermediate between *Schizoporella* and *Schizomavella*, but their suggestion followed the erroneous assumption of Canu & Bassler [26] that in *Schizomavella* the operculum closes the ovicell [17]. Moreover, the lepralielliform ovicell [31], in which the ectooecium is entirely calcified though perforated by numerous pseudopores, precludes these species from being affiliated with taxa having a microporelliform ovicell, in which the ectooecium is uncalcified.

Over time, additional new taxa have been ascribed to *Schizomavella* that were distinctly different from the characters of its type species, extremely stretching the generic diagnosis. In an attempt to bring some order into chaos, Gordon & d’Hondt [32] erected the genus *Parkermavella* for *Schizomavella*-like species with an imperforate frontal shield and only marginal areolae. Reverter-Gil & Fernández-Pulpeiro [11] established two groups in the genus *Schizomavella* based mainly on the morphology of the orifice and ovicell, but preferred not to include *S*. *discoidea* in either of them. Later, López de la Cuadra & García Gómez [14] discussed the similarities between *S*. *discoidea* and *S*. *neptuni*, and stated that they “could constitute a clade within *Schizomavella*”. Finally, Reverter-Gil et al. [17] stressed the importance of the ovicell in the classification of *Schizomavella* species and erected the new genus *Stephanotheca* for those in which the ovicell is closed by the operculum. Other species, like *Schizoporella triaviculata* Calvet *in* Jullien & Calvet, 1903 or *Schizoporella triaviculata* var. *paucimandibulata* d’Hondt, 1975, have never been thoroughly redescribed or systematically reassigned since their original descriptions, and were not taken into account in previous systematic analyses of the genus *Schizomavella*.

Although the species with (sub)cleithral ovicells have been transferred to the genus *Stephanotheca*, there are still some differences in ovicell morphology between the remaining *Schizomavella* species. In the genotype, *Lepralia auriculata* the acleithral ooecium is marginally covered by a thick imperforate layer of secondary calcification, resulting in a reduced proximal area of exposed pseudoporous ectooecium; the secondary calcification never covers this exposed ectooecium, even in heavily calcified zooids. This morphology is present in most other species of the genus, with some variation regarding the shape and extent of the exposed ectooecium as well as the number of pores and their size and shape.

In other European species of the genus, as for instance *S*. *grandiporosa* Canu & Bassler, 1925, *S*. *sarniensis* Hayward & Thorpe, 1995 and *S*. *teresae* Reverter-Gil & Fernández-Pulpeiro, 1996, together with *S*. *rectangularis* n. sp. (see below) and two new species from the Adriatic [9], the entire ectooecium is randomly perforated by pseudopores, and later almost completely covered by a thick, nodular secondary calcification that does not occlude the pseudopores. As a result, the porous ooecium becomes quite immersed, with an appearance identical to the autozooidal frontal shield.

In other species treated here, as for instance *S*. *neptuni* or *S*. *triaviculata* (see below), the ovicell only has a narrow, peripheral rim of imperforate secondary calcification, while most of its surface is a large area of exposed pseudoporous ectooecium; however, the ectooecium may be covered later by a very thin and smooth layer of secondary calcification that does not occlude the ectooecial pseudopores. Consequently, the ovicell is always prominent and the appearance of its surface is different to that of the frontal shield of autozooids.

**Fig 1:**
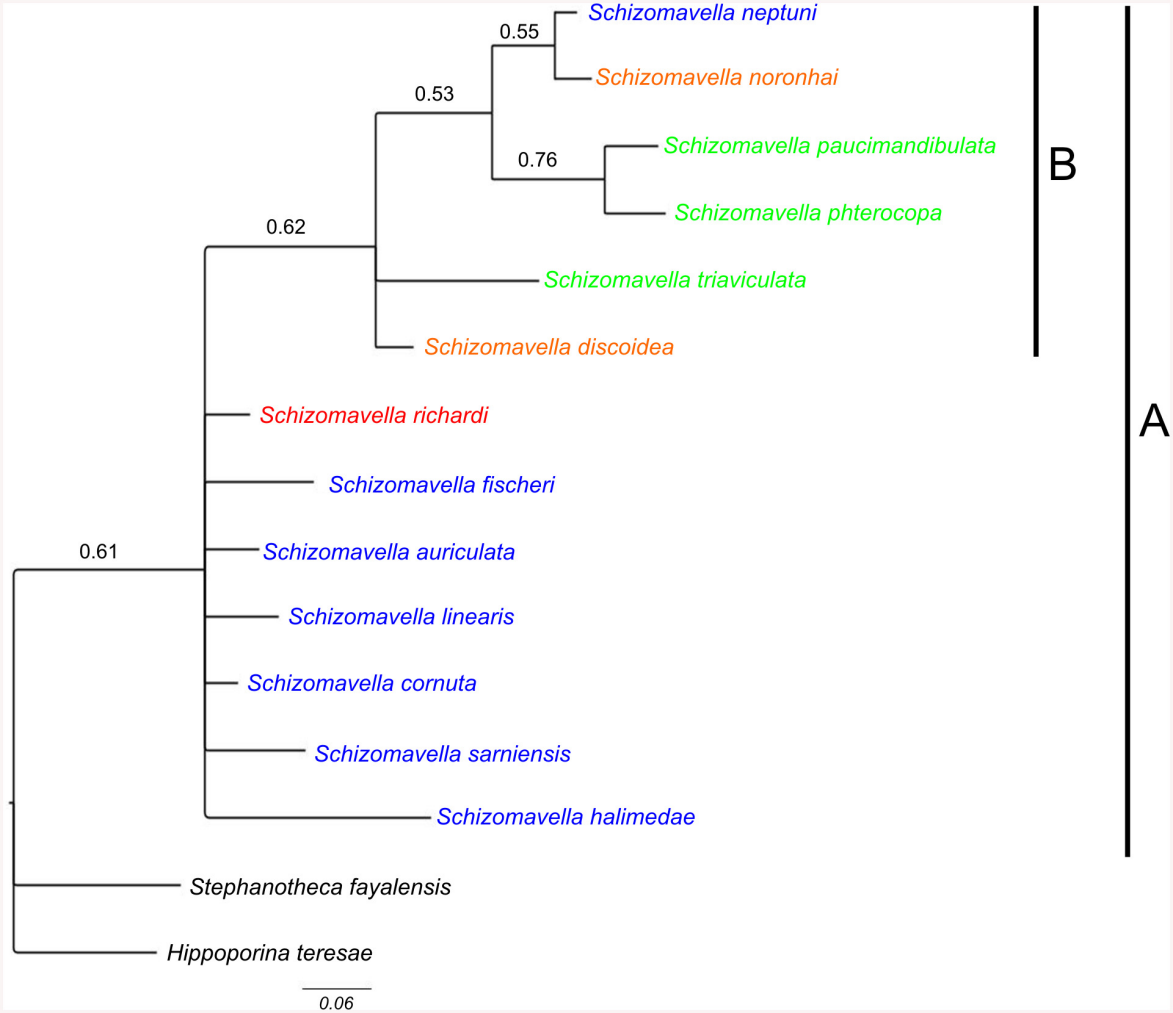
Phylogenetic relationships of *Schizomavella* species based on morphological characters. Maximum clade credibility tree; values above branches represent BI posterior probabalities. (blue: Continetal shelf distribution; orange: Madeira Island distribution; green: Azores Island distribution, red: Azores and continental shelf) A: genus *Schizomavella*; B: subgenus *Calvetomavella* n. subgen.

The ovicell morphology, used in combination with other diagnostic characters (e.g. the number and position of avicularia, number and persistence of oral spines, secondary calcification of the frontal wall), allows splitting the genus *Schizomavella* in two groups (Fig 1): the nominotypical subgenus including the genotype and most of the other species (*Schizomavella*), and another subgenus for species in which most of the ectooecium is exposed (*Calvetomavella* n. subgen.).

Species of *Calvetomavella* n. subgen. also have two distal avicularia, although sometimes one of them can be lacking, or occasionally some zooids have only a single suboral avicularium—especially in smaller zooids. Furthermore, the avicularian rostrum in these species is often pointed. In the subgenus *Schizomavella* the avicularium is defined as single and suboral. Only in *Schizomavella linearis* (Hassall, 1841) and *S*. *rectangularis* n. sp. (see below) the avicularia are double, one being placed on each side of the orifice. Moreover, in this group the avicularia always have rounded rostra [with the exception of *Schizomavella fischeri* (Jullien, 1882)]. The spines in most species of *Calvetomavella* n. subgen. are numerous and persist throughout ontogeny, and at least one pair is visible also in ovicellate zooids; however, in *S*. *triaviculata* (and also in *L*. *discoidea*) spines are absent in ovicellate zooids due to the development of a peristome. The type species of the (sub)genus *Schizomavella*, *S*. *auriculata*, and most of the other species of the genus, also have oral spines, although generally fewer than 5 are present in young, peripheral zooids, and these are absent from ovicellate zooids. Exceptions to the rule are *S*. *fischeri*, *S*. *richardi* and *S*. *halimedae* (Gautier, 1955).

The most important difference between the two groups is the morphology of the ovicell. However, some of the characters may overlap between both groups, as stated above: some species of *Schizomavella s*.*s*. also have uniformly perforated ovicells, though covered by thick secondary calcification; moreover, they have a reduced number of oral spines, lacking in most zooids, and a single suboral avicularium. A great number of spines, some of them persisting in ovicellate zooids, are also present in *S*. *fischeri* and *S*. *richardi*. And, finally, a pair of distal avicularia is also present in *S*. *linearis* and *S*. *rectangularis* n. sp. For these reasons, we prefer to define the present groups as subgenera within *Schizomavella* rather than as distinct genera.

The decision to introducing subgenera for the two groups is only partly supported by cladistic analysis. Maximum parsimony resolved only two clades although these are not well supported, one comprises by *S*. *triaviculata*, *S*. *paucimandibulata* and *S*. *phterocopa* (bootstrap value = 52%), and the other comprises only *S*. *paucimandibulata* and *S*. *phterocopa* (bootstrap value = 57%). However, the erection of the new subgenus *Calvetomavella* receives some support in the BI analysis (Fig 1), which shows a weakly supported clade (PP = 0.62) that includes *S*. *neptuni*, *S*. *noronhai*, *S*. *paucimandibulata*, *S*. *phterocopa*, *S*. *triaviculata* and *S*. *discoidea*. The relationships between the new subgenus and the rest of the analyzed species were not clearly established in either analysis. In particular, the position and relationship between the species of the subgenus *Schizomavella* remain unresolved, which could be caused by the existence of an elevated number of polymorphic characters. This problem has already encountered in phylogenetic analyses of bryozoan genera (see discussion)

The structure of the ovicell of *Calvetomavella* n. subgen. is similar to that in the genus *Metroperiella* Canu & Bassler, 1917, but this genus differs in having a much wider orificial sinus, in the absence of oral spines, in a more evenly perforated frontal shield, and in the consistent presence of a single, pointed, suboral avicularium. On the other hand, the number of frontal shield pores is highly variable in the different species of *Schizomavella s*.*l*., from very numerous (e.g. in *S*. *(C*.*) phterocopa* n. sp.), to extremely reduced or almost absent (e.g. in *S*. *(S*.*) fischeri* or *S*. *(C*.*) neptuni*), which makes the distinction between *Schizomavella* and *Parkermavella* somewhat difficult.

Subgenus ***Schizomavella*** Canu & Bassler, 1917

Type species: *Lepralia auriculata* Hassall, 1842, by original designation.

*Other species included in the subgenus*: *Lepralia cornuta* Heller, 1867; *Lepralia hastata* Hincks, 1862; *Lepralia linearis* Hassall, 1841; *Schizomavella gautieri* Reverter-Gil & Fernández-Pulpeiro, 1997; *Schizomavella grandiporosa* Canu & Bassler, 1925; *Schizomavella hondti* Reverter-Gil & Fernández-Pulpeiro, 1996; *Schizomavella rectangularis* n. sp.; *Schizomavella sarniensis* Hayward & Thorpe, 1995; *Schizomavella subsolana* Hayward & McKinney, 2002; *Schizomavella teresae* Reverter-Gil & Fernández-Pulpeiro, 1996; *Schizomavella triangularis* Reverter-Gil & Fernández-Pulpeiro, 1997; *Schizoporella auriculata* var. *asymetrica* Calvet, 1927; *Schizoporella auriculata* var. *hirsuta* Calvet, 1927; *Schizoporella fischeri* Jullien, 1882; *Schizoporella linearis* var. *mamillata* Hincks, 1880; *Schizoporella richardi* Calvet *in* Jullien & Calvet, 1903; *Smittina halimedae* Gautier, 1955.

*Differential diagnosis*: *Schizomavella* with few oral spines (with some exceptions), usually present only in early ontogeny, absent in ovicellate zooids. Periorbital region not raised. Avicularia monomorphic or polymorphic, single, suboral, rarely double, lateral; rostrum usually oval, rarely pointed. Ooecium marginally covered by a thick imperforate layer of secondary calcification, with a proximal area of exposed pseudoporous ectooecium; or later almost completely covered by a thick, nodular secondary calcification that does not occlude the pseudopores.

*Remarks*: The list of species that remain within the subgenus *Schizomavella* is incomplete, as the status of other species of the genus must still be revised and species correctly redescribed. Moreover, the monophyly of the subgenus *Schizomavella* is not supported by the phylogenetic analysis, and the position of some species remains unresolved (Fig 1). In the Atlantic species *S*. *richardi*, the Atlantic-Mediterranean *S*. *fischeri* (see below), and the Mediterranean *S*. *halimedae* [14], the autozooids have a relatively great number of oral spines, which is rather typical for the subgenus *Calvetomavella* n. subgen.; the first two species even have a pair of stout spines in ovicellate zooids. However, the morphology of the ovicell is similar to *S*. *auriculata*, and only a single suboral avicularium is present. Therefore, in our opinion they should remain within the subgenus *Schizomavella*.

The subgenus *Schizomavella* seems to be a group of mainly shallow water species, ranging in European waters from the N Atlantic to the NW coast of Africa and extending into the Mediterranean at least as far east as Chios. The species are also present in the Azores, Madeira and the Canaries. Records outside this area must still be confirmed.

Subgenus ***Calvetomavella*** n. subgen.

urn:lsid:zoobank.org:act:2974E67C-0D10-4DF7-AA1E-475BD502CE91

Type species: *Schizomavella (Calvetomavella) phterocopa* n. sp.

*Other species included in the subgenus*: *Lepralia discoidea* Busk, 1859; *Schizoporella neptuni* Jullien, 1882; *Schizoporella noronhai* Norman, 1909; *Schizoporella triaviculata* Calvet *in* Jullien & Calvet, 1903; *Schizoporella triaviculata* var. *paucimandibulata* d’Hondt, 1975.

*Differential diagnosis*: *Schizomavella* with a great number of long distolateral oral spines forming a semicircle, present in almost all zooids throughout ontogeny; one or two pairs visible in ovicellate zooids, or replaced by a peristome during ontogeny. Periorbital region usually imperforate and raised. Reduced secondary calcification of frontal shield in comparison with shallow-water *Schizomavella s*.*s*. Avicularia usually paired, distolateral, often enlarged and parallel to the distal zooidal margin, frequently asymmetrically developed, small if suboral; rostrum generally pointed, small avicularia occasionally oval. Exposed ectooecial area large, evenly perforated by circular or irregular shaped pseudopores, never thickly covered or immersed by secondary calcification during ontogeny; if present, the imperforate secondary calcification forms a narrow periooecial rim.

*Etymology*: Name compound of ‘Calvet’ and ‘*Schizomavella*’. This subgenus is named in honour of the French bryozoologist Louis Calvet.

*Remarks*: The erection of this subgenus is well supported by the phylogenetic analysis. The relationship between species within the clade is however not resolved by the analysis, showing a polytomy formed by four branches and only two species-pairs with relatively well-supported relationships between them (Fig 1).

Besides the species described below, *Calvetomavella* n. subgen. also includes the Madeiran species *S*. *noronhai*, which has been recently redescribed by Berning [15] and interpreted as the sibling species of *S*. *neptuni*.

**Fig 2:**
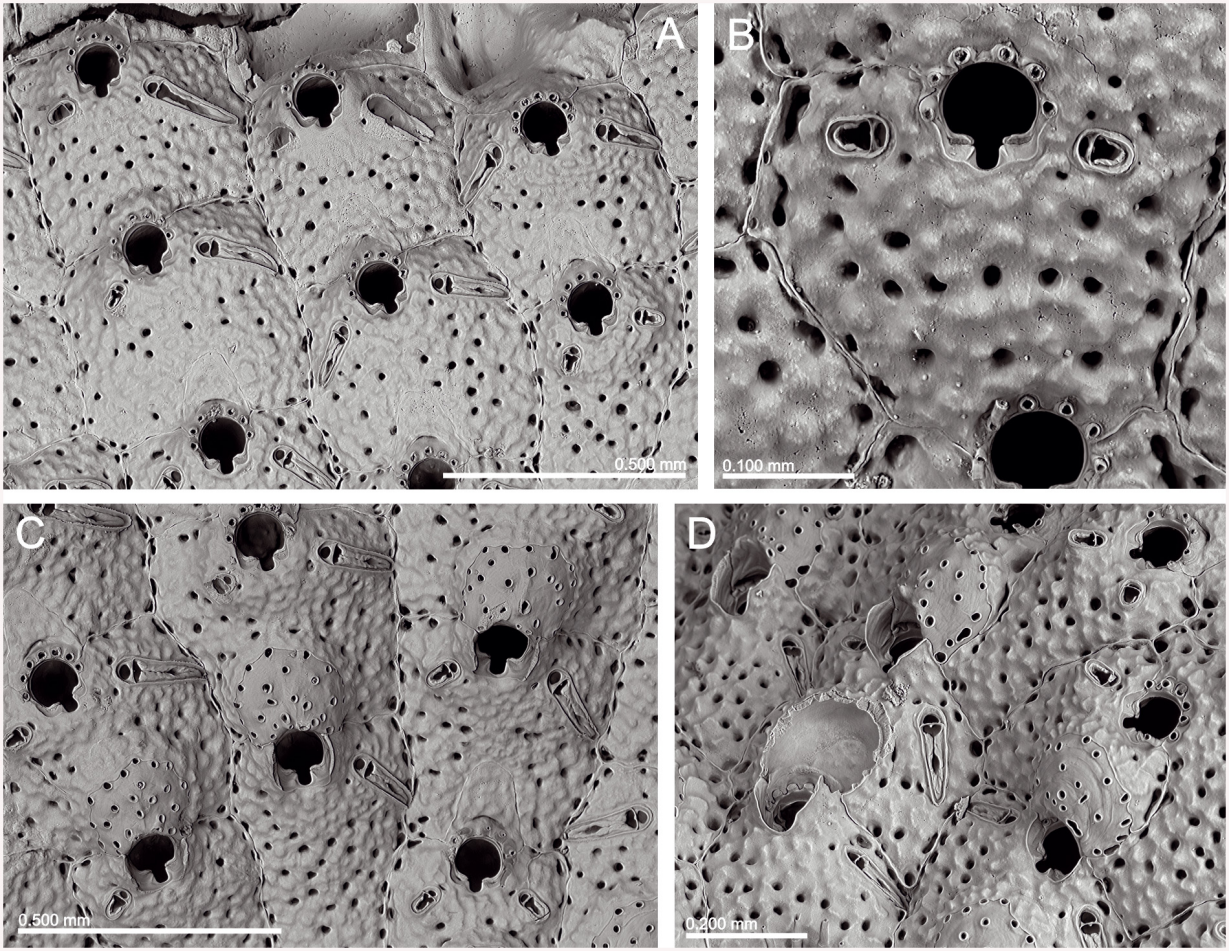
*Schizomavella (Calvetomavella) discoidea* (Busk, 1859) (NMHUK 1911.10.1088, Madeira). (A) Group of infertile autozooids; note the variable size of avicularia. (B) an autozooid with small, oval avicularia; (C) ovicellate zooids; (D) ovicellate zooids showing the development of the peristome and avicularia.

*Lepralia discoidea* was also described from Madeira. Its type material was not found at the NHMUK, but we have seen Madeiran material from the Norman Collection (NHMUK 1911.10.1.1088, Fig 2). Comparison of this material with European continental shelf records, reported in the Atlantic from the Netherlands and Great Britain to the Iberian Peninsula as well as in the W Mediterranean [14,33,34,35], strongly suggests that these records actually correspond to at least one new undescribed species, but a thorough revision of the material will be necessary. In any case, the morphology of the ovicell of *S*. *discoidea s*.*l*., the pair of distolateral avicularia, and the great number of oral spines allow transferring this species complex to *Calvetomavella* n. subgen.

The species assigned to *Calvetomavella* n. subgen. mainly occur in deep water in the NE Atlantic (Macaronesia). Two species are endemic to the Azores, one to the Great Meteor Bank, and another one to Madeira. *Schizomavella discoidea* is known with certainty only from Madeira. *Schizomavella neptuni* is the only species of the subgenus absent from the Macaronesian region, being present from the NW Bay of Biscay to the western Mediterranean. As stated above, another new species, closely related to *S*. *discoidea*, may also be present in the European continental shelf.

### Description of species

***Schizomavella (Schizomavella) fischeri*** (Jullien, 1882)

(Figs 3–5, Table 1)

**Fig 3:**
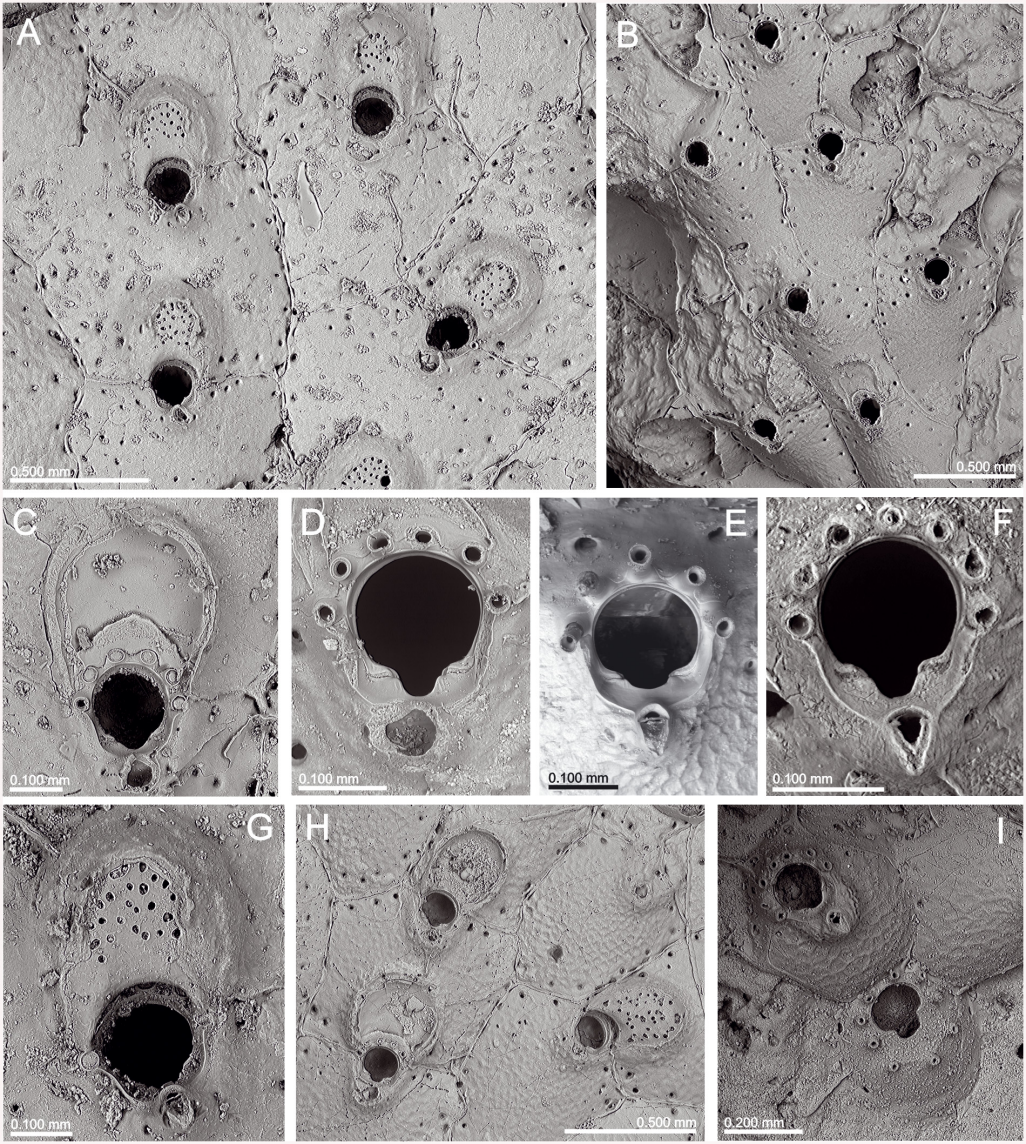
*Schizomavella (Schizomavella) fischeri* (Jullien, 1882). (A) Ovicellate zooids (MNHN 2966, lectotype); (B) a young colony (MNHN 3783, paralectotype); (C-F) several primary orifices (C, MNHN 2966, lectotype; D, MNHN 3783, paralectotype; E, *BANGAL* 2011 V01; F, MB37-000038); (G) detail of the ovicell and the suboral avicularium (MNHN 2966, holotype); (H) ovicellate zooids (MNHN IB-2013-567); I. ancestrula and first budded zooid (MNHN IB-2013-568).

**Fig 4:**
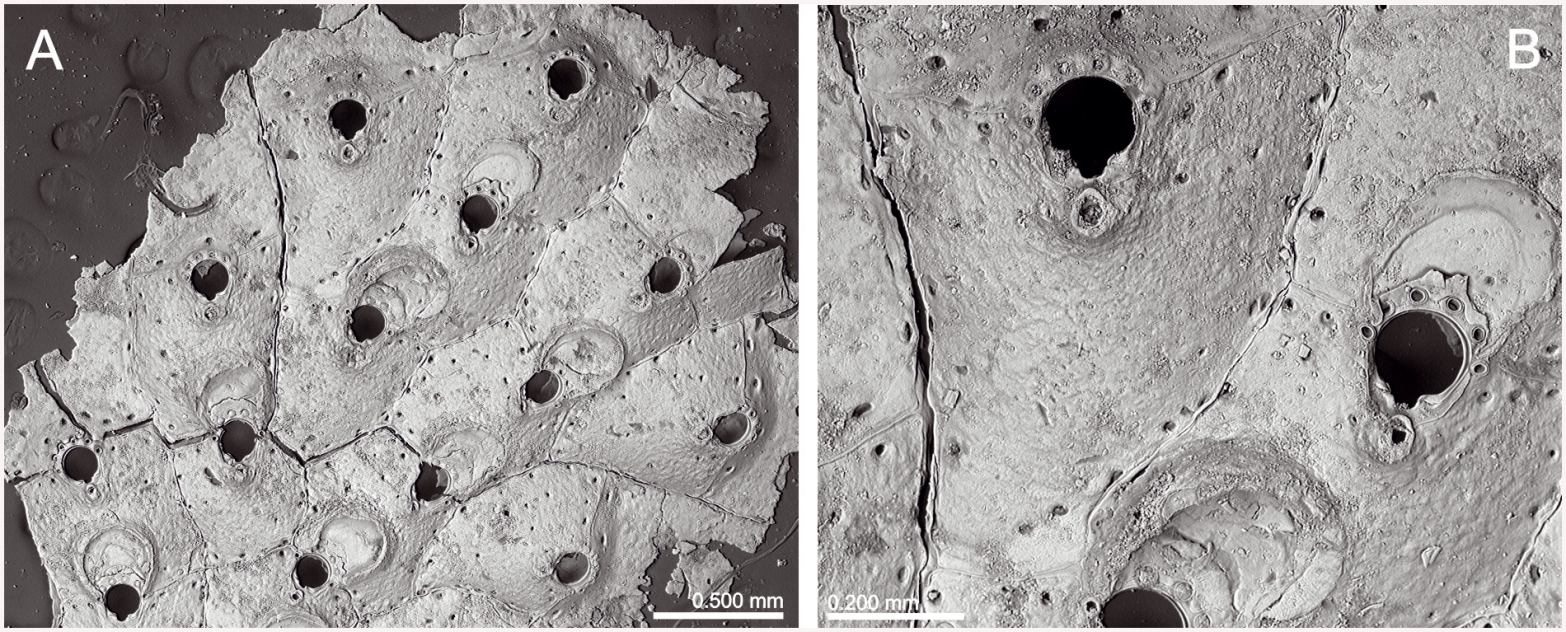
*Schizoporella obsoleta* Jullien, 1882 (MNHN 1024, holotype). (A) Part of the colony; B. infertile zooids.

**Fig 5:**
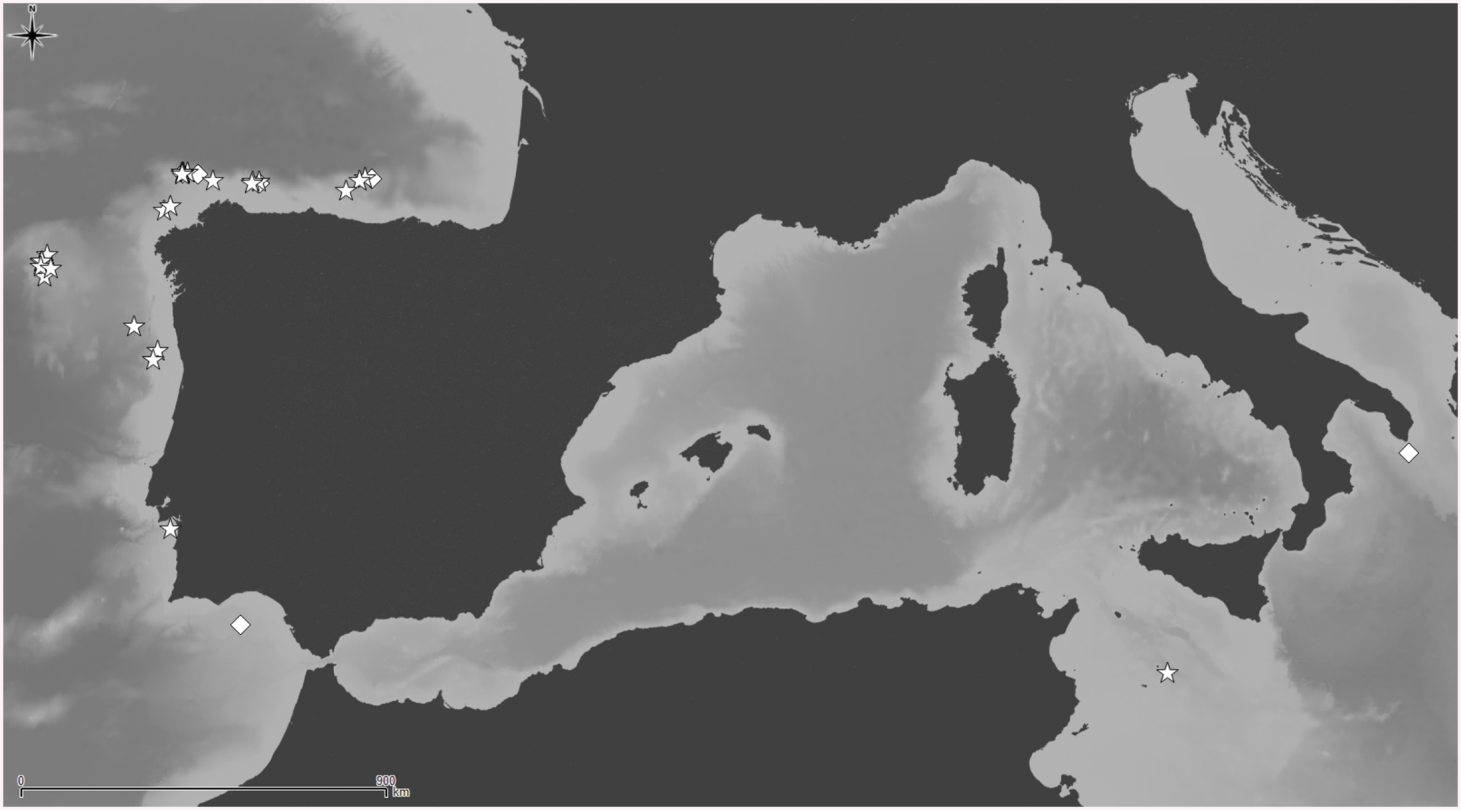
Distributional records of *Schizomavella (Schizomavella) fischeri* (Jullien, 1882) gathered from the literature and material examined. (Stars—material examined, diamonds—unconfirmed record taken from literature). Note that some symbols represent more than one record as the resolution of the map is insufficient to depict all records.

**Table 1:** Measurements (in mm) of *Schizomavella (Schizomavella) fischeri* (Lectotype and *BANGAL* 2011 DR08).

	Mean	SD	Minimum	Maximum	N
Autozooid length	0.733	0.0826	0.574	0.892	24
Autozooid width	0.677	0.1461	0.500	0.930	24
Orifice length	0.134	0.0091	0.114	0.152	26
Orifice width	0.142	0.0079	0.126	0.161	26
Ovicell length	0.289	0.0272	0.240	0.325	16
Ovicell width	0.315	0.0201	0.289	0.349	16
Avicularium length	0.066	0.0105	0.045	0.080	16
Avicularium width	0.041	0.0042	0.031	0.048	16
Pore density	2	1	0	4	16

SD, Standard deviation; N, number of measurements.

*Schizoporella fischeri* Jullien, 1882: 15, pl. 14, figs. 32, 33 [1]; Jullien, 1883: 511, pl. 14, figs. 32, 33 [2].

*Schizoporella fischeri* nov. sp.: Calvet, 1907: 422 [6].

*Schizomavella fischeri* (Jullien): d’Hondt, 1973: 371 [27]; d’Hondt, 1974: 40 [36]; Harmelin & d’Hondt, 1992: 44, pl. 5, E-F [30]; Reverter-Gil & Fernández-Pulpeiro, 2001: 119 [34]; Reverter-Gil et al., 2014: 25 [37].

*Schizoporella obsoleta* Jullien, 1882: 16, pl. 15, fig. 35 [1]; Jullien, 1883: 512, pl. 15, fig. 35 [2].

*Schizoporella obsoleta* nov. sp.: Calvet, 1907: 422 [6].

*Schizomavella obsoleta* (Jullien): Reverter-Gil & Fernández-Pulpeiro, 2001: 120 [34].

Not *Schizoporella obsoleta* (Jullien): d’Hondt, 1975: 575 (part or whole) [18].

*Type material*: Lectotype of *Schizoporella fischeri* (chosen here): MNHN 2966, *Travailleur* Stn 2 (1^er^ sér.), 41°43'00''N 09°19'26''W, off N Portugal, 14/06/1881, 1068 m, Jullien Coll.

Paralectotype of *Schizoporella fischeri* (chosen here): MNHN 3783: same data as lectotype of *S*. *fischeri*, Calvet Coll.

Holotype of *Schizoporella obsoleta* (by monotypy): MNHN 1024, *Travailleur* Stn 40, 44°05'00''N 07°14'46''W, off NW Iberian Peninsula, 15/08/1881, 392 m, Jullien Coll.

*Other material examined*: See S2 Text.

*Description*: Colony encrusting, unilaminar, multiserial, forming irregular crusts.

Autozooids in regular linear series, sometimes alternating; rectangular to polygonal, large, broad, usually widest distally; zooecia separated by sutures on slightly raised, thin rims. Distolateral vertical walls with few small round communication pores.

Frontal shield rather flat or slightly convex, somewhat rising distally towards orifice; smooth or slightly granular and rugose. A row (rarely two) of small scattered marginal pores plus some pores in the disto-lateral corners; most of the central frontal surface imperforate, or rarely only with few scattered pores.

Primary orifice orbicular, slightly wider than long, situated slightly elevated.

Distal half of orifice projecting beyond distolateral zooidal margins, surrounded by 5–7 (sometimes 8) stout, jointed spines, often broken; the proximal pair visible in ovicellate zooids. Open and broadly U-shaped sinus occupying half to one third of the proximal border; condyles relatively thin and inconspicuous, smooth, sloping towards the edges of the sinus. Proximal half of the primary orifice surrounded by an elevated rim of smooth gymnocystal calcification, somewhat quadrangular in outline.

Avicularia adventitious, single, situated on a raised cystid the distal part of which being incorporated into the central gymnocystal suboral area; rostrum elongate triangular, curved downward in distal half, directing proximally, but frequently broken in the material examined; proximal uncalcified area triangular, bounded by a well-developed distal shelf, distal area semicircular, crossbar complete without columella.

Ovicell hyperstomial, globular but distinctly flattened frontally, about as wide as long, with the secondary calcification of the distal zooid covering the entire lateral ooecium, forming a raised serrated rim around the central, flattened exposed ectooecium which is perforated by numerous pseudopores of variable shape and comparatively small size. Ovicell opening compressed, not closed by the operculum.

Ancestrula observed once, oval, longer than wide (0.38 x 0.33 mm); gymnocyst well developed proximally, narrowing and steepening distolaterally. Opesia mushroom-shaped, with a large U-shaped sinus formed by the cryptocyst, surrounded by 9 mural spines, with the two distal pairs positioned slightly closer to each other, a third pair positioned level with the sinus of the opesia, and three proximal spines. First autozooid budded distally, plus two zooids budded distolaterally. One round communication pore per neighbouring zooid.

*Remarks*: *Schizoporella fischeri* was originally described from material collected at 1068 m depth off N Portugal. The original specimens are stored at the MNHN and are here designated as lectotype and paralectotype of the species. This record of the species was eventually compiled by Calvet [6]. The species was not reported again until it was mentioned by d’Hondt [27,36], collected in the Bay of Biscay and NW of the Iberian Peninsula, between 300 and 1070 m depth. This author also first transferred the species to the genus *Schizomavella*. The species was reported again by Harmelin & d’Hondt [30] from one station in the Gulf of Cadiz, at 485 m depth. Later, Reverter-Gil & Fernández-Pulpeiro [34] compiled records of the species from NW Spain, and Reverter-Gil et al. [37] from Portugal, in both cases adding some new data. In the Mediterranean the species has been rarely reported, such as from the deep waters south of Italy [38 according to 30,39,40,41]. However, as SEM images of Mediterranean colonies are not provided in any of these publications, it is impossible to judge whether these records are indeed conspecific.

*Schizoporella obsoleta* was originally described from a single dead colony, collected at 392 m depth NW of the Iberian Peninsula [N.B.: according to Calvet (1907: 422) [6], Jullien [1] erroneously indicated 896 m depth for this station]. The sample MNHN 1024, which coincides with the specimen figured by Jullien (1882, pl. 15, fig. 35) [1], is therefore the holotype of *S*. *obsoleta* by monotypy. The species was mentioned again by Calvet [6] from this and other material collected at the same locality (MNHN 2348); however, this sample is not part of the type series, despite the indication by [42]. Later, d’Hondt [18] reported this species from several localities in the Azores, but the samples conserved do not correspond to *S*. *obsoleta*; sample MNHN 7534 is too poorly preserved to be properly identified, and MNHN 7547 is *Stephanotheca fayalensis* n. comb. (see below). The species does not seem to have been reported from elsewhere until the compilation of records from off Galicia (NW Spain) made by Reverter-Gil & Fernández-Pulpeiro [34], who transferred the species to the genus *Schizomavella*.

SEM imaging of the holotype of *S*. *obsoleta* shows that it is morphologically identical with the original material of *S*. *fischeri*; moreover, all the material comes from the same area (NW Iberian Peninsula). We therefore consider the two species as synonymous. Taking into account that *S*. *fischeri* was described first in the original paper, and that this name had been more widely used in subsequent literature we here decide to give precedence to the name *Schizoporella fischeri* according to Article 24 of the ICZN Code [43]. As stated above, we consider that this species must remain within *Schizomavella s*.*s*., although it has a relatively great number of oral spines, and a pair of stout spines in ovicellate zooids.

The spines of *S*. *(S*.*) fischeri* are frequently disarticulated and not preserved in dried material, but they were described by d’Hondt [36] as pointed, slender, and nearly as long as a zooid. Only the most proximal pair of spines is visible in ovicellate zooids, but the bases of the other spines are also present underneath the ovicell along the distal orifice margin.

It must also be stated that in the types and other old material the suboral avicularium is frequently broken, or at least eroded, giving a false appearance of being oval, but in the original descriptions, as well as in better preserved material, the avicularium is typically pointed.

*Schizomavella (S*.*) fischeri* seems to show a wide range of intercolonial variability regarding some of its characters, such as width of sinus, number of oral spines and number of perforations in the frontal shield. In the lectotype, as well as in material from the Galicia Bank, the sinus is wide and shallow, occupying about half of the proximal border of the primary orifice. In other material from NW Iberian Peninsula the sinus occupies one third of total proximal width, while in material from the Gulf of Cadiz and the Mediterranean Sea the sinus is indeed narrower, and is flanked by two sharp, upturned shoulders. Colonies from NW Iberian Peninsula, including the lectotype, usually have 6 oral spines, rarely 5, but colonies from *Poseidon* Stn 2 (MB37-000038) present 7 or even 8 oral spines. Material from the Galicia Bank has 5 spines, rarely 6, while in the Gulf of Cadiz Harmelin & d’Hondt [30] reported 5 to 7 spines. Other variations affect the extent of the exposed area of ectooecium in the ovicell, the number of ectooecial pseudopores, or the development of the raised serrated rim around the central, flattened, ectooecial area; these characters, however, are also subject to variation within a colony.

We were not able to establish if these differences simply represent ecological and/or geographical variations of a single species, or if they are of taxonomic importance; this needs to be assessed using combined morphological and genetic analyses. Therefore, we provisionally recognise here a single, variable species in favour of a complex of closely related species. Accordingly, we consider *S*. *(S*.*) fischeri* to be present around the Atlantic coast of the Iberian Peninsula, from the North to the Gulf of Cadiz, between 250 and 1070 m depth, but frequently deeper than 400 m depth. In the Mediterranean it seems to be a very rare species, and is also reported from bathyal environments: in the strait between Sicily and Malta at 320–600 m depth [38 as *Schizomavella* sp.; present paper] and recently from the Apulian plateau (Italy), associated with white corals at 513–528 m depth [39,40,41].

***Schizomavella (Schizomavella) richardi*** (Calvet *in* Jullien & Calvet, 1903)

(Figs 6–8, Table 2)

**Fig 6:**
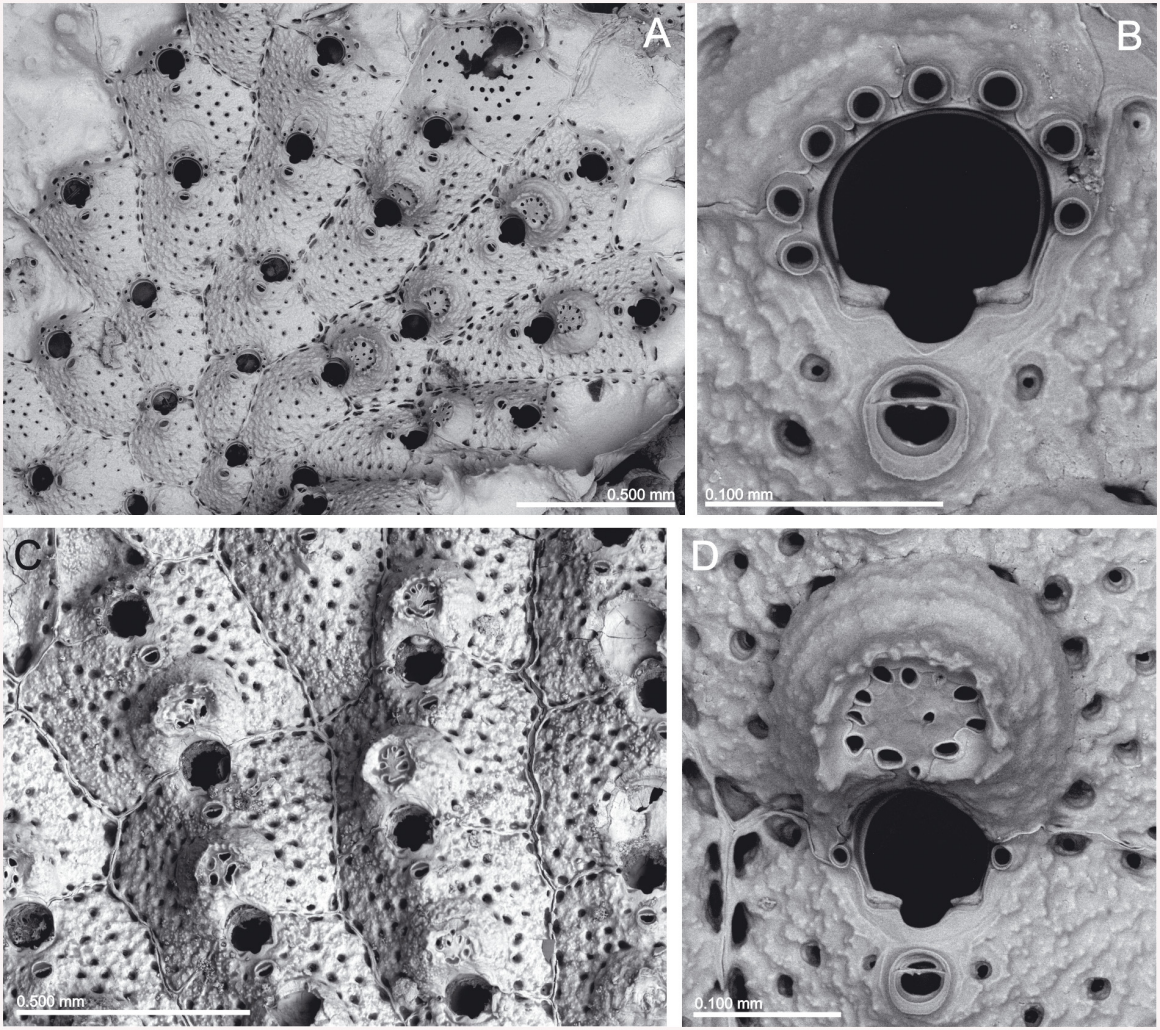
*Schizomavella (Schizomavella) richardi* (Calvet *in* Jullien & Calvet, 1903). (A) Part of a colony (MNHN 2436); (B) same, primary orifice; (C) ovicellate zooids (MOM INV-22505, lectotype); (D). closer view of the ovicell (MNHN 2436).

**Fig 7:**
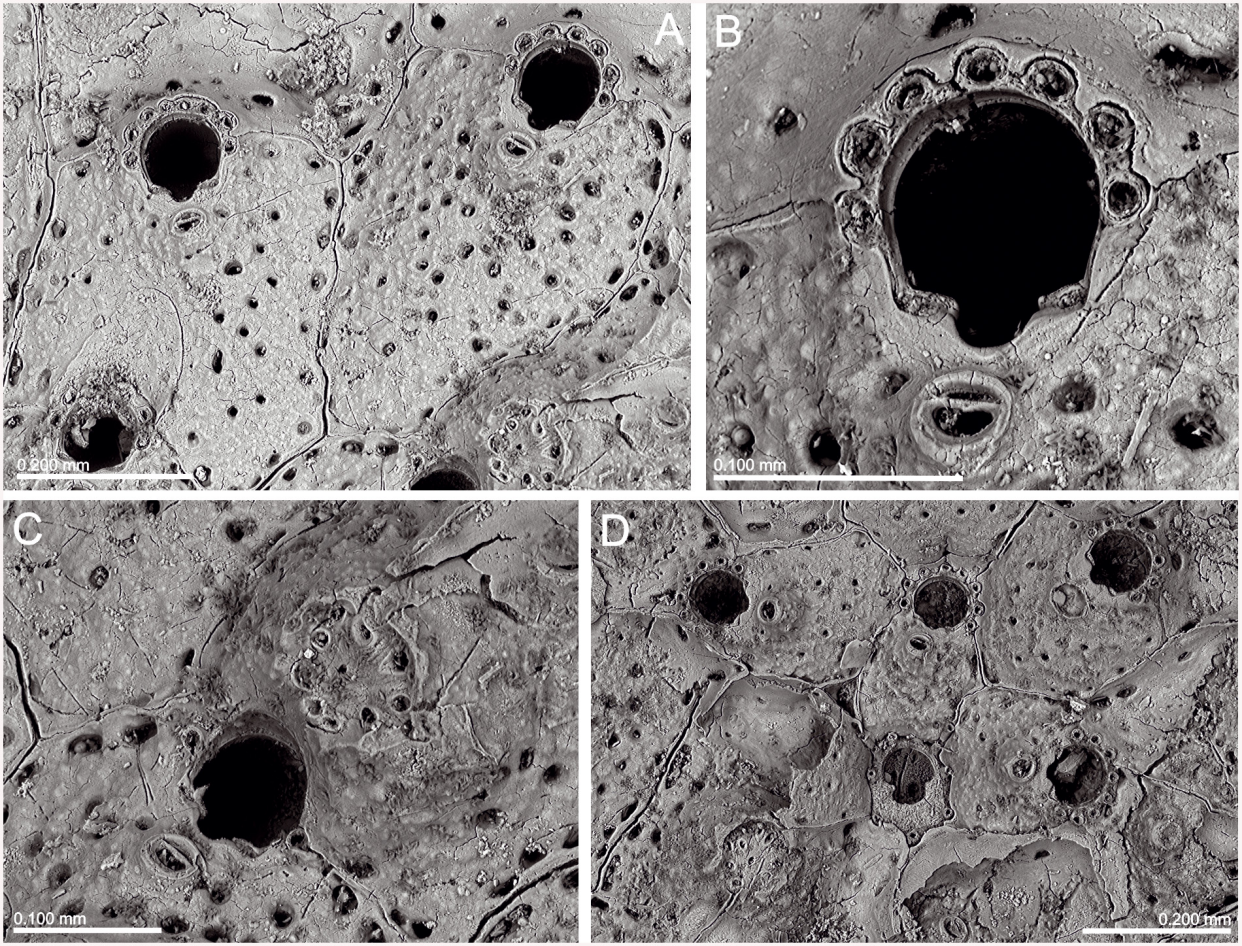
*Schizoporella glebula* Jullien & Calvet, 1903 (MOM INV-22501, lectotype). (A) Two infertile zooids; (B) primary orifice; (C) ovicell; (D) ancestrula and first budded zooids.

**Fig 8:**
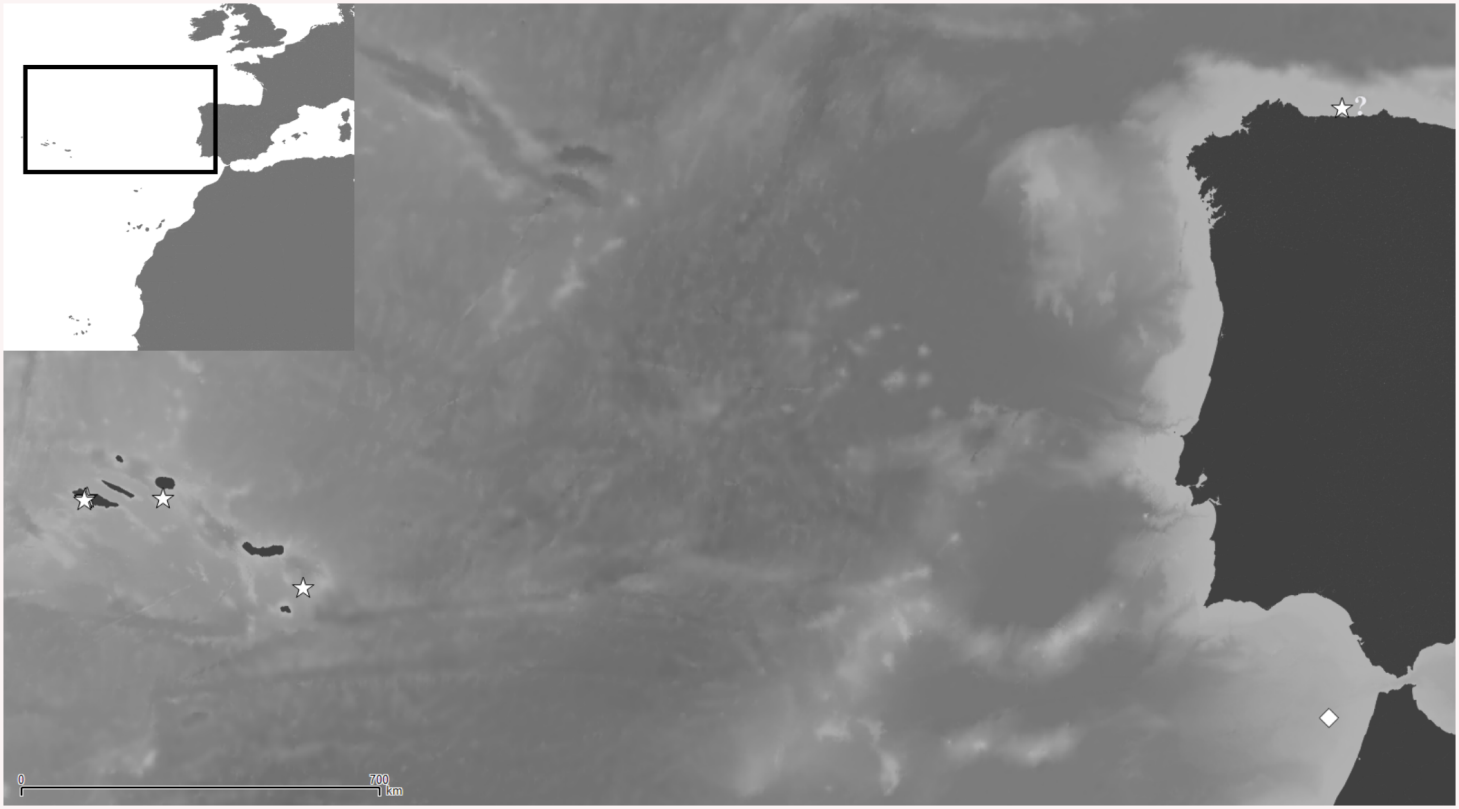
Distributional records of *Schizomavella (Schizomavella) richardi* (Calvet *in* Jullien & Calvet, 1903) and *Schizomavella (Schizomavella) rectangularis* n. sp. gathered from material examined. (Stars–*S*. *richardi*, diamond–*S*. *rectangularis*). Note that some symbols represent more than one record as the resolution of the map is insufficient to depict all records.

**Table 2:** Measurements (in mm) of *Schizomavella (Schizomavella) richardi* n. comb. (MOM INV-22505; MNHN 2436, 4038).

	Mean	SD	Minimum	Maximum	N
Autozooid length	0.417	0.035	0.366	0.488	20
Autozooid width	0.416	0.060	0.315	0.572	20
Orifice length	0.099	0.004	0.090	0.105	20
Orifice width	0.093	0.004	0.084	0.100	20
Ovicell length	0.192	0.013	0.170	0.216	15
Ovicell width	0.216	0.014	0.198	0.244	15
Avicularium length	0.043	0.003	0.038	0.051	20
Avicularium width	0.038	0.004	0.029	0.045	20
Pore density	13	2	9	17	17

SD, Standard deviation; N, number of measurements.

*Schizoporella glebula* Jullien & Calvet, 1903: 81, pl. 10, fig. 5 [3].

*Schizoporella richardi* Calvet *in* Jullien & Calvet, 1903: 140 (not p. 169), pl. 16, fig. 6a-b [3].

*Schizoporella richardi* Calvet: Calvet, 1907: 422 [6]; d’Hondt, 1975: 562 [18].

*Schizomavella glebula* (Jullien & Calvet): Reverter-Gil & Fernández-Pulpeiro, 2001: 121 [34].

Not *Schizoporella richardi* nov. sp.: Jullien & Calvet, 1903: 169 [3] [= *Schizomavella auriculata* (Hassall, 1842)].

*Schizomavella richardi* (Calvet): Wisshak et al., 2015: 95 [22].

*Type material*: Lectotype of *Schizoporella glebula* (selected here): MOM INV-22501, *Hirondelle* Stn 58, 43°40'00''N 06°34'46''W, off N Iberian Peninsula, 07/08/1886, 134 m, Calvet Coll.

Lectotype of *Schizoporella richardi* (selected here): MOM INV-22505, *Hirondelle* Stn 226, 38°31'19''N 28°34'31''W, Pico-Faial Channel (Azores), 14/08/1888, 130 m, a colony on *Reteporella* sp., Calvet Coll.

Paralectotypes of *Schizoporella richardi* (selected here): MOM INV-22615, -22616, -22617, -22618, -22619 and -22621, colonies and fragments on shells, same data as lectotype of *S*. *richardi*, Calvet Coll.

*Other material examined*: See S2 Text.

*Description*: Colony encrusting, unilaminar, multiserial, forming small subcircular crusts.

Autozooids in regular radial series, sometimes alternating; rectangular or quadrangular, sometimes broader than long; zooecia separated by sutures on thin rims. Distolateral vertical walls with several round communication pores.

Frontal shield slightly convex, granular, evenly perforated by 16 to 30 round pores, plus a row of elongate, marginal areolae.

Primary orifice suborbicular to horseshoe-shaped, only slightly longer than wide. Distal half or more of the orifice margin projecting beyond the distal zooecial margin, with 7–8 (sometimes 6) stout, jointed spines, often disarticulated; only the proximal pair exposed in ovicellate zooids. Open U-shaped sinus occupying half to one third of the proximal border, proximolateral margins usually slightly oblique, rising towards the shoulders of the sinus; condyles small, rectangular, smooth, slightly sloping towards the edges of the sinus, with the pointed tips facing each other.

Avicularia adventitious, single, situated on a raised cystid. Distance to the sinus may vary in different colonies, from almost touching it to separated by as much as the length of the avicularium. Rostrum directed and raised towards the proximal zooid margin, semicircular; distal uncalcified area semicircular, bounded by a well-developed distal shelf, proximal foramen elliptical, crossbar complete with a very small columella.

Ovicell hyperstomial, globular but distinctly flattened frontally. Secondary calcification of the distal zooid covering most of the ooecium, forming a thick raised serrated rim around the exposed, circular to D-shaped central ectooecium which is perforated by several pseudopores of variable shape and size. Ovicell opening compressed, not closed by the operculum.

Ancestrula oval, small, with 6 distal spines plus 3 widely spaced proximal ones surrounding a mushroom-shaped opesia with a large U-shaped sinus that is constricted by a well-developed proximolateral cryptocyst. First autozooid budded distally, two second-generation zooids budded laterally, oval in outline. Ancestrula overgrown by increasingly larger autozooids relatively early during astogeny.

*Remarks*: *Schizoporella glebula* was described by Jullien & Calvet [3] based on two colonies collected at two stations of the *Hirondelle* cruise in the Bay of Biscay, at 134 m and 155 m depth. Only the colony of Stn 58 (MOM INV-22501) is preserved, and is here designated as lectotype of the species. The figure in Jullien & Calvet (1903, pl. 10, fig. 5) [3] does not seem to correspond to this sample, so it perhaps represents the colony of Stn 46, which seems to be lost. *Schizoporella glebula* was not subsequently reported until its original record was compiled by Reverter-Gil & Fernández-Pulpeiro [34], but without revision of the original material. These authors transferred the species to the genus *Schizomavella*.

*Schizoporella richardi* was first described by Calvet [3] based on several colonies collected during the *Hirondelle* cruise in the Pico-Faial channel (Azores), at 130 m depth. The original specimens are stored at the MOM and are here designated as lectotype and paralectotypes of the species. There are two other specimens at the MOM (INV-22504, 22620) from *Hirondelle* Stn 58 (NW Iberian Peninsula), which were identified by Calvet as *S*. *richardi*. This material is actually *Schizomavella auriculata* (Hassall, 1842). However, these specimens were not cited in the original description of *S*. *richardi* but only in the list of localities (Jullien & Calvet, 1903: 169) [3] and must not be considered as part of the type series.

*Schizoporella richardi* was subsequently reported by Calvet [6] from its original area, at 80–115 m depth, and later by d’Hondt [18] between São Miguel and Santa Maria Islands (Azores), at 155–290 m depth [N.B.: the coordinates of the latter station (Stn 208) were erroneously reported by d'Hondt [18] as 31°16'N; the correct latitude must be 37°16'N, as Stns 207 and 209 are both at about 37°N]. Recently, Wisshak et al. (2015) [22] have recorded the species on settlement panels at 150 m depth in the Pico-Faial Channel.

The type material of *S*. *glebula* is morphologically indistinguishable from that of *S*. *richardi*. Therefore, the species must be considered as synonymous, despite the geographical distance between both type localities (NW Iberian Peninsula vs. the Azores). Their morphological congruence is astonishing and genetic analyses will be needed to clarify their true relationship and natural history of the species. As it has never been reported from shallow water, an introduction to the Azores on natural rafts or on artificial debris or ship's hulls is unlikely.

Both species were published in the same paper, whereas *S*. *glebula* was described before (by Jullien & Calvet, p. 81) and *S*. *richardi* later (by Calvet, p. 140), so in principle the first name would have precedence. However, *S*. *glebula* was only cited in the original paper (except for the compilation by [34]), and only a single, small colony is preserved. On the other hand, there are six original slides of *S*. *richardi*, and the species was reported in at least two subsequent papers, the material of which is also still existent in part. Furthermore, new comparative material has recently been collected near the type locality. Therefore, according to ICZN Article 24 [43], we decide to give precedence to the name *Schizoporella richardi*.

There are some differences between the present description and the original ones made by Jullien & Calvet [3]. In the description of *S*. *glebula* Jullien & Calvet stated that the suboral avicularium is frequently missing, but in the lectotype most of the zooids have an avicularium. More striking is the assertion made by Calvet in the description of *S*. *richardi*, when he stated that the oral spines are completely absent from ovicellate zooids; this is also visible in the original figure (Jullien & Calvet, 1903, pl. 16, fig. 6a) [3]. However, the most proximal pair of spines is present in almost all ovicellate zooids, and even the bases of the remaining spines are visible underneath the ovicell along the distal orifice margin. Only in some older zooids the bases of spines become obscured by secondary calcification.

This species is closely related to *S*. *fischeri*, both having a high number of oral spines, of which only two are visible in ovicellate zooids; an open U-shaped sinus occupying half to one third of the proximal border of the orifice; a centrally exposed ectooecium surrounded by a raised rim of secondary calcification; and both have a single suboral avicularium. However, differences are evident: among other characters *S*. *richardi* has 7–8 oral spines, pointed condyles, 15–30 frontal pores (with a mean pore density of 13/0.04mm^2^), an oval avicularium, the area of exposed ectooecium in the ovicell is smaller, circular or D-shaped, and the rim of secondary calcification surrounding it is thick and much more elevated. *Schizomavella fischeri* has 5–6 spines, smaller condyles sloping towards the sinus, 6–12 smaller frontal pores (with a mean pore density of 2/0.04mm^2^), a pointed avicularium, and the rim of secondary calcification surrounding the central area of the ovicell is less developed. Finally, *S*. *fischeri* occurs from 250 to 1070 m, while *S*. *richardi* is distributed between 76 and 290 m depth.

As for the previous species, characters of *S*. *richardi* allow placing it in the subgenus *Schizomavella* (but see comments above).

*Schizomavella (S*.*) richardi* is known from several localities in the Azores between 76 and 290 m depth, and from the Bay of Biscay (as *S*. *glebula*) between 134 m and 155 m depth.

**Fig 9:**
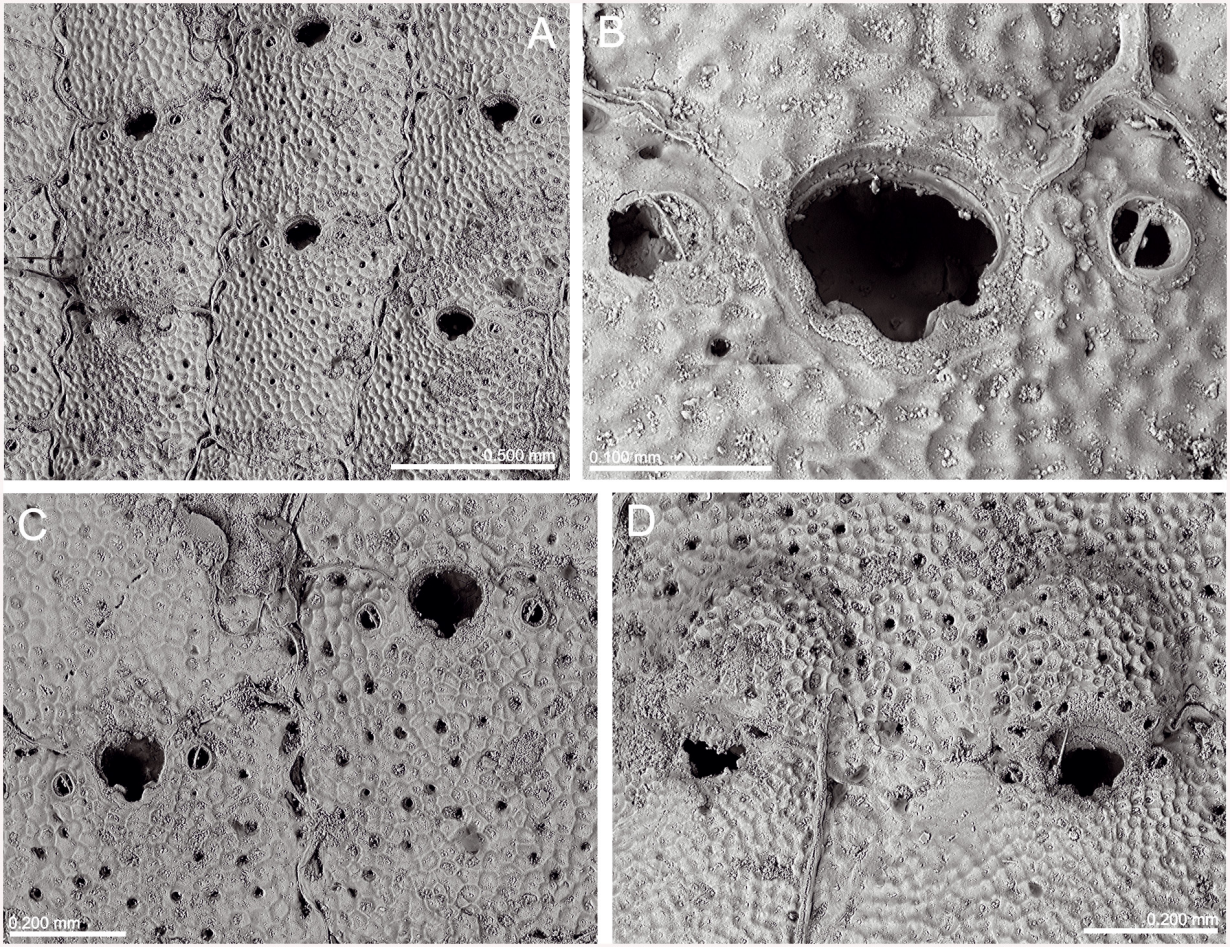
*Schizomavella (Schizomavella) rectangularis* n. sp. (MNHN 1023, holotype). (A) Group of zooids, one ovicellate, with the characteristic undulating rims; (B) primary orifices and avicularia; (C) two infertile zooids; (D) two ovicellate zooids.

***Schizomavella (Schizomavella) rectangularis* n. sp.** *urn*:*lsid*:*zoobank*.*org*:*act*:*8699E5F5-87B3-4608-86DC-8AC9B3890FD4*

(Figs 8 and 9, Table 3)

**Table 3:** Measurements (in mm) of *Schizomavella (Schizomavella) rectangularis* n. sp. (MNHN 1023: holotype).

	Mean	SD	Minimum	Maximum	N
Autozooid length	0.716	0.1060	0.589	0.985	13
Autozooid width	0.542	0.0523	0.449	0.639	13
Orifice length	0.093	0.0040	0.087	0.098	5
Orifice width	0.118	0.0038	0.112	0.122	5
Ovicell length	0.268	0.0087	0.262	0.278	3
Ovicell width	0.307	0.0137	0.298	0.323	3
Avicularium length	0.044	0.0027	0.039	0.048	9
Avicularium width	0.035	0.0044	0.030	0.042	9
Pore density	8	2	5	11	20

SD, Standard deviation; N, number of measurements.

?*Schizoporella neptuni* Jullien: Calvet, 1907: 421 (in part) [6].

*Type material*: Holotype: MNHN 1023: *Talisman* 1883, Stn 10, 35°26'00''N 06°48'46''W, near Cape Spartel (Strait of Gibraltar), 717 m, 10/06/1883, one colony on a shell fragment [originally labelled as *Schizoporella neptuni*], Calvet Coll.

*Etymology*: Alluding to the rectangular shape of the autozooids.

*Description*: Colony small, encrusting, unilaminar, multiserial.

Autozooids in regular linear series, sometimes alternating; subrectangular, broad, large, separated by sutures on raised, distinctly undulating rims. Distolateral vertical walls with several round communication pores.

Frontal shield very slightly convex, somewhat rising towards the orifice, surface entirely covered by polygonal pits separated by raised ridges, usually perforated by 20–30 small rounded pores plus a row of elongated marginal pores.

Primary orifice small compared to its own zooid-size, wider than long, horseshoe shaped. Sinus forming a widening U, occupying one-third of the proximal border and flanked by rounded shoulders; condyles conspicuous, short but broad and thick, extending beyond the sinus, and separated from the orificial rim by a lateral notch. Four to five spines present only in some zooids at the colony periphery.

Avicularia small, paired, situated on each side of the primary orifice, directed proximolaterally to laterally; rostrum semicircular; complete crossbar with a small columella.

Ovicell hyperstomial, globular, ooecium completely covered by secondary calcification with the same surface structure as frontal shields, perforated by several rounded pores.

An ancestrula was not observed.

*Remarks*: Calvet [6] reported four colonies of *S*. *neptuni* encrusting *Lophelia* collected in the *Talisman* Stn 10, near Cape Spartel, at 717 m depth. Only two of these samples (MNHN 1022; MNHN 4097) really correspond to this species (see below). Sample MNHN 2439 is an undetermined species of *Escharella*, while sample NHMUK 1899.7.1.2340 is *S*. *linearis*. The colony in the sample MNHN 1023, here designated as the holotype of *S*. *rectangularis* n. sp., is labelled as *S*. *neptuni* and is from the same station, but it encrusts a shell fragment, so it is unclear if the specimen was originally reported by Calvet [6].

Characters of the new species here described may fit the diagnosis of the subgenus *Schizomavella*. However, all the species of this subgenus (with the sole exception of *Schizomavella linearis*) have a single, suboral avicularium. Furthermore, as stated above, in the majority of species only the peripheral ooecium is covered by an imperforate layer of secondary calcification, exposing a central or proximal area of pseudoporous ectooecium varying in shape and size, while in *S*. *rectangularis* n. sp. the secondary calcification covers the entire ooecium. The pseudopores that are present in the ectooecium are not covered by secondary calcification, resulting in a porous ooecium with a similar structure as the autozooidal frontal shield.

*Schizomavella rectangularis* n. sp. differs from other species of *Schizomavella* in the Atlantic-Mediterranean region by the large, rectangular zooids separated by raised, undulating rims, the horseshoe shaped primary orifice, the presence of two small, oval distolateral avicularia at both sides of the orifice, and by an ooecium that is completely covered by perforate secondary calcification.

**Fig 10:**
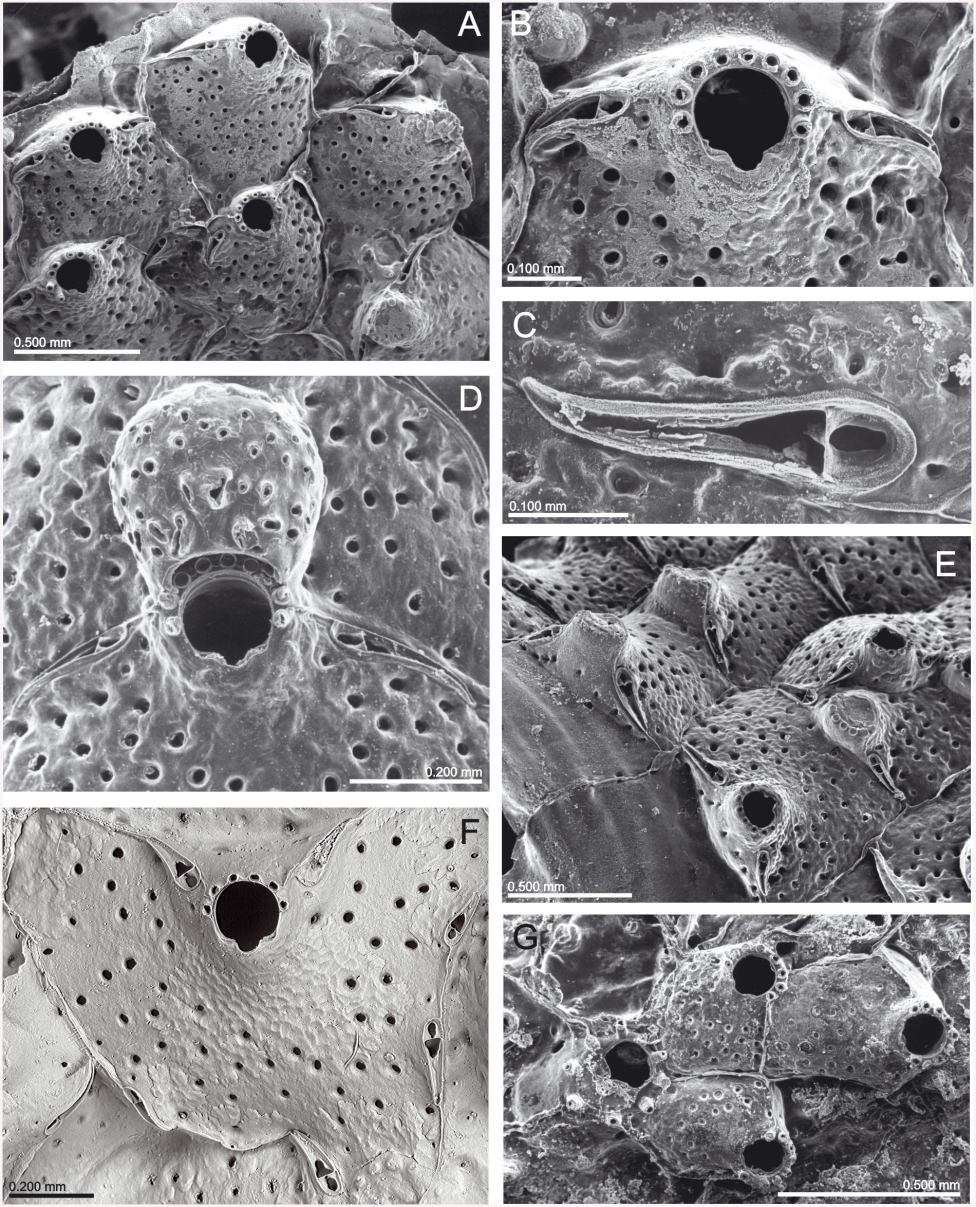
*Schizomavella (Calvetomavella) phterocopa* n. sp. (A) Group of newly budded zooids (SMF 30.051, paratype); (B) primary orifice; note the condyles with broad blunt denticles (OLL 2015/1, holotype); (C) zooids of the growing edge; note the raised periorbital region (OLL 2015/6); (D) an enlarged distal avicularium (OLL 2015/3, paratype); (E) closer view of the ovicell (OLL 2015/1, holotype); (F) a zooid with additional triangular avicularia along lateral sutures (MNHN IB-2013-573); (G) ancestrula and first budded zooids (OLL 2015/7); note that the cryptocystal sinus and proximal part of the denticles are occluded by dirt.

**Fig 11:**
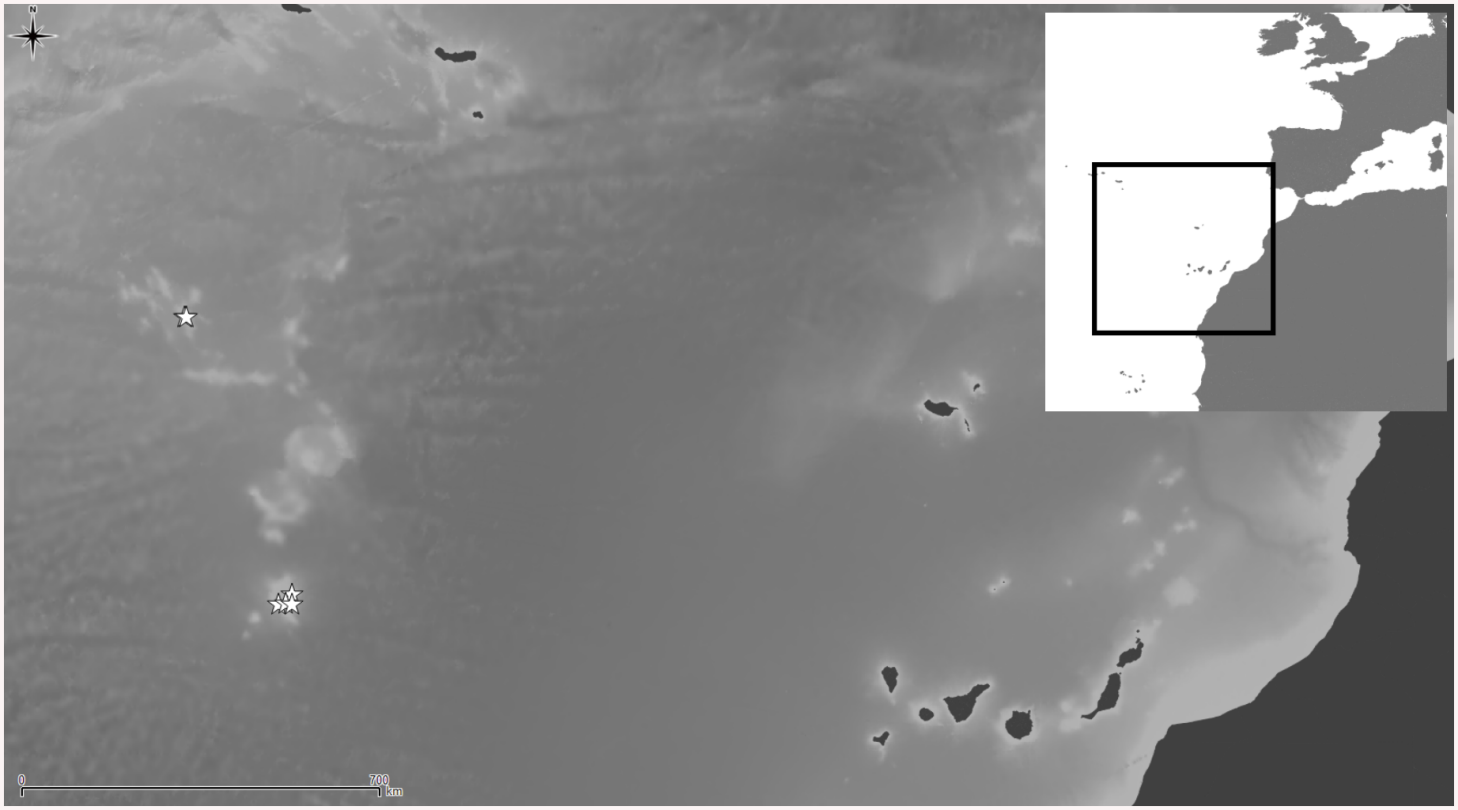
Distributional records of *Schizomavella (Calvetomavella) phterocopa* n. sp. gathered from material examined. Note that some symbols represent more than one record as the resolution of the map is insufficient to depict all records.

***Schizomavella (Calvetomavella) phterocopa* n. sp.** *urn*:*lsid*:*zoobank*.*org*:*act*:*DC86F84A-7459-41EE-82FE-7D4267ADFC19*

(Figs 10 and 11, Table 4)

**Table 4:** Measurements (in mm) of *Schizomavella (Calvetomavella) phterocopa* n. sp. (NHMUK 2015.3.4.6; OLL 2015/1, 2015/2, 2015/3, 2015/6; 2015/8; SMF 30.051).

	Mean	SD	Minimum	Maximum	N
Autozooid length	0.714	0.127	0.507	1.013	20
Autozooid width	0.659	0.124	0.452	1.029	20
Orifice length	0.149	0.009	0.135	0.172	20
Orifice width	0.141	0.010	0.119	0.161	20
Ovicell length	0.321	0.030	0.268	0.357	13
Ovicell width	0.342	0.018	0.316	0.378	13
Avicularium length	0.196	0.047	0.115	0.295	20
Avicularium width	0.043	0.007	0.034	0.062	20
Pore density	7	2	3	10	20

SD, Standard deviation; N, number of measurements.

*Type material*: Holotype: OLL 2015/1, *Meteor* 42/3, Stn 465, 05/09/1998, Great Meteor Bank, 29°48.3'N 28°36.5'W, 302 m, two colonies on coral, the larger elongated one being the holotype, the smaller colony is regarded as paratype, mounted on stub and sputter-coated, leg. B. Bader.

Paratypes *(locality information of all paratypes same as for holotype)*: NHMUK 2015.3.4.6, one colony on coral, mounted on stub and sputter-coated. NHMUK 2015.3.4.7, one colony on coral, dry. OLL 2015/2, one colony on coral, mounted on stub and sputter-coated. OLL 2015/3, one colony on coral, mounted on stub and sputter-coated. OLL 2015/4, one colony on coral, mounted on stub and sputter-coated. OLL 2015/5, three colonies on coral, dry. SMF 30.051, one colony on coral with *Hippothoa* sp., mounted on stub and sputter-coated. SMF 30.052, one colony on coral, dry.

*Other material examined*: See S2 Text.

*Etymology*: From the ancient Greek φτεροκοπώ (to flap one's wings, to flutter) in allusion to the zooids with their distolateral avicularia (spread wings) and the orifice (head) that give the impression of a flying bird.

*Description*: Colony encrusting, unilaminar or occasionally plurilaminar due to self-overgrowth, multiserial, the colony margin often lifting off the substrate and growing free.

Autozooids rectangular, rhombic or polygonal with sloping distolateral walls, relatively large, usually about as wide as long, separated by sutures on slightly raised, thin rims. Distolateral vertical walls with few, small, round communication pores.

Frontal shield flat or slightly convex, rising distally towards orifice, somewhat textured proximally and becoming granular distally, centrally and distally perforated by some 30, relatively large, round pores apart from suboral area.

Primary orifice situated above level of frontal plane on a slightly thickened rim, circular to horseshoe-shaped in outline, slightly longer than wide, proximal margin with a broadly U-shaped sinus occupying about half of total proximal width; condyles narrow but as long as proximal margin, slightly sloping, with 3 or 4 broad blunt denticles; distolateral orifice margin in autozooids with 9 or occasionally 8 oral spines (6 to 8 in colonies from Atlantis Seamount), 4 in ovicellate zooids.

Avicularia most often paired, situated directly at the distal border of the zooid, long and slender; rostrum length variable, occasionally extremely elongate, triangular, curved downward and/or proximally in distal half, pointing laterally or proximolaterally; distal uncalcified area elongate triangular and less than half of rostrum length, proximal area usually narrower and elliptical, margins with a sloping rim of calcification, distally forming a straight, basally directed extension underneath complete crossbar that may appear as a small columella when viewed from above. In colonies from Atlantis Seamount, additional small and triangular avicularia may occasionally appear along lateral sutures between zooids.

Ovicell hyperstomial, globular, flattened frontally, broader than long, ectooecium entirely exposed, perforated by numerous, usually round but occasionally fused and elongated, rimmed pores of irregular size, ovicell opening semicircular, not closed by the operculum.

Ancestrula oval, longer than wide (0.39 x 0.34 mm), gymnocyst well developed proximally, narrowing and steepening distolaterally; opesia composed of a non-calcified distal half and a slightly broader and partially calcified cryptocystal proximal part; cryptocyst very broad proximally, flat, smooth, with distolateral margins gently sloping towards the centre, forming proximomedially directing denticles that restrict a presumably broad and deep, U-shaped sinus; mural rim with 9 spines (4 lateral and distal pairs, a single proximal spine), with the 2 distal pairs of spines positioned slightly closer to each other, the third pair positioned level with 'condyles'; one relatively large round communication pore per neighbouring zooid.

*Remarks*: *Schizomavella* (*C*.) *phterocopa* n. sp. has been recorded on the Great Meteor Bank from depths of 300 to 416 m. Its sister species is *Schizomavella* (*C*.) *paucimandibulata* (d'Hondt, 1975) from the Azores (see below; Fig 1), as it is also lacking the suboral avicularium, while *S*. (*C*.) *paucimandibulata* differs in having fewer spines (6–7), a distinctly narrower sinus, and a greater pore density (12).

Colonies from Atlantis Seamount, which were recovered from 280–338 m depth and which lack ovicells, are tentatively assigned to *S*. (*C*.) *phterocopa* as well because their general autozooid, orifice, and avicularium morphologies and morphometrics are identical. Differences exist, however, in the number of oral spines, which number six to eight (most often seven) in Atlantis material, in contrast with eight to nine spines in the types from the Great Meteor Bank. Moreover, the autozooids in the two small colonies available from Atlantis Seamount are disordered, and additional avicularia may grow on the frontal shield along the lateral interzooidal sutures (Fig 10F). In colonies where autozooids are arranged in series (all coming from the Great Meteor Bank) these avicularia were not seen. Additional material from Atlantis Seamount is needed to shed light on the relatedness of these two populations.

In one colony from the Great Meteor Bank (M19-133/DD99), almost all orifices are damaged presumably during attempts by a predator to gain access to the polypide via the orifice. The orifices of numerous zooids in other colonies are filled by calcified closure plates, which may be another indication of predation. In contrast, intramural buds in otherwise undamaged zooids (cf. [44]) were rarely observed in any of the species analysed in this paper. If present, the orifice rim of the intramural bud was very narrow and lacking the oral spines, which makes repaired zooids difficult to spot in at least some *Schizomavella* species.

**Fig 12:**
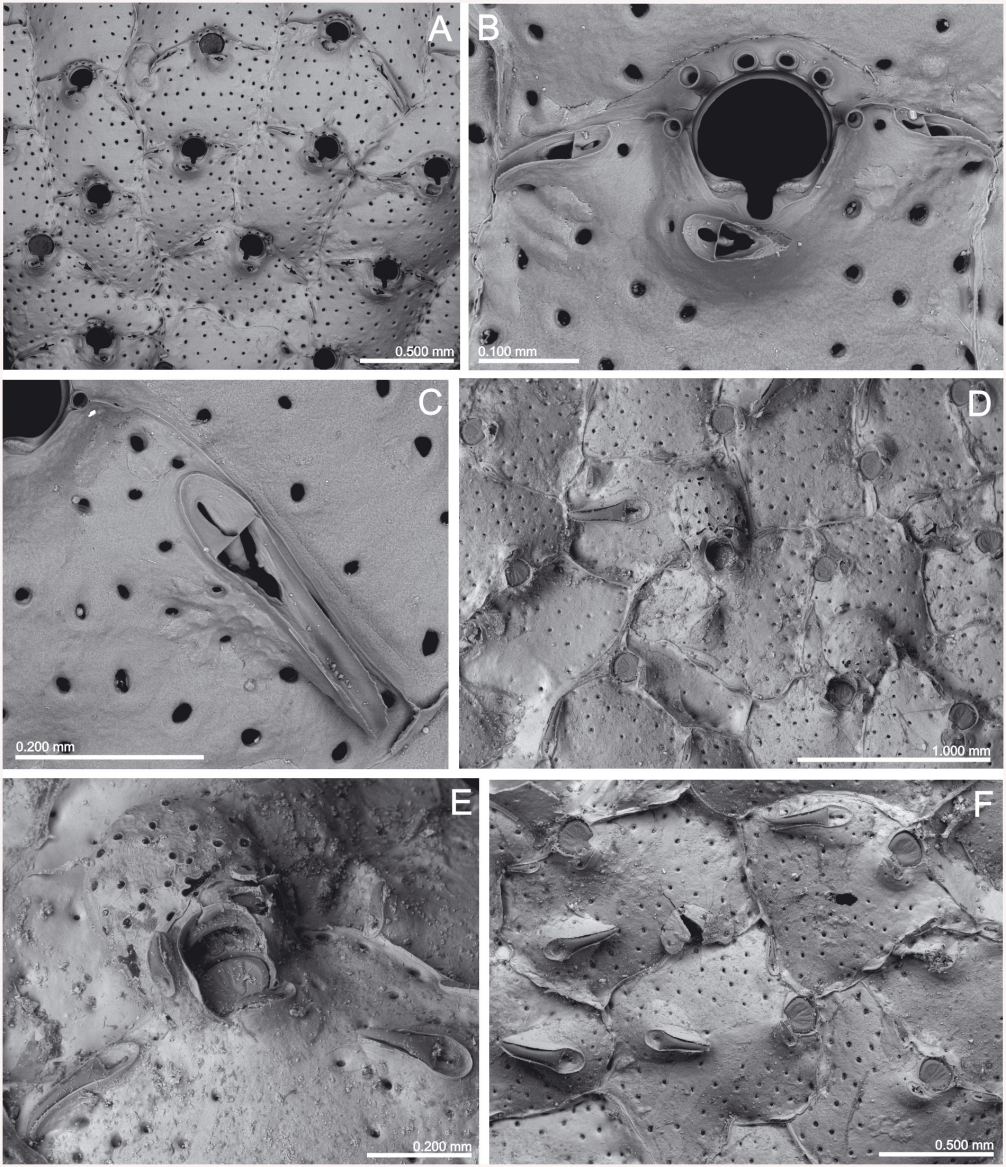
*Schizomavella (Calvetomavella) triaviculata* (Calvet *in* Jullien & Calvet, 1903). (A) Infertile zooids with the three distal avicularia (MNHN 3728, part of holotype); (B) same, primary orifice and avicularia; (C) same, detail of a distolateral avicularium; (D) detail of a multilaminar colony with disordered zooids, ovicells and enlarged avicularia (MOM INV-22670); (E) same, an ovicell with a pair of avicularia on the peristome; (F) same, enlarged avicularia.

**Fig 13:**
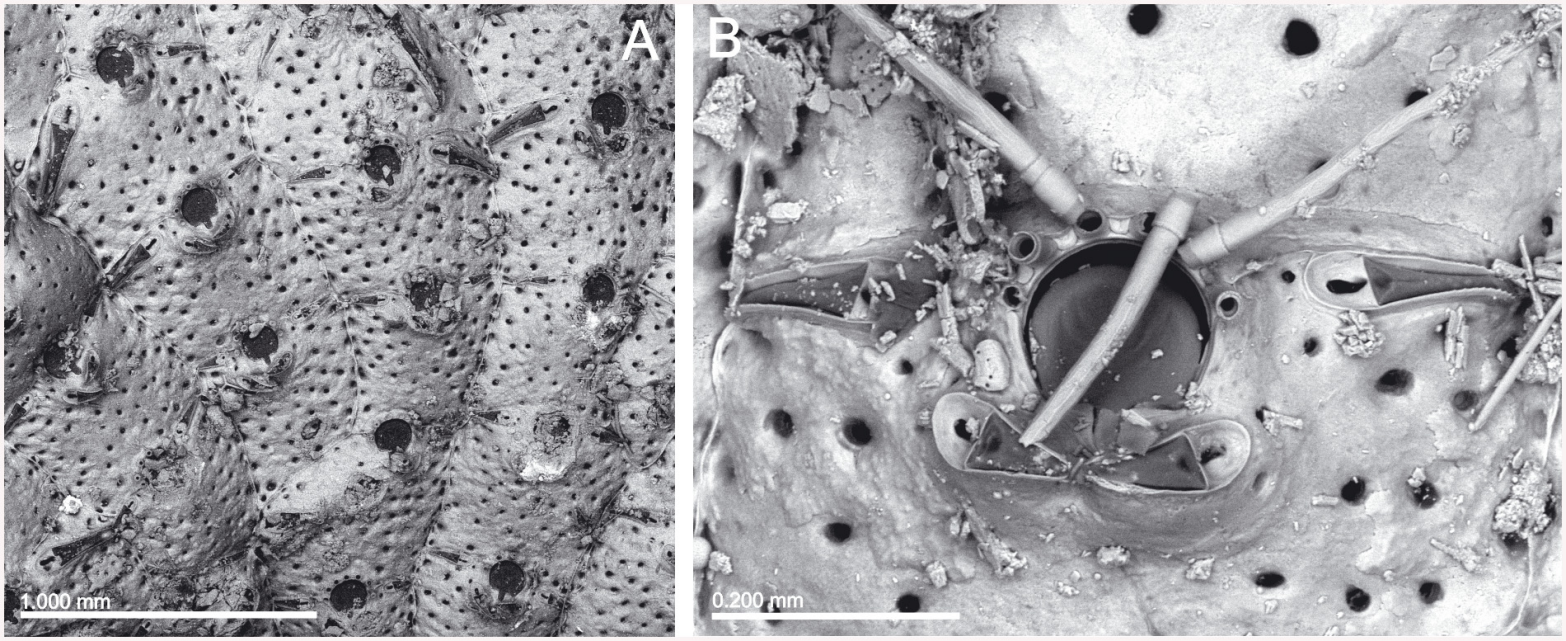
*Schizoporella triaviculata* var. *multimandibulata* d’Hondt, 1975 (MNHN 7549, holotype). (A) Detail of the colony; (B) primary orifice and avicularia.

**Fig 14:**
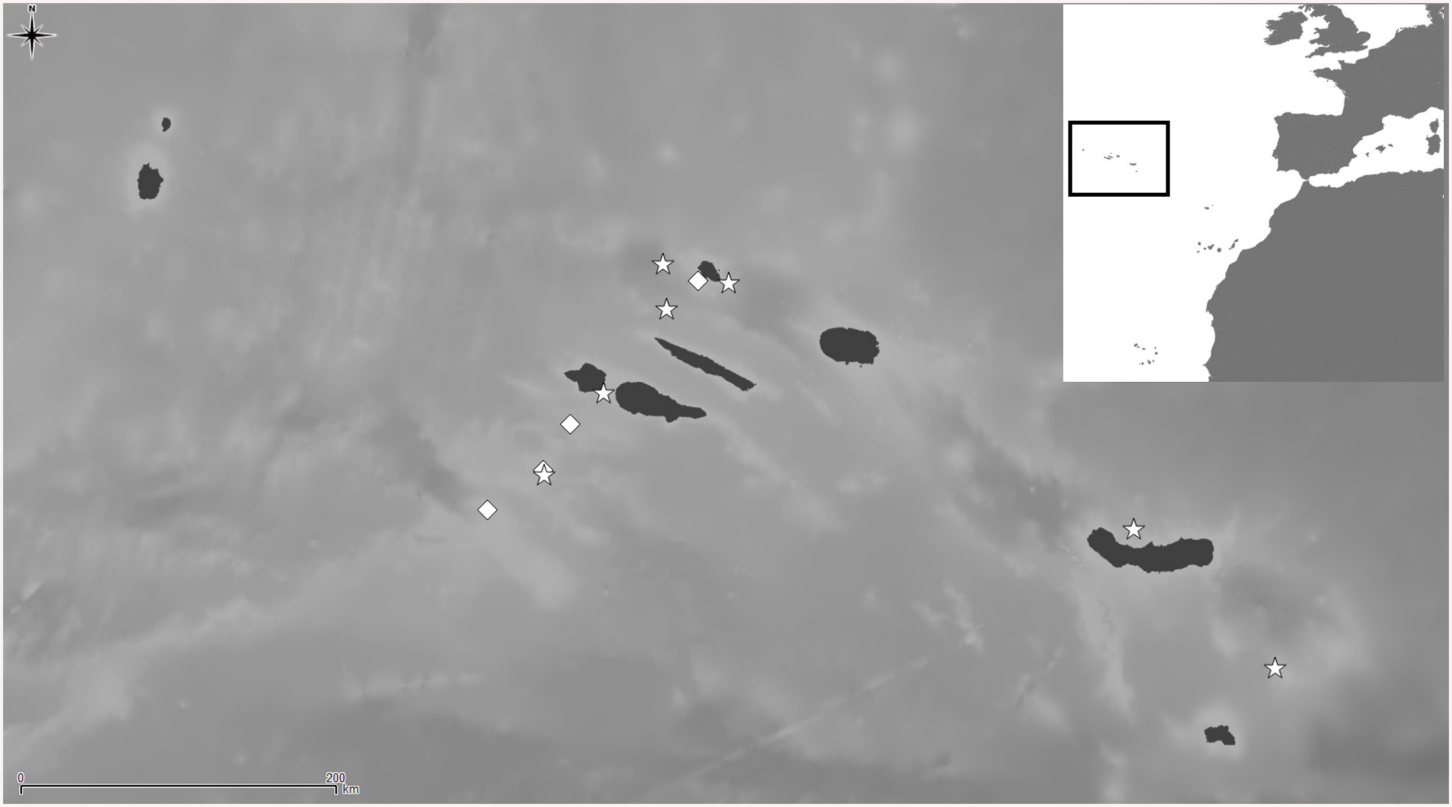
Distributional records of *Schizomavella (Calvetomavella) triaviculata* (Calvet *in* Jullien & Calvet, 1903) gathered from the literature and material examined. (Stars—material examined, diamonds—unconfirmed record taken from literature). Note that some symbols represent more than one record as the resolution of the map is insufficient to depict all records.

***Schizomavella (Calvetomavella) triaviculata*** (Calvet *in* Jullien & Calvet, 1903)

(Figs 12–14, Table 5)

**Table 5:** Measurements (in mm) of *Schizomavella (Calvetomavella) triaviculata* (holotype; MNHN 7457; MOM INV-22670).

	Mean	SD	Minimum	Maximum	N
Autozooid length	0.734	0.081	0.635	0.931	20
Autozooid width	0.750	0.122	0.524	0.959	20
Orifice length	0.150	0.007	0.133	0.163	20
Orifice width	0.142	0.005	0.132	0.149	20
Ovicell length	0.386	-	0.350	0.422	2
Ovicell width	0.398	-	0.386	0.410	2
Distal avicularium length	0.194	0.065	0.114	0.362	20
Distal avicularium width	0.046	0.012	0.030	0.077	20
Suboral avicularium length	0.112	0.013	0.090	0.134	20
Suboral avicularium width	0.044	0.003	0.037	0.050	20
Enlarged avicularium length	0.330	0.068	0.215	0.417	8
Enlarged avicularium width	0.106	0.023	0.061	0.128	8
Pore density	6	1	4	10	20

SD, Standard deviation; N, number of measurements.

*Schizoporella triaviculata* Calvet *in* Jullien & Calvet, 1903: 143, pl. 17, fig. 2a-e [3].

part *Schizoporella triaviculata* Calvet: Calvet, 1931: 83 (only material from Stn 568 and 600) [7].

*Schizoporella triaviculata* Calvet var. *typica* d’Hondt, 1975: 575 [18].

?*Schizoporella triaviculata* Calvet var. *multimandibulata* d’Hondt, 1975: 575, fig. 24 [18].

Not *Schizoporella triaviculata* Calvet: Calvet, 1907: 420, pl. 27, fig. 15 (as “*var*.” in the plate) [6]; Calvet, 1931: 83 (in part, material from Stn 584, 597 and 882) [7].

Part *Schizomavella triaviculata* (Calvet): Wisshak et al., 2015: 95 [22].

*Type material*: Holotype (by monotypy): formerly one large colony detached from substratum, divided into several fragments, *Hirondelle*, Pico Island (Azores; see remarks below), Calvet Coll. MNHN 3728, two fragments in a vial, one bleached; MOM INV-22506, one fragment on slide; MOM INV-22507, three fragments in ethanol.

*Other material examined*: See S2 Text.

*Description*: Colony encrusting, unilaminar to multilaminar, multiserial.

Autozooids rectangular to rhomboidal, about as broad as long, or broader than long, separated by sutures on slightly raised, thin rims. Distolateral vertical walls with several, small, round communication pores.

Frontal shield very slightly convex, surface smooth, evenly perforated by 20–30 small round pores.

Primary orifice slightly elevated, about as long as wide, anter subcircular, proximal margin with straight lateral edges and a very deep, narrow, U-shaped sinus occupying about one-third of total proximal width and of total orifice length; condyles relatively broad, exactly as long as proximal margin and usually slightly sloping towards rounded shoulders, a frontal structured part somewhat set off from a smooth rear part; distolateral orifice margin in autozooids with 5–6 oral spines.

A pair of adventitious avicularia, directly situated at and paralleling the distolateral border of the zooid, plus a single suboral one; additional avicularia of variable size may form on the proximolateral frontal shield during ontogeny. Distal avicularia slender, of variable length but generally much longer than suboral one, directed laterally or proximolaterally; rostrum elongate to extremely elongate depending on space along distal border, triangular, curved downward and/or proximally in distal part, faint serration may occur; distal foramen elongate triafoliate, proximal foramen a narrow slit, margins with a sloping rim of calcification, distally forming a tubular, basally directed extension underneath complete crossbar that may appear as a small columella when viewed from above. Suboral avicularium with distinctly shorter rostrum than distal avicularia but otherwise of similar morphology, directing laterally, positioned slightly offset relative to sinus: if pointing left it is shifted to the right of sinus and vice versa.

In areas where autozooids are disordered, additional small and triangular avicularia may appear along lateral interzooidal sutures. Enlarged avicularia may also occur on autozooids or kenozooids filling spaces between them.

Ovicells rare, hyperstomial, large, globular but depressed frontally, about as long as wide, ectooecium almost entirely exposed, perforated by numerous widely-spaced round to elongated pseudopores, peripheral ones usually slightly larger than central ones; ovicellate zooid developing a thin lateral peristome joining distally on the proximal ooecial margin; distal half of the peristome framed by one or two small, triangular, slightly curved avicularia; one or more larger avicularia may be present at the distolateral ooecial margin. Ovicell opening compressed. No spines visible in ovicellate zooids, but spines are present underneath the ovicell along the distal orifice margin.

An ancestrula was not observed.

*Remarks*: *Schizoporella triaviculata* was originally described by Calvet [3] based on a single large colony that is now divided into several fragments, two of them held at Paris (MNHN 3728) and four moreat Monaco (MOM INV-22506, -22507). Therefore, all of these samples must be considered as parts of the holotype of the species. The origin of this specimen is, however, doubtful. In a footnote, Calvet (*in* Jullien & Calvet, 1903: 143) [3] explained that the vial containing the specimen carried the label "Île de Pico (Açores), 1887" without specifying the station number. He then concluded that the specimen must be from Stn 108, which corresponds to manually collected samples from the intertidal near Lajes do Pico. For several reasons, the correctness of Calvet's conclusion has to be regarded as highly questionable. For once, the next deeper record of *S*. *triaviculata s*.*s*. (see below for problems with the species) is from settlement panels deployed at 150 m (OLL 2015/20), while the species was absent from settlement panels at 60, 15, 5 and 0 m [22]. It was most often recorded around 360 m, and its deepest occurrence is 550 m. Moreover, no other bryozoan species were reported from Stn 108, and hence the station was not given in the station list provided by Calvet *in* Jullien & Calvet (1903: 170) [3]. Finally, the presence of a large, unilaminar encrusting colony that occurs free of its substrate in such a shallow setting is highly unlikely. Especially in the Azores, intertidal bryozoans are absent or extremely rare owing to high wave energy and abrasion rates (BB, pers. oberv.). Although some embayments at Lajes do Pico are protected from wave swell, the rocky bottom of these sites is usually covered in soft algae and highly mobile rocks and rhodoliths. Accordingly, the species could not be found in a recent visit to the site by one of us (BB, 2015). We thus regard the purported type location (Stn 108, intertidal, at Lajes do Pico) as erroneous.

Calvet [6] later reported a specimen from the Pico-Faial channel as a variety of *S*. *triaviculata*. This variety was subsequently described as? *Schizoporella triaviculata* var. *paucimandibulata* by d’Hondt [18], which is raised to species status in the present paper (see below). Later again, Calvet [7] reported more material from the Azores. Samples from Stn 568 and 600, collected between 394 m and 550 m depth, correspond to *S*. *triaviculata s*.*s*., as already stated by the author. Material from Stn 584, 597 and 882, reported by Calvet (1931: 83) [7] as belonging to the “*variété du Travailleur et du Talisman*”, correspond to the variety described by d’Hondt [18] (see below). The species was not reported again until d’Hondt [18] collected it from several localities in the Azores between 142 m and 580 m depth. In the recent settlement panel experiment off Faial Island mentioned above [22], only some of the colonies recorded as *S*. (*C*.) *triaviculata* belong to this species, while several others, which were not distinguished during quantitiative analysis using an optical microscope, are in fact *Schizomavella* (*C*.) *paucimandibulata* (see below).

The holotype of *S*. *triaviculata* consists in a large unilaminar colony, but frontal budding may occasionally occur in other samples (e.g. MNHN 7457, MOM INV-22670, see Fig 12D–12F). Autozooids are then irregular in outline, disorganised and interspersed with irregular kenozooids. In these areas, small triangular avicularia may grow just by the lateral interzooidal sutures, and enlarged avicularia may also occur on autozooids and kenozooids.

From the Formigas Islets, situated between the Azorean islands Sao Miguel and Santa Maria, d’Hondt [18] furthermore described the variety *multimandibulata* based on a single colony devoid of ovicells that is characterised by the presence of two suboral avicularia instead of a single one (MNHN 7549, Fig 13). This pair of avicularia is not constant, being present in about half of the autozooids. Moreover, they are slightly curved (a character not seen in *S*. *triaviculata s*.*s*.) and the number of oral spines is greater than in other specimens (6–8). However, these differential characters do not seem enough as to upgrade the variety to species status, at least until more ovicellate material can be studied.

*Schizomavella triaviculata* differs from *S*. *phterocopa* n. sp., as well as from the remaining species described here, most obviously by the suboral avicularium directing laterally, and the thin peristome in ovicellate zooids, covered by small distolateral avicularia; also by the narrower sinus. Moreover, in both species additional avicularia may occur along lateral interzooidal sutures in areas where zooids grow disordered, but *S*. *phterocopa* n. sp. seems to lack the enlarged avicularium characteristic of *S*. *triaviculata*. Both species share, however, some characters that allow placing *S*. *triaviculata* in *Calvetomavella* n. subgen.

*Schizomavella discoidea s*.*l*. (Figs 2 and 22B) also has ovicellate zooids that develop a lateral peristome, lack oral spines, and may occasionally carry a single suboral avicularium, which is always oval and proximally directed. Moreover, the distolateral avicularia are not placed at the distolateral border of the autozooid but slightly proximal to it.

**Fig 15:**
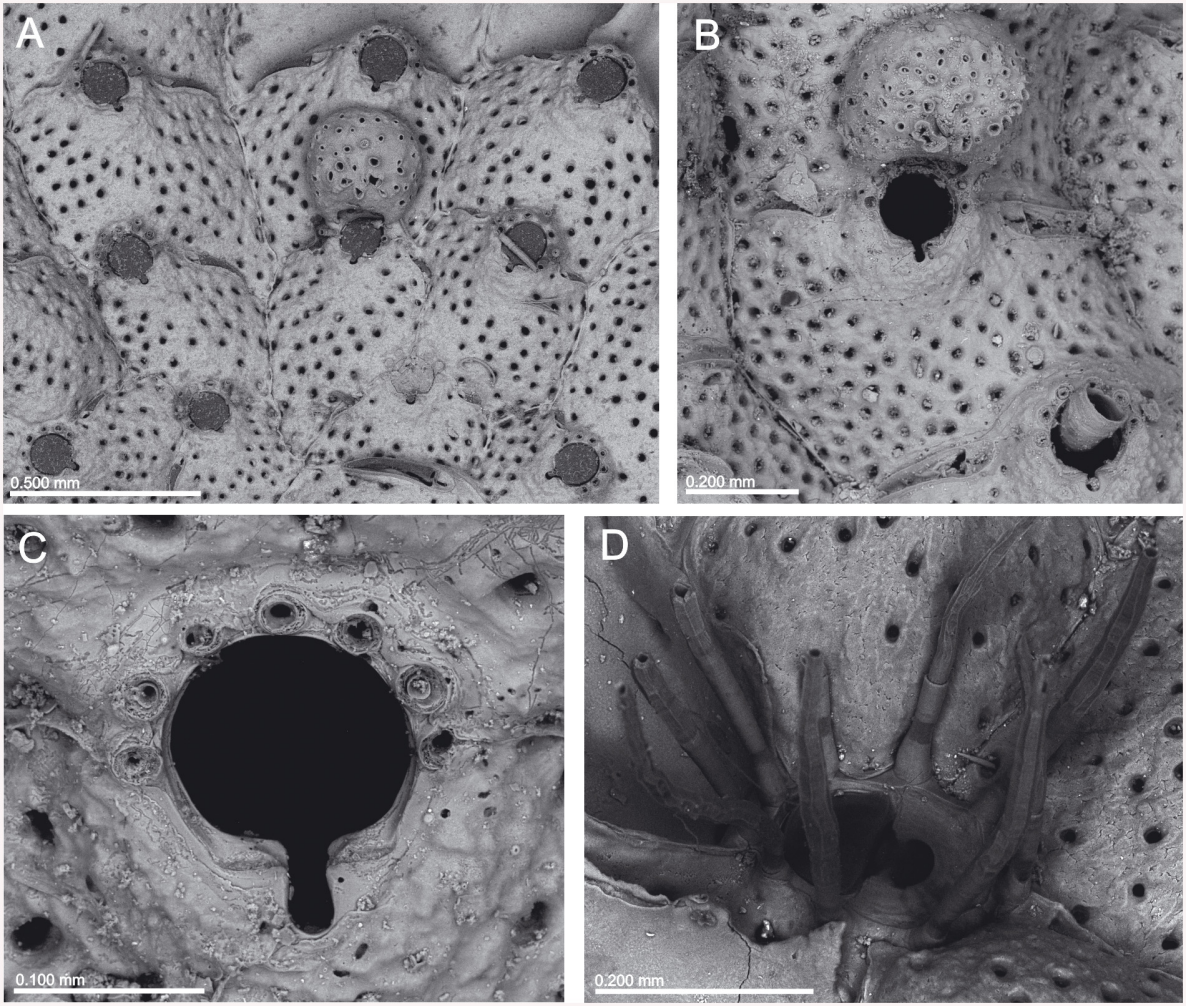
*Schizomavella (Calvetomavella) paucimandibulata* (d’Hondt, 1975) n. comb. (A) Group of zooids (MNHN 4296); (B) an ovicellate zooid (MNHN 3771, holotype); (C) same, primary orifice; (D) detail of the ancestrula (unregistered specimen from settlement panel, leg. M. Wisshak).

**Fig 16:**
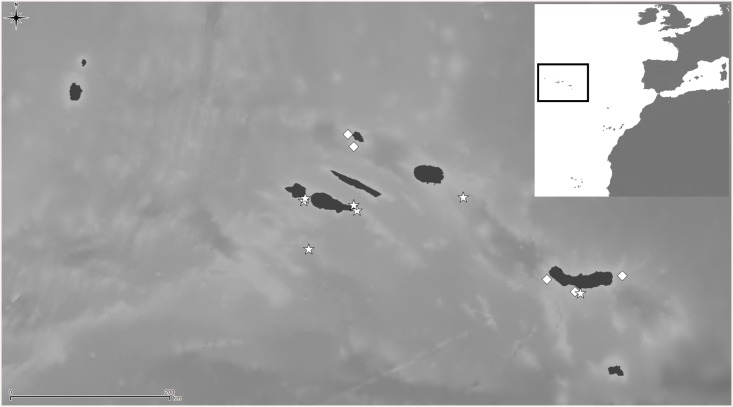
Distributional records of *Schizomavella (Calvetomavella) paucimandibulata* (d’Hondt, 1975) n. comb. gathered from the literature and material examined. (Stars—material examined, diamonds—unconfirmed record taken from literature). Note that some symbols represent more than one record as the resolution of the map is insufficient to depict all records.

***Schizomavella (Calvetomavella) paucimandibulata*** (d’Hondt, 1975) **n. comb.**

(Figs 15 and 16, Table 6)

**Table 6:** Measurements (in mm) of *Schizomavella (Calvetomavella) paucimandibulata* n. comb. (MNHN 3771, 4021, 4296).

	Mean	SD	Minimum	Maximum	N
Autozooid length	0.661	0.075	0.534	0.798	20
Autozooid width	0.720	0.123	0.521	0.923	20
Orifice length	0.165	0.008	0.149	0.176	20
Orifice width	0.132	0.008	0.111	0.142	20
Ovicell length	0.289	0.009	0.277	0.299	8
Ovicell width	0.321	0.031	0.274	0.367	8
Avicularium length	0.199	0.057	0.120	0.302	20
Avicularium width	0.056	0.014	0.034	0.092	20
Pore density	12	3	8	16	20

SD, Standard deviation; N, number of measurements.

*Schizoporella neptuni* Jullien: Jullien & Calvet, 1903: 80 [3].

*Schizoporella triaviculata* Calvet: Calvet, 1907: 420, pl. 27, fig. 15 (as “*var*.” in the figure caption) [6].

part *Schizoporella triaviculata* Calvet: Calvet, 1931: 83 (only material from Stn 584, 597 and 882) [7].

*Schizoporella triaviculata* var. *paucimandibulata* d’Hondt, 1975: 575 [18].

Not *Schizoporella triaviculata* Calvet: Calvet, 1931: 83 (in part, material from Stn 568 and 600) [7].

Part *Schizomavella triaviculata* (Calvet): Wisshak et al., 2015: 95 [22].

*Type material*: Holotype (by monotypy): MNHN 3771, one colony on bivalve shell fragment, *Talisman*, Stn 125, between Pico and Faial (Azores), 38°29.1'N 28°37.33'W, 13/08/1883, 80–115 m, Calvet Coll.

*Other material examined*: See S2 Text.

*Description*: Colony encrusting, unilaminar, multiserial.

Autozooids rectangular to polygonal, usually broader than long, separated by sutures on slightly raised, thin rims. Distolateral vertical walls with several, small, round communication pores.

Frontal shield slightly convex, rising in distal part towards orifice, surface rugose, perforated by 40–50 (even up to 70) relatively large, round pores apart from the raised suboral area.

Primary orifice longer than wide, situated above level of frontal plane on a thickened rim; anter circular in outline, proximal margin with straight lateral edges and a deep to extremely deep, narrow, U-shaped or somewhat drop-shaped sinus occupying about one-fifth of total proximal width, and almost one-third of total orifice length; condyles relatively broad, as long as proximal margin and very gently sloping towards rounded shoulders, these shoulders often somewhat partitioned into a more gently sloping frontal part and a rear part forming the pronounced condyle edges. Distolateral orifice margin in autozooids with 7 or occasionally 6 oral spines, 2 in ovicellate zooids although bases of another pair may occasionally be visible.

Avicularia adventitious, always paired, situated directly at the distal border of the zooid, slender, of variable length; rostrum short to extremely elongate depending on space along distal border, triangular, directing laterally or proximolaterally, curved downward and/or proximally in distal half; distal uncalcified foramen elongate triangular, proximal foramen narrower and elongate elliptical, margins with a sloping rim of calcification, distally forming a straight, basally directed extension underneath complete crossbar that may appear as a small columella when viewed from above.

Ovicell prominent, recumbent on distal autozooid, globular, slightly broader than long, ectooecium entirely exposed, perforated by numerous round to elongated pores of irregular size that are encircled by a raised and occasionally flared rim; ovicell opening semicircular, not closed by the operculum.

Ancestrula slightly longer than wide, mural rim with 9 spines, proximal half of intramural area occupied by a broad, flat, cryptocystal shelf, its distolateral margin straight with a pair of strong 'condyles' directing proximobasally, constricting a central U-shaped opesia and separating it from a D-shaped distal opesia that is entirely occupied by the operculum in life.

*Remarks*: Although *Schizomavella* (*C*.) *paucimandibulata* had already been sampled, and misidentified as *S*. *neptuni*, at 318 m depth off Pico Island (Azores) by Jullien & Calvet (1903: 80) [3] (MOM INV-22537), the species was recognised for the first time by Calvet [6] as an unnamed variety of *Schizoporella triaviculata*. The author stated that the single colony, collected in the Pico-Faial Channel at 80–115 m depth, differed from *S*. *triaviculata s*.*s*. only by the absence of the suboral avicularium, and by the presence of ovicells, which are absent in the type material of *S*. *triaviculata* (however, ovicells do exist in this species; see above). Although Calvet did not give a name to this variety, in the figure caption (pl. 27, fig. 15) it is given as “*Schizoporella triaviculata* Calvet var.”. Later, Calvet [7] reported more material of *S*. *triaviculata* from the Azores. Samples from Stn 568 and 600 correspond to *S*. *triaviculata s*.*s*. (see above), while material from Stn 584, 597 and 882, collected between 98 m and 845 m depth, were reported as belonging to the “*varieté du Travailleur et du Talisman*”. Then d’Hondt [18] described the variety *paucimandibulata* for Calvet's material, while reporting some new material from several stations in the Azores. Finally, most of the colonies reported as *S*. (*C*.) *triaviculata* from settlement panels deployed off Faial Island at 150 m depth by Wisshak et al. [22], are in fact *S*. (*C*.) *paucimandibulata*.

D'Hondt's [18] original variety *paucimandibulata* differs from *S*. *triaviculata s*.*s*. as defined in the present paper (see above) not only by the absence of a suboral avicularium but also by the thickened periorbital rim; a higher number of oral spines, two or four of which are present in ovicellate zooids; the deeper and somewhat drop-shaped sinus; a greater number of larger frontal pores and a greater pore density (12 vs. 6); by ectooecial pores that are encircled by a raised and occasionally flared rim; and by the absence of a peristome in ovicellate zooids. Consequently, the variety *paucimandibulata* is here raised to species status. A fair amount of variability as concerns sinus and condyle shape is, nevertheless, present between the colonies assigned to this species. For instance, the colonies recorded by Wisshak et al. [22] from more or less the type location lack the conspicuous condyle shoulders that are present in the holotype.

*Schizomavella (C*.*) phterocopa* n. sp. is very similar to *S*. *(C*.*) paucimandibulata*, but differs in having a much wider sinus with divergent lateral edges, more oral spines (8–9 versus 6–7), and fewer frontal pores and pore density (7 vs. 12).

*Schizomavella (C*.*) paucimandibulata* must be classified within the subgenus *Calvetomavella* owing to the presence of a high number of oral spines in all autozooids, while 2 or 4 are visible in ovicellate ones, the reduction of frontal calcification, the presence of a pair of distolateral avicularia, and the structure of the ovicell.

*Schizomavella (C*.*) paucimandibulata* is only known from a small number of localities in the Azores. As *S*. *(C*.*) triaviculata*, the species has been sampled from a great vertical range, between 80 m and 845 m depth.

**Fig 17:**
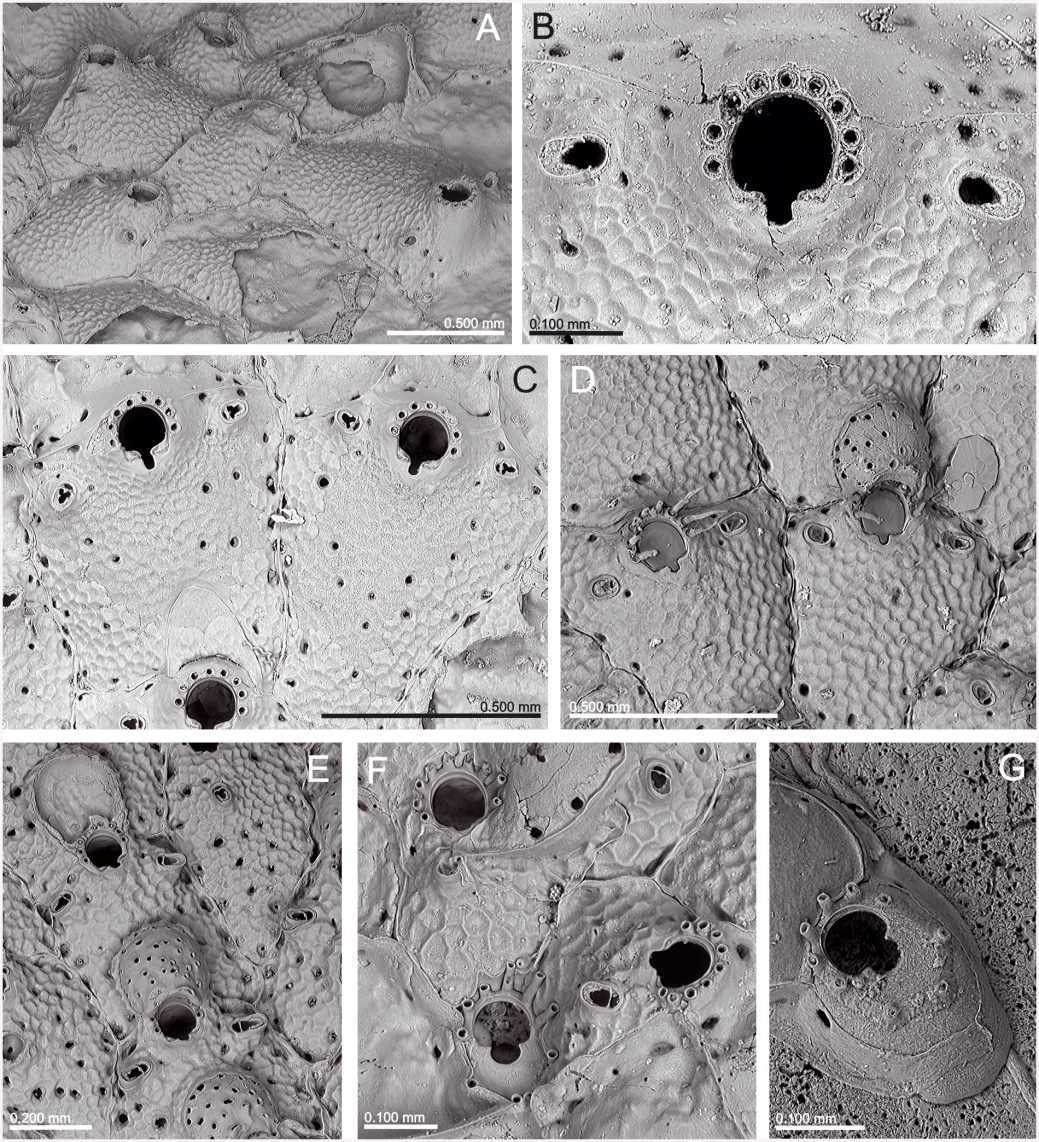
*Schizomavella (Calvetomavella) neptuni* (Jullien, 1882). (A) Group of zooids; note the raised periorbital region (MNHN 2342, lectotype); (B) same, primary orifice and avicularia; (C) infertile zooids (MNHN 4097); (D) two zooids, one ovicellate (MNHN 7653); (E) ovicellate zooids with slightly enlarged avicularia (MNHN IB-2013-579); (F) same, ancestrula and periancestrular zooids; (G) same, ancestrula and first budded zooid.

**Fig 18:**
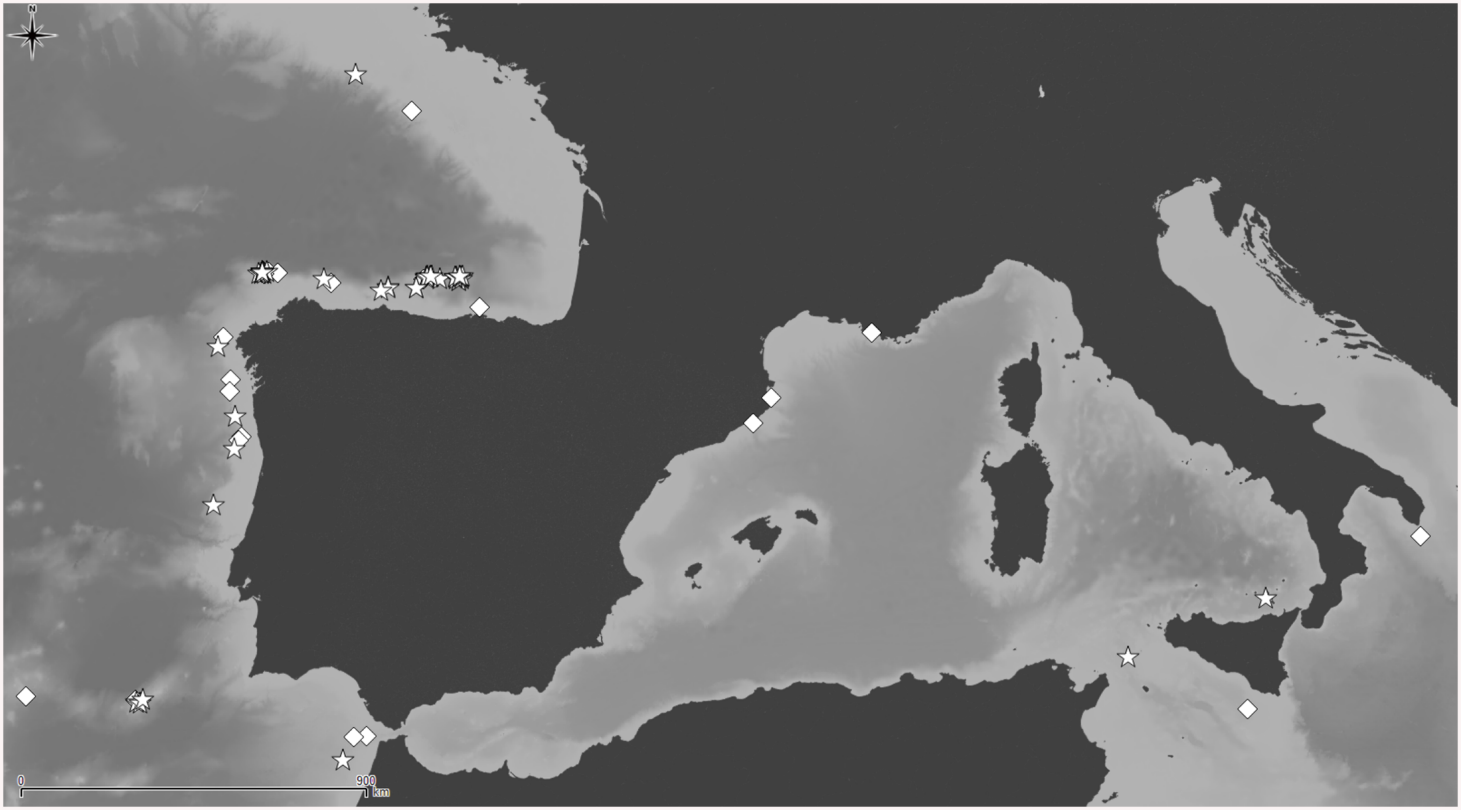
Distributional records of *Schizomavella (Calvetomavella) neptuni* (Jullien, 1882) gathered from the literature and material examined. (Stars—material examined, diamonds—unconfirmed record taken from literature). Note that some symbols represent more than one record as the resolution of the map is insufficient to depict all records.

***Schizomavella (Calvetomavella) neptuni*** (Jullien, 1882)

(Figs 17 and 18, Table 7)

**Table 7:** Measurements (in mm) of *Schizomavella (Calvetomavella) neptuni* n. comb. (Lectotype and MNHN 7653).

	Mean	SD	Minimum	Maximum	N
Autozooid length	0.699	0.1574	0.531	0.971	11
Autozooid width	0.552	0.1018	0.427	0.707	11
Orifice length	0.127	0.0125	0.108	0.151	11
Orifice width	0.112	0.0120	0.099	0.143	11
Ovicell length	0.216	0.0056	0.211	0.222	4
Ovicell width	0.249	0.0164	0.227	0.267	4
Avicularium length	0.069	0.0060	0.060	0.082	22
Avicularium width	0.042	0.0050	0.034	0.053	22
Pore density	2	1	0	3	20

SD, Standard deviation; N, number of measurements.

*Schizoporella neptuni* Jullien, 1882: 15, pl. 14, fig. 34 [1]; Jullien, 1883: 511, pl. 14, fig. 34 [2].

*Schizoporella neptuni* Jullien: Calvet, 1907: 421 (in part), pl. 27, fig. 14 [6]; d’Hondt, 1974: 40 [36]; Hayward & Ryland, 1978: 146 [45]; Hayward, 1979: 60 [28]; Zabala & Maluquer, 1988: 133, text fig. 314, pl. 18, fig. F [46].

*Schizomavella neptuni* (Jullien): Harmelin & d’Hondt, 1992: 46, pl. 6, fig. C [30]; Zabala et al., 1993: 75, figs 19, 20 [47]; Reverter-Gil & Fernández-Pulpeiro 2001: 119 [34]; Reverter-Gil et al., 2014: 25 [37].

?*Schizoporella neptuni* Jullien: d’Hondt, 1973: 1217 [48].

Not *Schizoporella neptuni* Jullien: Jullien & Calvet, 1903: 80 [3].

Not *Schizoporella neptuni* Jullien: Calvet, 1907: 421 (in part) [6].

Not *Schizomavella neptuni* (Jullien): Gordon, 1984: 81, pl. 27 D [29].

*Type material*: Lectotype (selected here): MNHN 2342, *Travailleur* Stn 2 (1^er^ sér.), 41°43'00''N 09°19'26''W, off N Portugal, 14/06/1881, 1068 m, one small colony on a stone, Jullien Coll.

*Other material examined*: See S2 Text.

*Description*: Colony encrusting, unilaminar, multiserial, forming irregular crusts.

Autozooids in regular linear series, sometimes alternating; polygonal, broad, separated by sutures on slightly raised, thin rims. Distolateral vertical walls with few small round communication pores.

Frontal shield slightly convex, rising in distal part towards orifice, entirely covered by polygonal pits separated by raised ridges; a row of small marginal pores plus up to 12 scattered small pores, frequently less; suboral area imperforate.

Primary orifice slightly longer than wide, situated above level of frontal plane on a thickened rim; anter orbicular; proximal margin with straight lateral edges and a U-shaped sinus, as long as wide, occupying about one-fourth of total proximal width; condyles relatively broad, as long as proximal margin and sloping towards the edge of the sinus. Distal half of orifice projecting beyond distolateral zooidal margin, surrounded by 7–10 stout, jointed spines, slender and sharp, very long but often disarticulated; the two most proximal pairs visible in ovicellate zooids.

Avicularia small, paired or more seldom single, positioned at orifice level or more proximally, near the lateral borders of the zooid; situated on a slightly raised cystid, directing laterally or proximolaterally, oblique to the frontal surface of the zooid. Rostrum semielliptical, with a Y-shaped foramen; complete crossbar with a small columella.

Ovicell prominent, globular, slightly wider than long; ectooecium nearly entirely exposed, perforated by numerous round to elongated pores of irregular size that are encircled by a raised rim, secondary calcification of distal zooid encroaching only the very margin of the distolateral ooecium; ovicell not closed by the operculum.

Ancestrula oval, longer than wide (0.29 x 0.22 mm); gymnocyst well developed proximally, narrowing and steepening distolaterally. Opesia mushroom-shaped, with a large U-shaped sinus, constricted proximolaterally by a well developed flat cryptocyst, surrounded by 9 mural spines, with the two distal pairs positioned slightly closer to each other, a third pair positioned level with the sinus of the opesia, and three proximal spines. First autozooid budded distally, plus two zooids budded distolaterally. One round communication pore per neighbouring zooid.

*Remarks*: *Schizoporella neptuni* was originally described by Jullien [1] from two stations off the NW Iberian Peninsula (*Travailleur* Stns 2 and 42) between 896 and 1068 m depth. Later, Calvet [6] only listed the first station, perhaps because the only preserved colony (MNHN 2342) was collected there. Therefore, it is not the holotype of the species, as wrongly stated by [42]. This colony, figured by Jullien (1882, pl. 14, fig. 34) [1] is here designated as the lectotype.

*Schizoporella neptuni* was later reported by Jullien & Calvet [3] from the Azores, but the original material (MOM INV-22537) actually corresponds to *S*. *paucimandibulata* n. sp. (see above).

Calvet [6] also reported four colonies of *S*. *neptuni* from near Cape Spartel (Strait of Gibraltar), but only two of them (MNHN 1022, MNHN 4097) indeed belong to the species (see remarks of *S*. *(S*.*) rectangularis* n. sp.). Calvet [6] also described the ovicell for the first time and made some corrections to the original description.

The species was not reported again until it was mentioned by d’Hondt [48] from material collected during the *Noratlante* survey at Gorringe Bank and Josephine Seamount at 600 m and 690 m depth, respectively. However, the avicularia are described as pointed instead of oval. As we were not able to find any of the original samples, the record has to remain doubtful. In any case, *S*. *neptuni* actually does exist at the Gorringe Bank (see Material examined).

D’Hondt [36] also collected *S*. *neptuni* in the NW of the Iberian Peninsula between 250 and 1130 m depth. Soon after, Hayward & Ryland [45] and Hayward [28] also reported the species from the northern and southern Bay of Biscay between 250 m and 910 m depth. In the Atlantic, *S*. *neptuni* was subsequently reported from the Gulf of Cadiz at 150–532 m depth by Harmelin & d’Hondt [30], and from the NW Iberian Peninsula between 35 and 930 m depth by Reverter-Gil & Fernández-Pulpeiro [34] and Reverter-Gil et al. [37].

*Schizomavella neptuni* was reported for the first time in the Mediterranean by Harmelin [38] from the strait between Sicily and Malta at 320–900 m depth. Subsequent records include caves in the Medes Isles (Catalonia) at 20 m depth [46], the Blanes Canyon, Provence and Tyrrhenian Sea between 120–700 m depth [47], a cave at 15 m depth in the Gulf of Lion [49], and recently at 513 m depth in the Ionian Sea [39,40]. In the absence of SEM images from most of the works, the assignment of these records to *S*. *neptuni* must remain tentative.

Although Zabala et al. [47] stated that *S*. *neptuni* is rare, it seems to be the most ubiquitous among the species treated in the present work. D’Hondt [36] also stated that it was one of the most abundant species in the area studied by the *Thalassa* expedition. In the Atlantic it is frequently found below 400 m depth. One of the localities in Portugal (see sample MB37-000030, and [37]) was indicated as coming from 35–930 m depth, although *S*. *neptuni* was probably sampled at the deeper end of the dredge range, as in a nearby locality the depth was 800–900 m (MB37-000037: *Poseidon* Stn 2). In the Mediterranean *S*. *neptuni* was recorded at 15 m and 20 m depth, but in both cases from sublittoral caves, probably in particular conditions simulating deep-sea environments; in open waters the minimum depth was 120 m, but it also seems to be more frequent in deeper waters.

Gordon [29] was the first author transferring *S*. *neptuni* to the genus *Schizomavella*. This record from Kermadec Ridge (Macauley Island, 290 m), which indeed differs in several aspects from the type of *S*. *neptuni*, was subsequently synonymised by [50] with his newly described species *Schizomavella pseudoneptuni* from New Caledonia. However, the taxon recorded by Gordon [29] was really based on two different species (D. Gordon pers. comm.), which are specifically different again from *S*. *pseudoneptuni*. A redescription of these SW Pacific species is, however, beyond the scope of the present paper.

*Schizomavella (C*.*) neptuni* differs from the other species here assigned to *Calvetomavella* n. subgen. in the shape of the orifice, the large number of spines, the narrow sinus, and the pair of small, oval avicularia with a Y-shaped distal foramen and the crossbar with a small columella. This species seems to show a low variability, mostly concerning sinus depths, even between Atlantic and Mediterranean material. However, although avicularia are in general quite small (Fig 17A–17D) in some material from the Gorringe Bank these are clearly longer (Fig 17E).

As stated by Berning [15], *S*. *neptuni* and *S*. *noronhai* are sibling species, both having a large number of oral spines, as well as similar avicularia, frontal wall surface structure, and ovicell. *Schizomavella noronhai*, here assigned to the subgenus *Calvetomavella* n. subgen., differs in the shape of its primary orifice, which is more quadrangular and with a wider sinus and smaller condyles, a larger number of larger frontal pores, and in the absence of a columella in the crossbar of the avicularium. Both species differ from the other species in *Calvetomavella* n. subgen. by the small, oval avicularia situated at orifice level or more proximally instead of being triangular and situated parallel to the distal zooidal margin. In *S*. *discoidea s*.*l*. the rostrum is also not pointed, and avicularia may be occasionally small and oval, and placed suborally or distolaterally.

Family **LANCEOPORIDAE** Harmer, 1957

Genus ***Stephanotheca*** Reverter-Gil, Souto & Fernández-Pulpeiro, 2012

**Fig 19:**
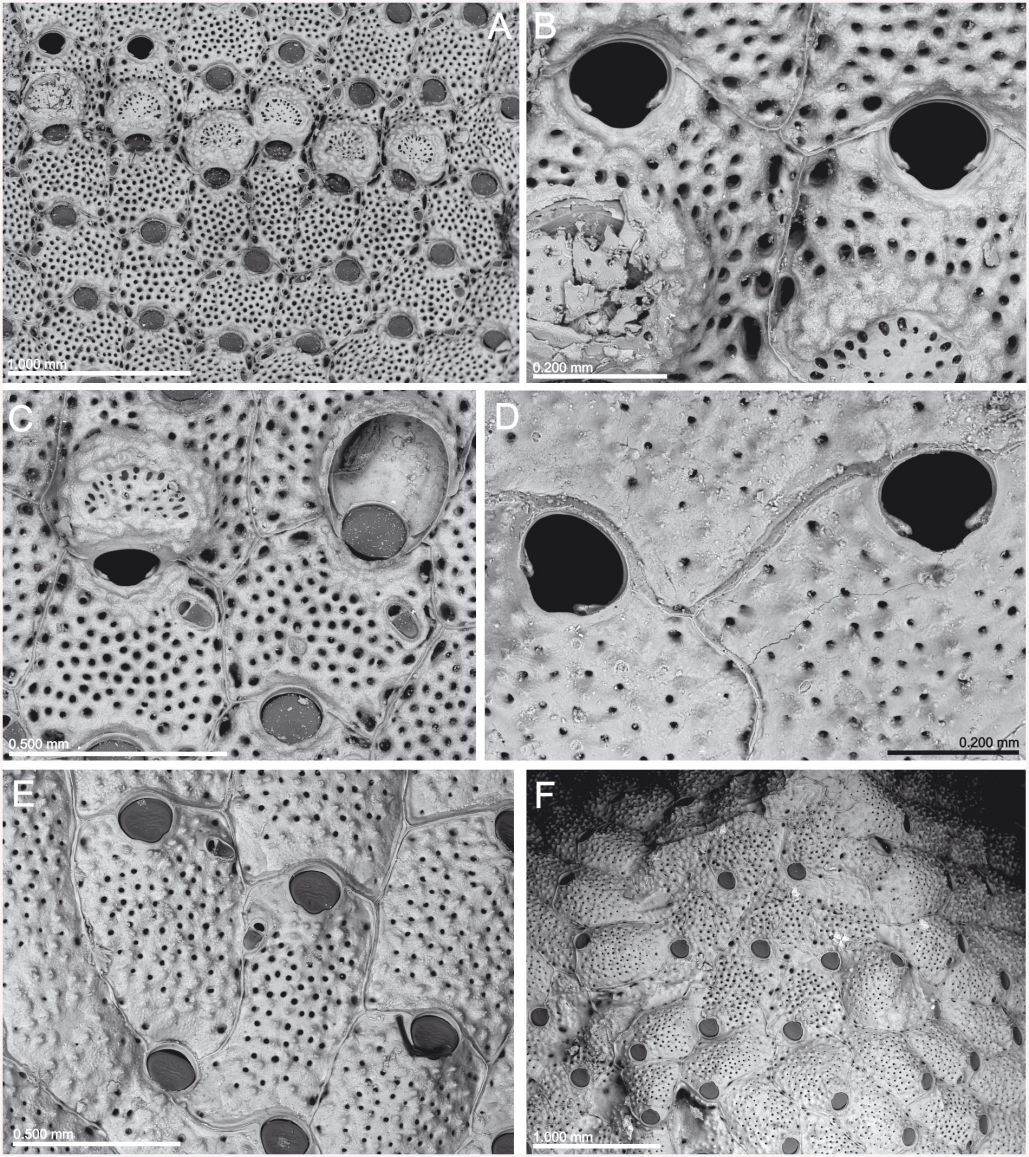
*Stephanotheca fayalensis* (Calvet *in* Jullien & Calvet, 1903) n. comb. (A) Detail of the colony (MNHN IB-2009-1553, lectotype); (B) same, primary orifices; (C) same, close-up of a condyle; note the slightly serrate inner margin; (D) same, ovicellate zooids and avicularia; (E) primary orifices in a free-living colony; note the smaller pores (MNHN 7547); (F) same, infertile zooids and avicularia; (G) same, apical budding in a free-living colony.

**Fig 20:**
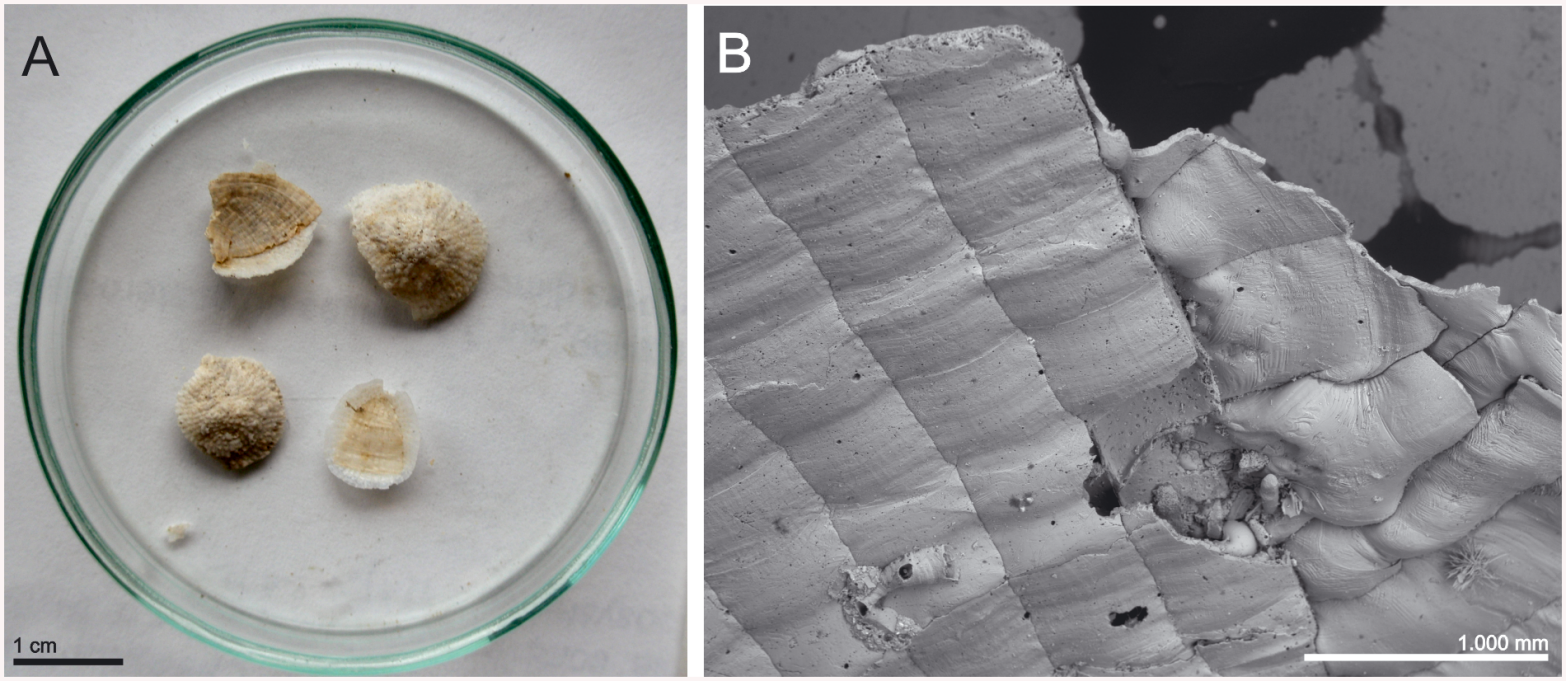
*Stephanotheca fayalensis* (Calvet *in* Jullien & Calvet, 1903) n. comb. (MNHN 7547). (A) Four free-living, discoidal colonies; (B) lateral budding in a free-living discoidal colony.

**Fig 21:**
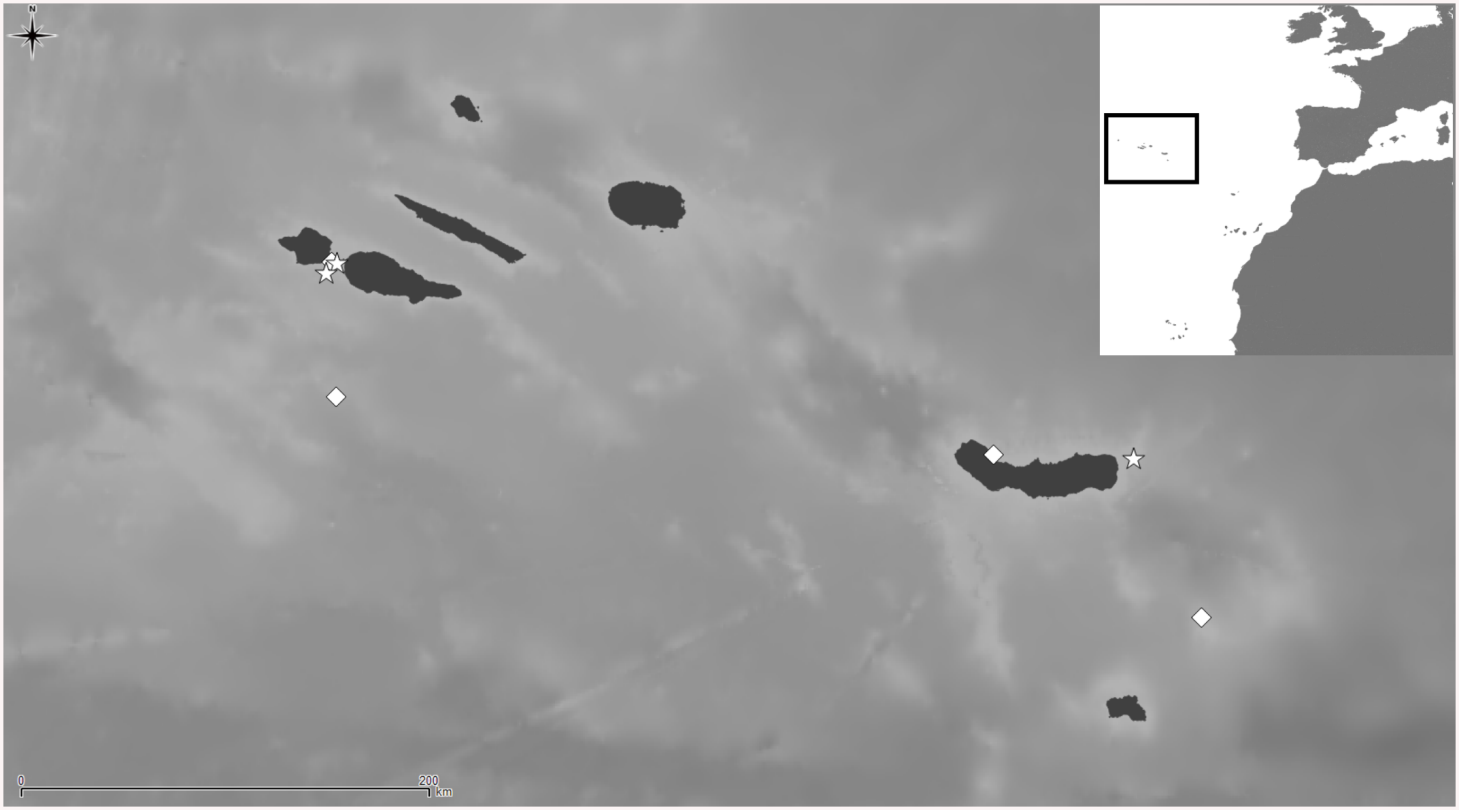
Distributional records of *Stephanotheca fayalensis* (Calvet *in* Jullien & Calvet, 1903) n. comb. gathered from the literature and material examined. (Stars—material examined, diamonds—unconfirmed record taken from literature). Note that some symbols represent more than one record as the resolution of the map is insufficient to depict all records.

***Stephanotheca fayalensis*** (Calvet *in* Jullien & Calvet, 1903) n. comb.

(Figs 19–21, Table 8)

**Table 8:** Measurements (in mm) of *Stephanotheca fayalensis* n. comb. (Lectotype).

	Mean	SD	Minimum	Maximum	N
Autozooid length	0.576	0.050	0.525	0.695	20
Autozooid width	0.492	0.048	0.406	0.599	20
Orifice length	0.128	0.005	0.118	0.136	20
Orifice width	0.138	0.008	0.122	0.149	20
Ovicell length	0.356	0.018	0.317	0.385	20
Ovicell width	0.366	0.019	0.334	0.407	20
Avicularium length	0.098	0.007	0.080	0.110	20
Avicularium width	0.051	0.005	0.042	0.057	20
Pore density	20	2	16	24	20

SD, Standard deviation; N, number of measurements.

*Schizoporella fayalensis* Calvet *in* Jullien & Calvet, 1903: 139, pl. 16, figs 5a, 5b [3].

*Schizoporella fayalensis* Calvet: Calvet, 1907: 420 [6]; d’Hondt, 1975: 576 [18].

*Schizomavella fayalensis* (Calvet): Calvet, 1931: 81 [7].

*Schizoporella obsoleta* (Jullien): d’Hondt, 1975: 575 (in part) [18].

*Type material*: Lectotype (here designated): MNHN IB-2009-1553 (former part of MNHN 5939), colony on bivalve fragment, *Hirondelle*, Stn 226, 38°31.19'N 28°34.61'W, Pico-Faial Channel (Azores), 14/08/1888, 130 m, Calvet Coll.

Paralectotypes (here designated; all samples with the same information as lectotype): MNHN 5939, several colonies on shells; MOM INV-22500, -22604, -22605, -22606, -22607, -22608, -22609, -22610, -22611, -22612, -22613, -22614, dry, mounted on slide, one colony fragment in each. MOM INV-22499, three specimens with colonies of variable size on bivalves and a serpulid, occasionally growing free beyond substratum, stored in ethanol.

*Other material examined*: See S2 Text.

*Description*: Colony loosely encrusting, unilaminar or multilaminar, forming broad irregular crusts; or free-living, cupuliform.

Autozooids in regular radial series, or irregularly arranged in successive layers.

Autozooids oval to polygonal, or even subrectangular; separated by fine raised sutures; transverse distal wall often curved. Vertical walls with small uniporous septula arranged in rows parallel to basal wall.

Frontal shield very slightly convex with a finely granular surface, evenly perforated by up to 60 circular pseudopores; marginal areolar pores frequently elongate and larger than frontal pores. Periorbital region somewhat swelled, marked with small nodules or ridges.

Primary orifice transversely elliptical, wider than long, its distal edge projecting distally; poster almost entirely occupied by a wide, shallow sinus with rounded and gently sloping shoulders; condyles conspicuous, subrectangular, distinctly offset from and extending parallel to the orifice margin, slightly serrated only in their inner half and tip. Oral spines absent.

One distolateral avicularium, sometimes lacking, placed near zooid margin at right or left, occasionally level with the orifice, normally with its distal edge proximal of the sinus. Avicularium narrow, elliptical, twice as long as wide, with complete crossbar and an extensive foramen occupying a half of the rostrum, distal shelf broad and sloping towards foramen. Rostrum semi-elliptical, proximolaterally directed, with the lateral edge usually in contact with the lateral zooidal suture.

Ovicell hyperstomial, becoming subimmersed owing to secondary calcification during ontogeny, globular, relatively large, frontally flattened,. Exposed ectooecium a wide, flat, semicircular area with rounded or elongate pseudopores that are larger at the periphery; secondary calcification of distal zooid forming a crown of prominent conical nodules completely encircling the exposed ectooecial area, showing a fine proximomedian suture; secondary calcification may occasionally even cover the entire pseudoporous area with a thin layer but without occluding the pseudopores. Primary orifice of ovicellate zooids appearing as slightly more circular and larger than non-ovicelled zooids, and with a shallower and broader sinus. Opening of ovicell inclined at an angle, formed by the concave proximal margin of the ooecium extending to the proximolateral corners of the orifice. Ovicell closure of the subcleithral type.

An ancestrula was not observed.

*Remarks*: *Schizoporella fayalensis* was described by Calvet [3] based on numerous specimens collected by the *Hirondelle* cruise at 130 m depth in the Pico-Faial Channel (Azores). Most of these specimens are preserved at the MOM while one sample, consisting of several colonies preserved in ethanol, is at the MNHN (5939). We have selected one colony of the latter sample as the lectotype of the species, which received a new collection number (MNHN IB-2009-1553); the remaining colonies of MNHN 5939, as well as the 13 specimens at the MOM, are designated as paralectotypes. As several colonies were initially present, listing of sample MNHN 5939 as holotype of *S*. *fayalensis* by Tricart & d'Hondt [42] is erroneous.

The species was subsequently reported by Calvet [6,7] from the same area, between 80 and 115 m depth, and later by d’Hondt [18] near São Miguel Island (Azores), between 120 and 258 m depth.

While the overall suite of characters allows placing *S*. *fayalensis* in the lanceoporid genus *Stephanotheca* Reverter-Gil et al., 2012, some of the morphological characters of the species necessitate a minor amendment of the generic diagnosis of *Stephanotheca*. As none of the ovicells were observed to be closed by the operculum, presumably because they were empty and the opercula were resting on the primary orifice rim, the ovicell closure type is subcleithral rather than cleithral [51], at least in some species of the genus. Moreover, the avicularium must not necessarily be suboral in *Stephanotheca* but may be positioned anywhere in proximity of the orifice.

Most of the *Stephanotheca* species recorded so far are from the Mediterranean Sea. Up to now, only *Stephanotheca ochracea* (Hincks, 1862) had an Atlantic distribution. *Stephanotheca fayalensis* is easily distinguished from all other species of the genus by its distolateral avicularium.

Most of the material of *S*. *fayalensis* is loosely encrusting. For instance, the sample MNHN 3769 contains three fragments that were probably once joined in a single, large, multilaminar 3D-construction growing on and over bivalves and serpulids. The construction is strengthened by frontal budding and/or multilaminar self-overgrowth whereas its unilaminar colony margins grow free from the substratum and are able to bridge gaps. Interestingly, *S*. *fayalensis* is the only species of the genus that may also form free-living, cupuliform colonies (Fig 20). Some Australian species of *Calyptotheca* Harmer, 1957, a genus closely related to *Stephanotheca*, may also develop conical, free living colonies [52,53]; in these species, the ancestrula settles on a small substratum, like a pebble or shell fragment, and the colony grows overhanging the object. The four colonies of *S*. *fayalensis* in the sample MNHN 7547, originally reported by d’Hondt [18] as *S*. *obsoleta*, are free, cupuliform colonies (Fig 20); autozooids, avicularia and ovicells in these colonies fit the present description of the species, although frontal pores are smaller than those of the lectotype, and avicularia are frequently lacking. None of the colonies show any remainder of a substrate like sand grains, small pebbles or shells; moreover, there is no evidence of rootlets or other form of attachment. These colonies grow by peripheral budding of uni- to multilaminar colony fragments that were previously growing free of a substratum (Fig 20). Fracturing of the colonies takes place, and is facilitated, along the lateral zooidal walls, and any stronger contact may inflict breakage. Damage of the free colony edge can be caused by mobile benthic organisms, by wave action, or by remobilisation of coarse sediment during a storm. As the wave base of strong storms is deeper than 100 m in the Azores [54], at least the shallower populations of *S*. *fayalensis*, which are recorded from around 80 m, are thus occasionally in reach of a strong swell.

The convex colony shape of the free-living colony is presumably produced because the original curvature of the fragment is maintained by the newly formed peripheral buds. In all four free colonies the colony surface of the initial fragment is already convex, none has formed from a fragment with a concave curvature. Although there is also apical budding forming new layers that strengthen the entire free-living colony (Fig 19F), basal thickening was not observed, and the discoidal colonies are quite fragile. As a result, fragments of these colonies are likely to form new colonies as well.

This discoidal morphology is similar to many other unrelated free-living forms from around the world and in the geological past (A. O’Dea, personal communication). This process obviously represents a type of asexual multiplication by means of fragmentation in unstable environments; however, the process described here for *S*. *fayalensis* has nothing to do with genetically predetermined autofragmentation as described in cupuladriids (e.g., [55]).

## Concluding Remarks

The diversity of the genus *Schizomavella* is still poorly known, as demonstrated by the description of new species in recent years, in the NE Atlantic and in the Mediterranean [9,10,11,12,13, present paper]. The generic position of some species has been a matter of debate, raising the question of generic circumscription. Gordon & d’Hondt [32] started dismantling the genus *Schizomavella* by erecting a new genus, *Parkermavella*, for species with only marginal areolae. Reverter Gil et al. [17] erected *Stephanotheca* for species with cleithral ovicells. Some other Indo-Pacific species were transferred to the genus *Calyptotheca* [56]. However, there are still some differences between the remaining species of the genus that allow splitting them into two groups. As some of the characters are partly overlapping, however, we have decided to establish two new taxa at subgeneric level (see Fig 1).

**Fig 22:**
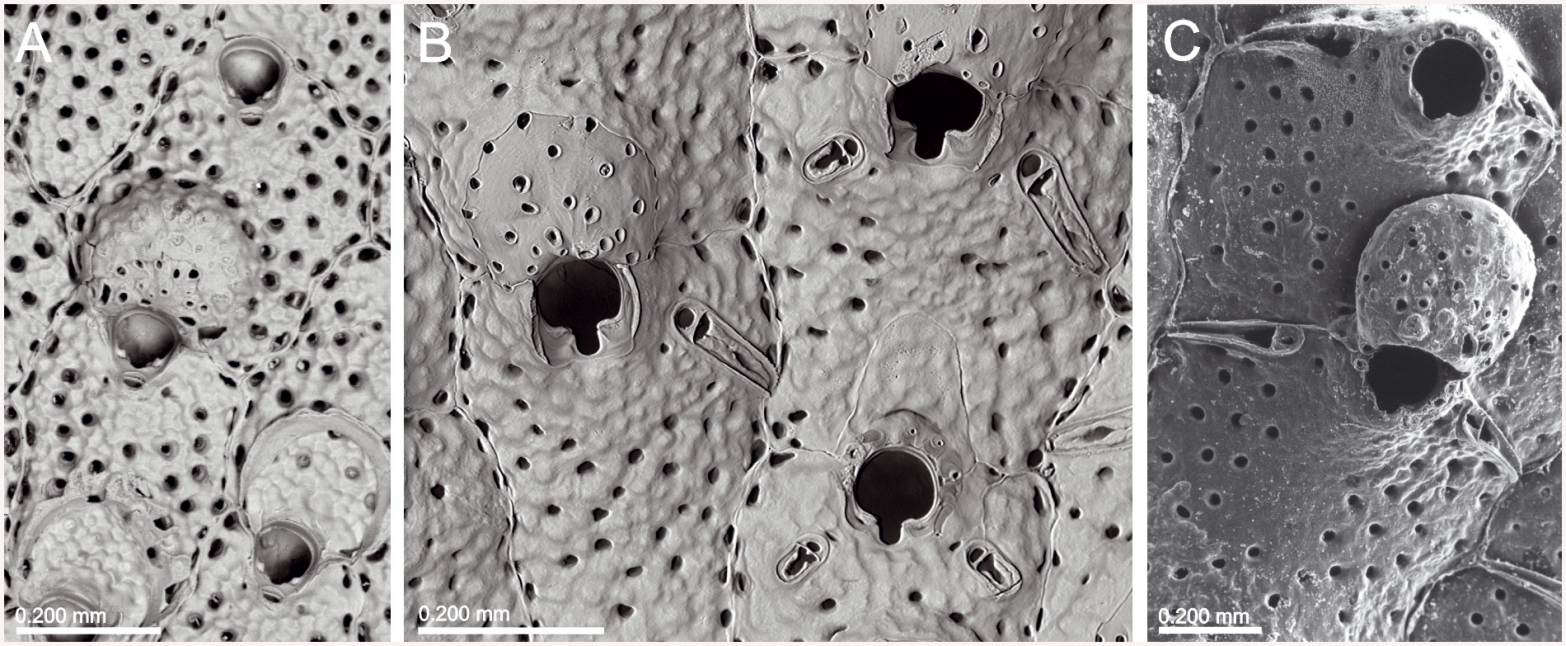
Representatives of the morphotaxa introduced here. (A) *Schizomavella* (*Schizomavella*) *auriculata* (Galicia, NW Spain), a typical *Schizomavella*; note the single suboral avicularium, the lack of spines, the extensive frontal calcification, and the reduced area of exposed ectooecium. (B) *Schizomavella* (*Calvetomavella*) *discoidea* (NHMUK 1911.10.1.1088); a species showing transitional features such as smaller avicularia with rounded rostra that are positioned proximolaterally of the orifice as well as larger elongated ones, an ooecium that is only marginally calcified by secondary calcification of the distal zooid, and an intermediate number of oral spines. (C) *Schizomavella* (*Calvetomavella*) *phterocopa* (OLL 2015/6), a typical representative of the subgenus *Calvetomavella* with pointed avicularia, an ooecium that is not covered by secondary calcification, and numerous oral spines.

Among the species described in the present paper, three of them (*S*. *fischeri*, *S*. *richardi* and the newly introduced *S*. *rectangularis*) are assigned to the subgenus *Schizomavella*. Most of the oft-cited species from the European continental shelf and oceanic islands, such as *S*. *auriculata* (Fig 22A) or *S*. *linearis*, also belong to this subgenus. Typically, these species occur in shallow-water, although there are notable exceptions (e.g. *S*. *fischeri* is reported from depths of 1070 m). Another species, *S*. *fayalensis*, is placed in the superficially similar genus *Stephanotheca*, being the second Atlantic species of the genus and the first one in the Macaronesian region.

The remaining species treated here, together with *S*. *(C*.*) discoidea* and *S*. *(C*.*) noronhai*, form a clade within *Schizomavella* (Fig 1) for which we introduced the new subgenus *Calvetomavella*. In contrast to the subgenus *Schizomavella*, most of these species are predominantly bathyal.

Although the two groups defined here as subgenera are clearly separated, the phylogenetic analysis based on 33 morphological characters did not resolve the relationships between all the species within each subgenus. This is particularly true for the subgenus *Schizomavella* in which the relationships between most species is unresolved. We explain this deficiency by the use of only a very limited number of species included in the analysis and the existence of an elevated number of polymorphic characters. This problem was already encountered in phylogenetic analyses of the bryozoan genera *Micropora* [57] and *Licornia* [58]

Within the subgenus *Calvetomavella*, *Schizomavella (C*.*) neptuni* and *S*. *(C*.*) noronhai* seem to be sister and sibling species, although the support is not overly strong (Fig 1). They differ from the other species in the subgenus by the small, oval, suborally placed avicularia. While *S*. *(C*.*) neptuni* seems to be widely distributed in deep waters from the North of the Bay of Biscay to the western Mediterranean but is absent from Madeira, *S*. *(C*.*) noronhai* only occurs at an unknown depth in this island.

*Schizomavella (C*.*) phterocopa* n. sp. and *S*. *(C*.*) paucimandibulata* also seem to be closely allied (Fig 1), both species having enlarged triangular avicularia aligned parallel to the distal zooidal margin, which may frequently be asymmetrically developed, a large number of frontal shield perforations, and the ectooecium is perforated by rimmed pseudopores of irregular size. Moreover, both species are known from the central North Atlantic region. Another Azorean species, *S*. *(C*.*) triaviculata*, also has distal enlarged triangular avicularia. In contrast to the cladistic analysis, however, in which it occurs at the base of the two crown groups (Fig 1), its overall optical impression and geographic occurrence suggests that it may be as part of the crown group containing *S*. *(C*.*) paucimandibulata* and *S*. *(C*.*) phterocopa*.

*Schizomavella (C*.*) triaviculata* differs from all the other species of the subgenus by the tendency to form additional small frontal avicularia. One (or occasionally a pair) is placed suborally, another one or two are situated on the peristome of ovicellate zooids. Moreover, enlarged avicularia growing elsewhere on the frontal shield and along the lateral interzooidal sutures in areas of the colony where zooid growth is disordered. This last type of avicularium may be also present in some populations of *Schizomavella (C*.*) phterocopa* n. sp.

*Schizomavella (C*.*) discoidea* is likely to represent a link between both groups of species (Figs 2 and 22B). It was originally described from Madeira, whereas other records from the European continental shelf probably comprise at least one undescribed species, so at present *S*. *discoidea* must be regarded as a species complex. On the one hand, the avicularia of *S*. *(C*.*) discoidea s*.*l*. are typically long and placed parallel to the distal zooidal margin (though slightly proximal to it), like in *S*. *(C*.*) phterocopa* n. sp. and *S*. *(C*.*) paucimandibulata*; moreover, ovicellate zooids of *S*. *(C*.*) discoidea* lack spines and develop a thin peristome, like in *S*. *(C*.*) triaviculata*. On the other hand, the avicularia are not pointed, and sometimes they can be small, oval, and even placed laterally suborally, like in *S*. *(C*.*) neptuni* and *S*. *(C*.*) noronhai*.

**Fig 23:**
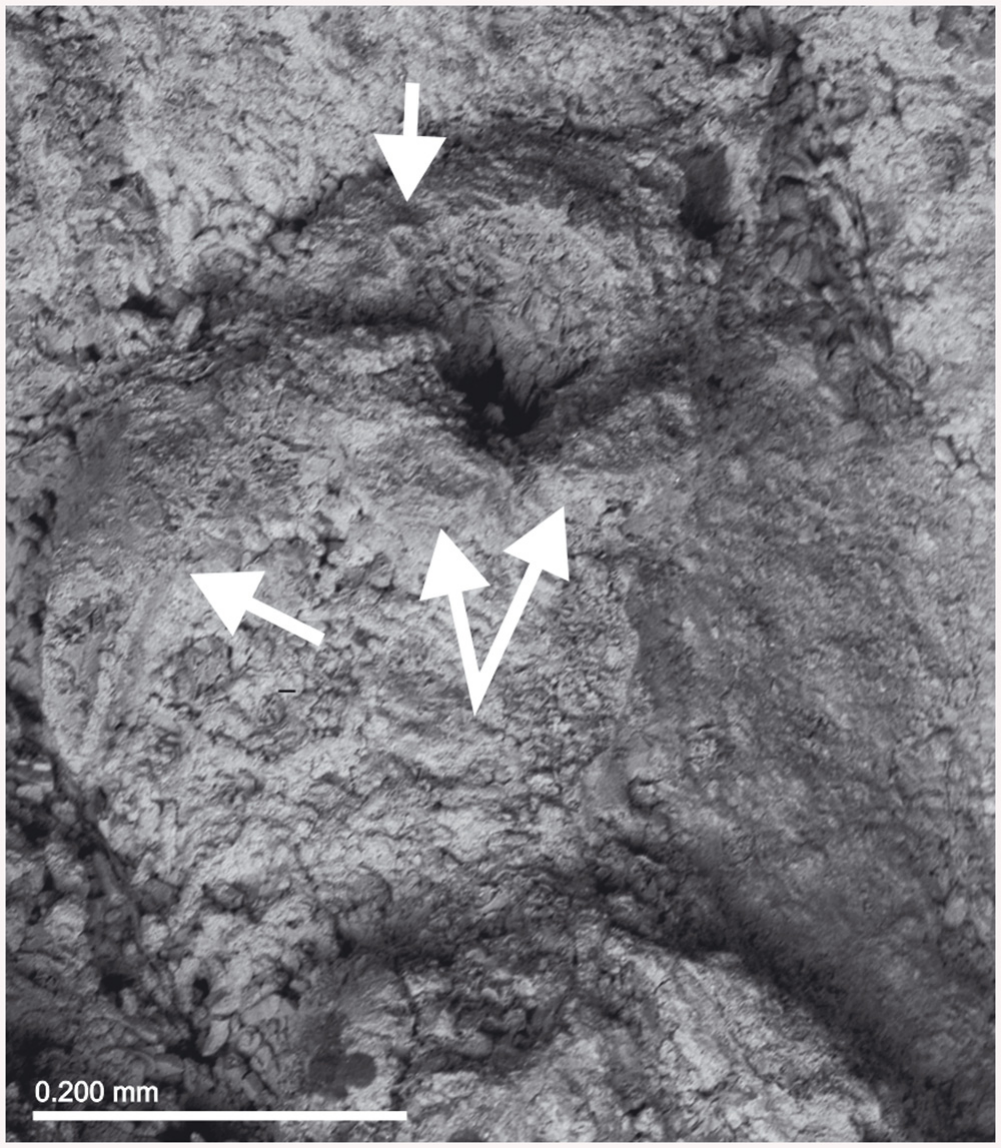
An early Pliocene fossil from Santa Maria Island (Pedra-que-pica outcrop) resembling the Recent *S*. *(C*.*) triaviculata* var. *multimandibulata*. Note the elongated distal avicularium (left arrow), the pair of suboral avicularia (central arrows), and the oral spines (top arrow).

Owing to the evolution of distinct morphological characters, and the widespread occurrence of deep-water *Calvetomavella* species in the NE Atlantic continental shelf and oceanic islands, the separation of the two groups can be expected to reach well into the Neogene. This notion is supported by the presence of an early Pliocene fossil from Santa Maria Island (Azores) [59] that closely resembles the modern *S*. *(C*.*) triaviculata s*.*l*. (Fig 23), or to be more precise, the specimens recorded as *S*. *triaviculata* var. *multimandibulata* from just north of Santa Maria by d’Hondt [18]. Although poorly preserved, the main characteristic features (a perforated frontal shield, the presence of some six spines, elongated distal avicularia, plus a pair of short suboral avicularia pointing towards each other) can be observed.

Sampling of fresh material from the Mediterranean Sea as well as from the NE Atlantic continental shelf and oceanic islands and seamounts will be essential for future work on the evolution, diversity, biogeography and ecology of the genus *Schizomavella*. As the present study shows, morphological characters are insufficient to ascertain the systematic relationships of its species, and few fossil taxa exist in order to reconstruct their evolutionary history. Genetic studies will thus prove to be indispensable.

## Supporting Information

S1 Table Matrix of 33 characters used in the phylogenetic analysis. (DOCX)

S1 Text List of characters included in the phylogenetic analysis. (DOCX)

S2 Text List of additional, non-type material examined. (DOCX)
