# Supplementary material for: Diversity and Systematics of Schizomavella Species (Bryozoa: Bitectiporidae) from the Bathyal NE Atlantic
Source: PLoS One. 2015 Oct 21;10(10):e0139084. doi: 10.1371/journal.pone.0139084 (PMC4619517; doi:10.1371/journal.pone.0139084)
Supplement: S1 Table — (DOCX) [file pone.0139084.s001.docx]

| Spp. | C1 | C2 | C3 | C4 | C5 | C6 | C7 | C8 | C9 | C10 | C11 | C12 | C13 | C14 | C15 | C16 | C17 | C18 | C19 | C20 | C21 | C22 | C23 | C24 | C25 | C26 | C27 | C28 | C29 | C30 | C31 | C32 | C33 |
| --- | --- | --- | --- | --- | --- | --- | --- | --- | --- | --- | --- | --- | --- | --- | --- | --- | --- | --- | --- | --- | --- | --- | --- | --- | --- | --- | --- | --- | --- | --- | --- | --- | --- |
| Schiz. neptuni | 0 | 0 | 0 | 1 | 3 | 1 | 1 | 0 | 1 | 3 | 0 | 2 | 0 | 0 | 0 | 1 | 0 | - | 2 | 0 | 0 | 0 | - | 0 | 0 | 0 | 0 | 1 | 0 | 0 | 0 | 0 | 3 |
| Schiz. noronhai | 0 | 1 | 0 | 0 | 1 | 3 | 1 | 0 | 1 | 3 | 0 | 2 | 0 | 0 | 0 | 1 | 0 | - | 2 | 0 | 0 | 0 | - | 0 | 0 | 0 | 0 | 1 | 0 | 0 | 0 | 0 | 4 |
| Schiz. triaviculata | 0 | 2 | 0 | 1 | 2 | 4 | 1 | 0 | 1 | 2 | 2 | 0 | 1 | 1 | 1 | 1 | 2 | 0 | 2 | 1 | 1 | 1 | 1 | 1 | 1 | 0 | 0 | 1 | 0 | 0 | 0 | 0 | 6 |
| Schiz. paucimandibulata | 0 | 0 | 1 | 1 | 4 | 1 | 1 | 0 | 1 | 2 | 0 | 2 | 0 | 2 | 2 | 2 | 0 | - | 2 | 1 | 1 | 1 | - | 0 | 0 | 0 | 0 | 2 | 0 | 0 | 0 | 0 | 5 |
| Schiz. phterocopa | 0 | 1 | 2 | 0 | 1 | 4 | 1 | 0 | 1 | 3 | 0 | 2 | 0 | 1 | 2 | 2 | 0 | - | 2 | 10 | 1 | 1 | - | 0 | 1 | 0 | 0 | 2 | 0 | 0 | 0 | 0 | 5 |
| Schiz. discoidea | 0 | 0 | 0 | 1 | 2 | 1 | 1 | 0 | 0 | 2 | 2 | 0 | 1 | 0 | 3 | 0 | 0 | - | 2 | 10 | 0 | 0 | - | 0 | 0 | 1 | 0 | 1 | 0 | 0 | 0 | 0 | 1 |
| Schiz. richardi | 0 | 1 | 2 | 0 | 12 | 13 | 2 | 1 | 0 | 3 | 0 | 1 | 0 | 1 | 3 | 1 | 1 | 0 | 0 | - | - | - | 0 | 0 | 0 | 0 | 1 | 1 | 1 | 1 | 0 | 0 | 1 |
| Schiz. fischeri | 0 | 1 | 2 | 1 | 12 | 3 | 1 | 1 | 0 | 2 | 0 | 1 | 0 | 0 | 1 | 1 | 1 | 0 | 0 | - | - | - | 1 | 0 | 0 | 0 | 1 | 0 | 0 | 1 | 0 | 0 | 5 |
| Schiz. auriculata | 1 | 1 | 2 | 2 | 12 | 0 | 10 | 1 | 0 | 1 | 1 | 0 | 0 | 0 | 3 | 10 | 1 | 0 | 0 | - | - | - | 0 | 0 | 0 | 0 | 2 | 12 | 0 | 1 | 0 | 0 | 1 |
| Schiz. linearis | 0 | 1 | 2 | 1 | 2 | 3 | 0 | 0 | 0 | 1 | 1 | 0 | 0 | 0 | 0 | 10 | 0 | - | 2 | 0 | 0 | 1 | - | 1 | 0 | 1 | 2 | 1 | 1 | 1 | 0 | 0 | 1 |
| Schiz. cornuta | 2 | 1 | 2 | 0 | 2 | 1 | 2 | 0 | 0 | 1 | 1 | 0 | 0 | 0 | 3 | 0 | 1 | 10 | 0 | - | - | - | 0 | 0 | 0 | 1 | 2 | 10 | 1 | 1 | 0 | 0 | 1 |
| Schiz. sarniensis | 0 | 1 | 2 | 2 | 1 | 2 | 12 | 1 | 0 | 1 | 1 | 0 | 0 | 0 | 4 | 0 | 1 | 10 | 0 | - | - | - | 0 | 0 | 0 | 1 | 0 | 0 | 1 | 3 | 1 | 0 | 1 |
| Schiz. halimedae | 3 | 2 | 1 | 0 | 4 | 3 | 0 | 1 | 0 | 2 | 2 | 0 | 1 | 0 | 3 | 1 | 1 | 10 | 0 | - | - | - | 0 | 0 | 0 | 1 | 2 | 12 | 0 | 2 | 0 | 0 | 0 |
| Steph. fayalensis | 4 | 1 | 2 | 0 | 0 | 2 | 0 | 0 | 0 | 0 | - | 0 | 0 | 2 | 3 | 0 | 0 | - | 1 | 0 | 0 | 0 | - | 0 | 0 | 0 | 1 | 0 | 0 | 1 | 0 | 1 | 3 |
| Hippo. teresae | 0 | 1 | 2 | 0 | 0 | 1 | 0 | 1 | 0 | 0 | - | 0 | 0 | 1 | 4 | 0 | 0 | - | 1 | 1 | 0 | 0 | - | 0 | 0 | 0 | 0 | 1 | 1 | 3 | 1 | 2 | 2 |

**Supporting Information S1 Table.** Matrix of characters used in the phylogenetic analyses.
