# Supplementary material for: Diversity and Systematics of Schizomavella Species (Bryozoa: Bitectiporidae) from the Bathyal NE Atlantic
Source: PLoS One. 2015 Oct 21;10(10):e0139084. doi: 10.1371/journal.pone.0139084 (PMC4619517; doi:10.1371/journal.pone.0139084)
Supplement: S1 Text — (DOCX) [file pone.0139084.s002.docx]

**Supporting Information S1 Text.** List of characters used for the phylogenetic analysis (the characters are treated in the description and remarks of each species).

C1. Orifice morphology

0 Orbicular

1 Drop shaped

2 Horseshoe

3 D-shaped

4 Transversely elliptical

C2. Sinus size

0 Longer than wide

1 Wider than long

2 As long as wide

C3. Sinus shape

0 Square U-shaped

1 Drop-shaped

2 Open U-shaped

C4. Proximolateral orifice margins

0 Sloping towards the sinus

1 Straight

2 Upturned

C5. Sinus width in relation to total width of the proximal orifice margin

0 ~1

1 ½

2 1/3

3 ¼

4 <1/5

C6 . Condyle tips

0 Free, pointed

1 Free, blunt and smooth

2 Free, blunt and serrated

3 Tapered towards proximolateral orifice margin, surface smooth

4 Tapered towards proximolateral orifice margin, surface bluntly denticulated

C7. Condyle size

0 Not reaching the margin of the sinus

1 Reaching the margin of the sinus

2 Extending beyond the margin of the sinus

C8. Periorbital rim of gymnocystal calcification

0 Absent

1 Present

C9. Periorbital region projecting above zooidal surface

0 Absent

1 Present

C10. Mean number of spines in early ontogenetic zooids

0 No spines

1 One to three

2 Five to seven

3 Eight or more

C11. Persistence of spines during ontogeny

0 In all zooids

1 Lost in late ontogenetic zooids

2 Persisting only in infertile zooids

C12. Number of spines in ovicellate zooids

0 None

1 Two

2 Four

C13. Peristome

0 Absent

1 Present

C14. Mean number of frontal shield pores

0 <20

1 20-30

2 >30

C15. Frontal shield surface

0 Dimpled

1 Smooth

2 Wrinkled

3 Granular/nodular

4 Spiky

C16. Secondary frontal calcification

0 Frontal calcification (sub) immersing the ovicell

1 Frontal calcification covering the ovicell without immersing it

2 Secondary calcification absent or very reduced

C17. Maximum number of suboral avicularia per zooid

0 Absent

1 One

2 Two

C18. Size of suboral avicularia

0 Small

1 Large

C19. Maximum number of lateral avicularia per zooid

0 Absent

1 One

2 Two

C20. Size of lateral avicularia

0 Small

1 Large

C21. Position of lateral avicularia

0 Distal suboral

1 Distal marginal

C22. Rostrum of lateral avicularia

0 Rounded

1 Pointed

C23. Rostrum of suboral avicularia

0 Rounded

1 Pointed

C24. Vicarious avicularia

0 Absent

1 Present

C25. Additional marginal avicularia

0 Absent

1 Present

C26. Dimorphic avicularia

0 Absent

1 Present

C27. Position of ectooecial pores

0 Uniform

1 Central

2 Proximal

C28. Demarcation of ectooecial pores

0 Absent

1 Low rim

2 Funnel-shaped rim

C29. Ooecial pore shape

0 Circular

1 Irregular

C30. Secondary calcification on the ovicell

0 Basal peripheral

1 Marginal

2 Distal half

3 Extensive

C31. Perforation of secondary calcification covering the ovicell

0 Absent

1 Present

C32. Type of ovicell closure

0 Acleithral

1 Subcleithral

2 Cleithral

C33. Mean autozooid size, in µm^2^ (mean zooid length x mean zooid width)

0 <120

1 140-180

2 210-220

3 280-310

4 360-370

5 470-500

6 >550
