# Supplementary material for: Diversity and Systematics of Schizomavella Species (Bryozoa: Bitectiporidae) from the Bathyal NE Atlantic
Source: PLoS One. 2015 Oct 21;10(10):e0139084. doi: 10.1371/journal.pone.0139084 (PMC4619517; doi:10.1371/journal.pone.0139084)
Supplement: S2 Text — (DOCX) [file pone.0139084.s003.docx]

**Supporting Information S2 Text.** List of additional, non-type material examined.

***Schizomavella (Schizomavella) fischeri*** (Jullien, 1882)

As *S. fischeri*:

MNHN 6957: *Thalassa* Stn T466, 43°36.3'N 09°04.8'W, off NW Iberian Peninsula, 798-802 m, d’Hondt Coll.

MNHN 6998: *Thalassa* Stn X343, 44º07.0'N-44º07.8'N 04º38.8'W-04º38.9'W, off N Iberian Peninsula, 600-655 m, d’Hondt Coll.

MNHN 6999: same data as MNHN 6998.

MNHN 7009: *Thalassa* Stn X350, 44º04.2'N 04º46.0'W, off N Iberian Peninsula, 503-507 m, d’Hondt Coll.

MNHN 7096: *Thalassa* Stn W422, 44º03.6'N-44º04,1'N 06º58.7'W-06º59.1'W, off NW Iberian Peninsula, 700-850 m, d’Hondt Coll.

MNHN 7101: *Thalassa* Stn W423, 44°03.7'N 07°07.5'W, off NW Iberian Peninsula, 710-1070 m, d’Hondt Coll.

MNHN 7105: *Thalassa* Stn W424, 44°02.2'N 07°08.9'W, off NW Iberian Peninsula, 300-700 m, d’Hondt Coll.

MNHN 7186: *Thalassa* Stn Y394, 41º18.6'N 09º13.8'W, off N Portugal, 410 m, d’Hondt Coll. (Examined using SEM)

MNHN 7263: *Thalassa* Stn Y442, 44°12.4'N 08°41.0'W, off NW Iberian Peninsula, 688 m, d’Hondt Coll.

MNHN 7612: *Thalassa* Stn T471, 43°40.0'N 08°57.2'W, off NW Iberian Peninsula, 562-574 m, d’Hondt Coll.

MNHN 7621: *Thalassa* Stn U852, 44°12.0'N 08°34.0'W, off NW Iberian Peninsula, 615-654 m, d’Hondt Coll.

MNHN 7631: *Thalassa* Stn W406, 43°55.5'N, 5°44.0'W, off N Iberian Peninsula, 700-400 m, d’Hondt Coll.

MNHN 7637: same data as MNHN 7631.

MNHN 7647: *Thalassa* Stn Y428: 44º11.8'N 08º40.6'W, off NW Iberian Peninsula, 500 m, d’Hondt Coll.

MNHN 7648: same data as MNHN 7647.

MNHN 7653: *Thalassa* Y440, 44°12.2'N 08°40.2'W, off NW Iberian Peninsula, 490 m, d’Hondt Coll. (Examined using SEM)

MNHN IB-2013-564: *Thalassa* Stn Y433, 44°12.0'N 08°40.5'W, off NW Iberian Peninsula, 605-620 m. 04/08/1972. Harmelin Coll. (Examined using SEM)

MNHN IB-2013-565: *Thalassa* Stn Y434, 44°12.0'N 08°40.8'W, off NW Iberian Peninsula, 605-620 m. 04/08/1972. Harmelin Coll. (Examined using SEM)

MNHN IB-2013-566: *Thalassa* Stn U807, 44°11.0'N 08°40.8'W, off NW Iberian Peninsula, 450-500 m. 18/10/1968. Harmelin Coll. (Examined using SEM)

MNHN IB-2013-567: *Noroit Seamount 1* Stn DW106, 42°41.6'N 11°48.5'W, Galicia Bank, 765 m. 18/10/1987. Harmelin Coll. (Examined using SEM)

MNHN IB-2013-568: *Noroit Seamount 1* Stn DW111, 42°39.9'N 11°35.8'W, Galicia Bank, 675-685 m. 19/10/1987. Harmelin Coll. (Examined using SEM)

MNHN IB-2013-569: *Faial* Stn SME 1192 – P.59, 38º16.8'N 08º56.4'W, Setubal Canyon, off central Portugal, 250-300 m. Harmelin Coll.

MNHN IB-2013-570: Gesite DR 72, 35°44.3'N 13°05.1'E, Linosa Trough between Malta and Tunis, 320-600 m. 1973. Harmelin Coll. (Examined using SEM)

*BANGAL* 2011 DR01, 42º53.2'N, 11º39.7'W, Galicia Bank, 20/07/2011, 1414 m.

*BANGAL* 2011 DR08, 42º31.6'N, 11º43.3'W, Galicia Bank, 25/07/2011, 1138 m.

*BANGAL* 2011 DR10, 42º45.9'N, 11º49.1'W, Galicia Bank, 26/07/2011, 826 m.

*BANGAL* 2011 DR09, 42º45.3'N, 11º46.3'W, Galicia Bank, 26/07/2011, 779 m. (Examined using SEM)

*BANGAL* 2011 V01, 42º47.3'N, 11º48.1'W, Galicia Bank, 26/07/2011, 867 m. (Examined using SEM)

MB37-000038: *Poseidon* Stn 2, 41°09.3'N 09°20'W, off N Portugal, 800-900 m, 20/11/1984. (Examined using SEM)

As *S. obsoleta*:

MNHN 2348: same data as holotype of *S. obsoleta*, Calvet Coll.

***Schizomavella (Schizomavella) richardi*** (Calvet *in* Jullien & Calvet, 1903)

MNHN 1391: *Talisman* 1883, Stn 125, 38°29.1'N 28°37.33'W [estimate], Pico-Faial Channel (Azores), 13/08/1883, 80-115 m, one colony on bivalve occurring together with *Membranipora filum* Jullien, *Micropora coriacea* (Esper) and *Hippothoa flagellum* Manzoni, Calvet Coll. (Examined using SEM)

MNHN 2436: same data as MNHN 1391, one colony on bivalve shell fragment, not in the original label, occurring together with *Lepralia canalifera* (Busk), *Hippothoa nebulosa* Jullien and *Cribrilina figularis* (Johnston). (Examined using SEM)

MNHN 4009: same data as MNHN 1391, one colony on bivalve shell fragment, not in the original label, occurring together with *Membraniporella alice* Jullien.

MNHN 4038: same data as MNHN 1391, one colony on bivalve shell fragment, not in the original label, occurring together with *Hippothoa nebulosa* Jullien. (Examined using SEM)

MNHN 7555: *Biaçores* Stn 208, 37º16'N 24º47'W, Azores, 07/11/1971, 155-290 m, d’Hondt Coll. (Examined using SEM)

MNHN IB-2013-571: *Jean Charcot Biaçores* Stn 11, 38°30'N 27°14.5'W, off S Terceira (Azores), 76-105 m. Harmelin Coll.

OLL 2015/19, 38°30.55'N 28°36.61'W, off Faial (Azores), Sept. 2008, 150 m, a tiny colony fragment broken off a settlement panel, mounted on stub, sputter-coated, leg. M. Wisshak. (Examined using SEM)

Numerous unregistered colonies on PVC and limestone settlement panels, 38°30.55'N 28°36.61'W, off Faial (Azores), Sept. 2008, 150 m, leg. M. Wisshak (Senckenberg, Wilhelmshaven).

*Other material:*

*Schizomavella auriculata*: MOM INV-22504, 22620: *Hirondelle* Stn 58, 43º40'00''N 06º34'46''W, off N Iberian Peninsula, 07/08/1886, 134 m, two slides originally labelled as *Schizoporella richardi*, Calvet Coll. (Examined using SEM)

***Schizomavella (Calvetomavella) phterocopa* n. sp.**

**Additional material examined**

OLL 2011/405, *Meteor* 42/3, Stn 515, 13/09/1998, Great Meteor Bank, 29°48.9'N 28°29'W, 302 m, one colony on coral with *B.* *baderae*, *Hippothoa* sp. and Celleporidae indet., mounted on stub and sputter-coated.

OLL 2015/6, *Meteor* 42/3, Stn 519, 14/09/1998, Great Meteor Bank, 30°06.2'N 028°24.5'W, 416 m, one colony on coral, mounted on stub and sputter-coated. (Examined using SEM)

OLL 2015/7, *Meteor* 42/3, Stn 515, 13/09/1998, Great Meteor Bank, 29°48.9'N 28°29'W, 302 m, one colony on coral with *Hippothoa* sp., mounted on stub and sputter-coated. (Examined using SEM)

OLL 2015/8, *Meteor* 42/3, Stn 514, 13/09/1998, Great Meteor Bank, 29°57.7'N 28°21.7'W, 330 m, three small colonies (one on echinoid spine, two fragments free of substrate), mounted on stub and sputter-coated. (Examined using SEM)

OLL 2015/9, *Meteor* 42/3, Stn 465, 05/09/1998, Great Meteor Bank, 29°48.3'N 28°36.5'W, 302 m, one damaged colony on coral, mounted on stub and sputter-coated.

OLL 2015/10, *Meteor* 42/3, Stn 514, 13/09/1998, Great Meteor Bank, 29°57.7'N 28°21.7'W, 330 m, one colony on coral with *Bryopesanser* *baderae* Tilbrook, *Lagenipora* sp. and *Puellina* sp., mounted on stub and sputter-coated.

OLL 2015/11, *Meteor* 42/3, Stn 514, 13/09/1998, Great Meteor Bank, 29°57.7'N 28°21.7'W, 330 m, one colony on coral with *Buskea* sp., mounted on stub and sputter-coated.

OLL 2015/12, *Meteor* 42/3, Stn 515, 13/09/1998, Great Meteor Bank, 29°48.9'N 28°29'W, 302 m, four small colonies on coral with *Hippothoa* sp. and *Puellina* sp., mounted on stub and sputter-coated.

OLL 2015/13, *Meteor* 42/3, Stn 465, 05/09/1998, Great Meteor Bank, 29°48.3'N 28°36.5'W, 302 m, four small colonies on coral, dry.

OLL 2015/14, *Meteor* 42/3, Stn 514, 13/09/1998, Great Meteor Bank, 29°57.7'N 28°21.7'W, 330 m, three colonies on coral, dry.

OLL 2015/15, *Meteor* 42/3, Stn 515, 13/09/1998, Great Meteor Bank, 29°48.9'N 28°29'W, 302 m, eight colonies on coral and one free of substrate, with *Cribrilaria hexaspinosa* Harmelin and *Hippothoa* sp., dry.

OLL 2015/16, *Meteor* 42/3, Stn 565, 21/09/1998, Great Meteor Bank, 29°39.4'N 028°22.9'W, 403 m, two small colony fragments free of substrate, from sediment sample, dry.

OLL 2015/17, *Meteor* M9c, Stn 161a, June-July 1967, Great Meteor Bank, 29°53.2'N 28°32.5'W, 307 m, one colony on coral, dry.

OLL 2015/18, *Meteor* M19c-132, February 1970, Great Meteor Bank, 29°48.5'N 28°22.5'W, 392 m, on colony on coral with *B*. *baderae* and *Hippothoa* sp., dry.

MNHN IB-2013-572: *Meteor* 19-133/DD99, 29°47.9'N 28°22.1'W, Great Meteor Bank, 300 m, 18/02/1970. Two small colonies on coral. Harmelin Coll. (Examined using SEM)

MNHN IB-2013-573: *Suroit Seamount 2* Stn CP257, 34°04.50'N 30°15.00'W, Atlantis Seamount, 338 m. One small colony on a coral. Harmelin Coll. (Examined using SEM)

MNHN IB-2013-574: *Suroit Seamount 2* Stn DW274, 34°05.13'-34°04,96'N 30°13,57'-30°13,81'W, Atlantis Seamount, 280 m. One small colony on a bivalve. Harmelin Coll. (Examined using SEM)

***Schizomavella (Calvetomavella) triaviculata*** (Calvet *in* Jullien & Calvet, 1903)

MNHN 7457: “Jean-Charcot” *Biaçores*, Stn 79, 39º00'N 27º54'W, Azores, 16/10/71, 360-380 m, d’Hondt Coll. (Examined using SEM)

MNHN 7549: Holotype of *Schizoporella triaviculata* var. *multimandibulata* d’Hondt, 1975. “Jean-Charcot” *Biaçores*, Stn 207, 37º16.5'N 24º47'W, Formigas Islets (Azores), 7/11/71, 69-130 m, d’Hondt Coll. (labeled as var. *multiaviculata*; wrongly named as var. *plurimandibulata* by [42]). (Examined using SEM)

MNHN 7604: “Jean-Charcot” *Biaçores*, Stn 29, 38º08.6'N 28º57'W, Azores, 10/10/71, 360-375 m, d’Hondt. Coll.

MOM INV-22667: *Princesse Alice*, Stn 568, 37º54'N 25º35'25''W, Azores, 550 m, four colony fragments, Calvet Coll. (Examined using SEM)

MOM INV-22670: *Princesse Alice*, Stn 600, 38º30'35''N 28º16'20''W, Azores, 349 m, one colony, Calvet Coll. (Examined using SEM)

OLL 2015/20, Sept. 2008, off Faial (Azores), 38°30.546'N 28°36.606'W, 150 m, one colony fragment free of substrate, mounted on stub, sputter-coated, leg. M. Wisshak. (Examined using SEM)

Several unregistered colonies on PVC and limestone settlement panels, same data as for OLL 2015/20.

***Schizomavella (Calvetomavella) paucimandibulata*** (d’Hondt, 1975) **n. comb.**

MNHN 4021, one colony on bivalve shell fragment (not in the original label) [filed under, and occurring together with, *Cribrilina figularis* (Johnston)] (same locality information as MNHN 3771). (Examined using SEM)

MNHN 4296, one colony on base of *Reteporella atlantica* (Busk) (same locality information as MNHN 3771). (Examined using SEM)

MNHN IB-2013-575: *Président Théodore Tissier* Stn. 427, 37°40'N 25°30'W, off São Miguel (Azores), 300-320 m, 11/06/1951. Several colonies on a volcanic rock. Harmelin Coll.

MOM INV-22537: *Hirondelle* Stn 247, 38º24'N 28º01'26''W, Azores, 318 m, 30/VIII/1888, Calvet Coll. (Examined using SEM)

MOM INV-22669: *Princesse Alice*, Stn 597, 38º27'N 28º03'25''W, Azores, 523 m, two colonies on coral, Calvet Coll. (Examined using SEM)

MOM INV-22671: *Princesse Alice*, Stn 882, 38º03'40''N 28º34'45''W, Azores, 98 m, two colonies on *Reteporella* indet. and bivalve fragment, Calvet Coll. (Examined using SEM)

MOM INV-22668: *Princesse Alice*, Stn 584, 38º31'-38º30'30''N 26º49'15''-26º50'15''W, Azores, 845 m, one colony on coral, Calvet Coll. (Examined using SEM)

OLL 2015/21, Sept. 2008, off Faial (Azores), 38°30.546'N 28°36.606'W, 150 m, a tiny colony fragment detached from a settlement panel, sputter-coated, leg. M. Wisshak. (Examined using SEM)

OLL 2015/22, locality information same as OLL 2015/21, a tiny colony fragment detached from a settlement panel, mounted on stub, sputter-coated, leg. M. Wisshak. (Examined using SEM)

Unregistered specimen, locality information same as OLL 2015/21, one young colony encrusting a settlement panel, dry, leg. M. Wisshak (Senckenberg, Wilhelmshaven). (Examined using SEM)

Several unregistered colonies on PVC and limestone settlement panels, locality information same as OLL 2015/21.

***Schizomavella (Calvetomavella) neptuni*** (Jullien, 1882)

MNHN 1022: *Talisman*, 1883 Stn 10, 35º26'00''N 06º48'46''W, off Cap Spartel, strait of Gibraltar, 717 m, 10/06/1883, a colony on *Lophelia*, Calvet Coll. (Examined using SEM)

MNHN 2669: *Travailleur* Stn 40, 44°05'00''N 07°14'46''W, off NW Iberian Peninsula, 392 m, 15/08/1881, on *Reteporella* sp., Jullien Coll.

MNHN 4097: same data as MNHN 1022. (Examined using SEM)

MNHN 6915: *Thalassa* T478, 44°09.9'N 08°45.9'W, off NW Iberian Peninsula, 513-550 m.

MNHN 6983: *Thalassa* Stn X313, 44°02.6’N 04°55.2’W, off N Iberian Peninsula, 580-525 m, d’Hondt Coll.

MNHN 6988: *Thalassa* X325, 44º06.8'N-44º07.0'N 04º45.5'W, off N Iberian Peninsula, 660-800 m, d’Hondt Coll.

MNHN 6990: same data as 6988, one small colony on a shell fragment.

MNHN 6998: *Thalassa* Stn X343, 44°07.0’N 04°38.8’W, off N Iberian Peninsula, 600-655 m, d’Hondt Coll.

MNHN 6999: one small colony, same data as MNHN 6998.

MNHN 7003: *Thalassa* Stn X344, 44°06.7’N 04°41.0’W, off N Iberian Peninsula, 560-530 m, d’Hondt Coll.

MNHN 7021: *Thalassa* X359, 44º07.2'N 04º49.4'-04º49.6'W, off N Iberian Peninsula, 605-630 m, d’Hondt Coll.

MNHN 7027: *Thalassa* X362, 44º06.5'N-44º06.8'N 04º50.8'W-04º50.9'W, off N Iberian Peninsula, 585-600 m, d’Hondt Coll.

MNHN 7028: *Thalassa* X361, 44º06.0'N 04º50.1'W-04º50.2'W, off N Iberian Peninsula, 582-595 m, d’Hondt Coll.

MNHN 7032: *Thalassa* X374, 44º07.2'N 04º42.7'W-04º43.0'W, off N Iberian Peninsula, 570-582 m, d’Hondt Coll.

MNHN 7064: *Thalassa* W393, 44º06.9'N 04º44.1'W, off N Iberian Peninsula, 590-970 m, two small colonies, d’Hondt Coll.

MNHN 7077: *Thalassa* W405, 43º56.5'N 05º44.0'W, off N Iberian Peninsula, 400-690 m. several small colonies on a rock, d’Hondt Coll.

MNHN 7111: *Thalassa* W429, 44°10.5'N 08°41.5'W, off NW Iberian Peninsula, 430-450 m, d’Hondt Coll.

MNHN 7122: *Thalassa* W434, 44°11.8'N 08°40.4'W, off NW Iberian Peninsula, 500-540 m, one colony on a rock, d’Hondt Coll.

MNHN 7123: same data as 7122.

MNHN 7125: *Thalassa* W433, 44°10.2'N 08°41.5'W, off NW Iberian Peninsula, 435-460 m, one colony on a rock, d’Hondt Coll.

MNHN 7133: *Thalassa* W444, 44°10.1'N 08°38.4'-38.5'W, off the NW Iberian Peninsula, 452-580 m, d’Hondt Coll.

MNHN 7156: *Thalassa* W392, 44º06.9'N 04º49.3'W, off N Iberian Peninsula, 600-1130 m, one colony on a rock, d’Hondt Coll.

MNHN 7160: *Thalassa* Stn W388, 44°04.5’N 04°28.8’W, off N Iberian Peninsula, 750-850 m, d’Hondt Coll.

MNHN 7163: *Thalassa* W390, 44º05.0'N 04º31.0'W, off N Iberian Peninsula, 760-1000 m, one colony on a rock, d’Hondt Coll.

MNHN 7222: *Thalassa* Y428, 44°11.8'N 08°40.6'W, off NW Iberian Peninsula, 500 m.

MNHN 7227: same data as MNHN 7222.

MNHN 7240: *Thalassa* Y434, 44°12.0'N 08°40.8'W, off NW Iberian Peninsula, 620 m.

MNHN 7269: *Thalassa* U807, 44°11.0'N 08°40.2'W, off NW Iberian Peninsula, 450-500 m.

MNHN 7329: *Thalassa* U852, 44°12.0'N 08°34.0'W, off NW Iberian Peninsula, 615-654 m, d’Hondt Coll.

MNHN 7376: *Thalassa* Stn X323, 44°03.1’N 04°45.1’W, off N Iberian Peninsula, 650-590 m, d’Hondt Coll.

MNHN 7381: *Thalassa* Stn X345, 44°06.2’N 04°41.0’W, off N Iberian Peninsula, 525-550 m, d’Hondt Coll.

MNHN 7387: *Thalassa* X349, 44º07.1'N-44º06.9'N 04º43.8'W-04º44'W, off N Iberian Peninsula, 570-615 m, one colony on a rock, d’Hondt Coll.

MNHN 7621: same data as MNHN 7329.

MNHN 7628: *Thalassa* Stn W406, 43°55.5’N 05°44.0’W, off N Iberian Peninsula, 700-400 m, d’Hondt Coll.

MNHN 7629: same data as MNHN 7628.

MNHN 7630: same data as MNHN 7628.

MNHN 7632: same data as MNHN 7628.

MNHN 7629: *Thalassa* W406, 43º55.5'N 05º44.0'W, off N Iberian Peninsula, 400-700 m, one colony on a rock, d’Hondt Coll.

MNHN 7634: *Thalassa* W436, 44°11.8'N 08°40.7'W, off NW Iberian Peninsula, 499-600 m, d’Hondt Coll.

MNHN 7637: same data as MNHN 7628.

MNHN 7638: *Thalassa* Stn W390, 44°05.0’N 04°31.0’W, off N Iberian Peninsula, 1000-760 m, d’Hondt Coll.

MNHN 7639: *Thalassa* Stn W406, 43°55.5’N 05°44.0’W, off N Iberian Peninsula, 700-400 m, d’Hondt Coll.

MNHN 7640: same data as MNHN 6983.

MNHN 7641: same data as MNHN 6983

MNHN 7642: *Thalassa* Stn X340, 44°07.0’N 04°29.8’W, off N Iberian Peninsula, 860-910 m, d’Hondt Coll.

MNHN 7643: *Thalassa* Stn X340, 44°07.0’N 04°29.8’W, off N Iberian Peninsula, 860-910 m, d’Hondt Coll.

MNHN 7644: *Thalassa* Stn X341, 44°07.2’N 04°30.0’W, off N Iberian Peninsula, 840-800 m, d’Hondt Coll.

MNHN 7645: *Thalassa* Stn X356, 44°07.0’N 04°44.1’W, off N Iberian Peninsula, 600-550 m, d’Hondt Coll.

MNHN 7647: same data as MNHN 7222.

MNHN 7648: same data as MNHN 7222.

MNHN 7653: *Thalassa* Y440, 44°12.2'N 08°40.2'W, off NW Iberian Peninsula, 490 m, d’Hondt Coll. (Examined using SEM)

MNHN 7654: same data as MNHN 7653.

MNHN 8479: *Thalassa* Stn X347, 44°07.3’N 04°44.0’W, off N Iberian Peninsula, 640-910 m, d’Hondt Coll. (Examined using SEM)

MNHN 18350: same data as MNHN 7027.

MNHN IB-2013-564: *Thalassa* Stn Y433, 44°12.0'N 08°40.5'W, off NW Iberian Peninsula, 605-620 m. 04/08/1972. Harmelin Coll. (Examined using SEM)

MNHN IB-2013-576: same data as MNHN IB-2013-564

MNHN IB-2013-565: *Thalassa* Stn Y434, 44°12.0'N 08°40.8'W, off NW Iberian Peninsula, 605-620 m. 04/08/1972. Harmelin Coll. (Examined using SEM)

MNHN IB-2013-566: *Thalassa* Stn U807, 44°11.0'N 08°40.8'W, off NW Iberian Peninsula, 450-500 m. 18/10/1968. On stone. Harmelin Coll. (Examined using SEM)

MNHN IB-2013-577: same data MNHN IB-2013-566.

MNHN IB-2013-578: *Thalassa* Y428, 44°11.8'N 08°40.6'W, off NW Iberian Peninsula, 500-522 m, 04/09/1972. On stone. Harmelin Coll. (Examined using SEM)

MNHN IB-2013-579: *Noroit Seamount 1* DW5, 36°32.0'N 11°37.9'W, Gorringe Bank, 180 m, 22/09/1987. Harmelin Coll. (Examined using SEM)

MNHN IB-2013-580: *Noroit Seamount 1*, DW16. 36°31.1'N, 11°32.5'W. 255-265 m. Gorringe Bank. 24/09/1987. Harmelin Coll. (Examined using SEM)

MNHN IB-2013-581: same data as MNHN IB-2013-580.

MNHN IB-2013-582: *Noroit Seamount 1* DW21, 36°34.9'N 11°28.4'W, Gorringe Bank, 460-480 m, 24/09/1987. Harmelin Coll.

MNHN IB-2013-583: Chassefiere, Stn 010 11C, 37°22.25'N 11°34.60'E, Détroit Siculo-Tunisien, 400 m.One colony on coral. Harmelin Coll.

MNHN IB-2013-584: Sonne 41, 58 DC, 38°28.26’N 14°48.68’E, Lipari (Sicily), 700 m. A small ovicellate colony. Harmelin Coll. (Examined using SEM)

NHMUK 1985.1.2.70 A**:** *Sarsia* St 18, 47º25'N 06º30'W, N Bay of Biscay, 585-896 m, coral, 14 May 1958.

NHMUK 2012.2.8.8: *Thalassa* X352, 44º6.5'N 04º45.2'W, off N Iberian Peninsula, 545-580 m. (Examined using SEM)

MB37-000030: *Poseidon* Stn 13, 40°09.1'N 09°49.9'W, off N Portugal, 35-930 m, 21/11/1984. (Examined using SEM)

MB37-000037: *Poseidon* Stn 2, 41°09.3'N 09°20'W, off N Portugal, 800-900 m, 20/11/1984. (Examined using SEM)

*INDEMARES-Avilés* 0511, DR7, 43°52.7'N 05°54.4'W, off N Iberian Peninsula, 551 m, 06/05/2011, one small colony.

42°56'00''N 09°43'42''W, off NW Iberian Peninsula, 594 m, several colonies on a cetacean rib, Reverter-Gil Coll.

***Stephanotheca fayalensis*** (Calvet *in* Jullien & Calvet, 1903) n. comb.

MNHN 1014: *Talisman*, Stn 125, 38°29.1'N 28°37.33'W, between Pico and Faial (Azores), 13/08/1883, 80-115 m, one colony on shell affected by Bynesian decay, Calvet Coll.

MNHN 2809: a colony encrusting a bivalve shell (not in original label) (same information as MNHN 1014) [filed under, and occurring together with, *Cribrilina figularis* (Johnston)].

MNHN 3769: three colony fragments (same information as MNHN 1014).

MNHN 7547: *Biaçores* Stn 198, 37º50.5'N 25º04'W (Azores), 200-258 m; four discoidal colonies identified as *Schizomavella obsoleta*, d’Hondt Coll. (Examined using SEM)
